# Intensive lactation among women with recent gestational diabetes significantly alters the early postpartum circulating lipid profile: the SWIFT study

**DOI:** 10.1186/s12916-021-02095-1

**Published:** 2021-10-08

**Authors:** Ziyi Zhang, Mi Lai, Anthony L. Piro, Stacey E. Alexeeff, Amina Allalou, Hannes L. Röst, Feihan F. Dai, Michael B. Wheeler, Erica P. Gunderson

**Affiliations:** 1 Department of Physiology, Faculty of Medicine, University of Toronto, Toronto, Ontario Canada; 2 Department of Endocrinology, Sir Run Run Shaw Hospital, Zhejiang University, Zhejiang, Hangzhou China; 3 Division of Research, Kaiser Permanente Northern California, Oakland, California USA; 4 Donnelly Centre for Cellular and Biomolecular Research, University of Toronto, Toronto, Ontario Canada; 5 Metabolism Research Group, Division of Advanced Diagnostics, Toronto General Research Institute, Toronto, Ontario Canada; 6 Health Systems Science, Kaiser Permanente Bernard J. Tyson School of Medicine, Pasadena, USA

**Keywords:** Lactation, Lipid metabolism, Gestational diabetes mellitus, Type 2 diabetes risk

## Abstract

**Background:**

Women with a history of gestational diabetes mellitus (GDM) have a 7-fold higher risk of developing type 2 diabetes (T2D). It is estimated that 20-50% of women with GDM history will progress to T2D within 10 years after delivery. Intensive lactation could be negatively associated with this risk, but the mechanisms behind a protective effect remain unknown.

**Methods:**

In this study, we utilized a prospective GDM cohort of 1010 women without T2D at 6-9 weeks postpartum (study baseline) and tested for T2D onset up to 8 years post-baseline (n=980). Targeted metabolic profiling was performed on fasting plasma samples collected at both baseline and follow-up (1-2 years post-baseline) during research exams in a subset of 350 women (216 intensive breastfeeding, IBF vs. 134 intensive formula feeding or mixed feeding, IFF/Mixed). The relationship between lactation intensity and circulating metabolites at both baseline and follow-up were evaluated to discover underlying metabolic responses of lactation and to explore the link between these metabolites and T2D risk.

**Results:**

We observed that lactation intensity was strongly associated with decreased glycerolipids (TAGs/DAGs) and increased phospholipids/sphingolipids at baseline. This lipid profile suggested decreased lipogenesis caused by a shift away from the glycerolipid metabolism pathway towards the phospholipid/sphingolipid metabolism pathway as a component of the mechanism underlying the benefits of lactation. Longitudinal analysis demonstrated that this favorable lipid profile was transient and diminished at 1-2 years postpartum, coinciding with the cessation of lactation. Importantly, when stratifying these 350 women by future T2D status during the follow-up (171 future T2D vs. 179 no T2D), we discovered that lactation induced robust lipid changes only in women who did not develop incident T2D. Subsequently, we identified a cluster of metabolites that strongly associated with future T2D risk from which we developed a predictive metabolic signature with a discriminating power (AUC) of 0.78, superior to common clinical variables (i.e., fasting glucose, AUC 0.56 or 2-h glucose, AUC 0.62).

**Conclusions:**

In this study, we show that intensive lactation significantly alters the circulating lipid profile at early postpartum and that women who do not respond metabolically to lactation are more likely to develop T2D. We also discovered a 10-analyte metabolic signature capable of predicting future onset of T2D in IBF women. Our findings provide novel insight into how lactation affects maternal metabolism and its link to future diabetes onset.

**Trial registration:**

ClinicalTrials.gov NCT01967030.

**Supplementary Information:**

The online version contains supplementary material available at 10.1186/s12916-021-02095-1.

## Background

It is recommended by the World Health Organization (WHO) that mothers should exclusively breastfeed infants for the first 6 months following delivery for optimal maternal and infant health outcomes [1]. Despite this, breastfeeding rates remain well below 50% in infants younger than 6 months in most countries, irrespective of total income [2]. This may result from inability to breastfeed, lack of an extended period for paid maternity leave, inadequate information and social support, particularly in women with high pre-pregnancy obesity, professional career demands, and older or younger maternal age [1, 3]. This is concerning as lactation is a postpartum behavior associated with several beneficial effects including reduced infant morbidity and mortality, preventing breast and ovarian cancer in mothers, as well as having a negative association with their risk of developing future diabetes and other cardiovascular diseases in mid to later life [2, 4–9].

In prospective studies of lactation and incident type 2 diabetes (T2D), 5 or more months of lactation was associated with up to 50% reduction in the relative risk of future T2D [7, 8]. A meta-analysis of 206,204 women reported that breastfeeding for 12 months or longer was associated with a relative risk reduction of only about 30% for incident T2D (pooled odds ratio 0.70; 95% CI, 0.62-0.78; *p* < 0.001) [10]. Other large epidemiologic studies followed women starting at older ages and reported much weaker protective effects of lactation on future T2D (3-15% lower relative risk for each year of lactation) [11–13]; however, these studies were limited by self-report of diabetes and inability to account for GDM history, potentially biasing estimates towards the null.

GDM is a common disorder that occurs in approximately 10% of all pregnancies [14–16]. Women who develop GDM have several fold higher risk of developing T2D during mid to later life compared to non-GDM women [17, 18]. The Coronary Artery Risk Development Study in Young Adults (CARDIA), a biracial cohort with black and white women, found that 6 or more months of lactation was associated with up to 50% relative reduction in the incidence of T2D in the 30-year follow-up [7]. The Study of Women, Infant Feeding, and Type 2 Diabetes after GDM Pregnancy (SWIFT), a racially and ethnically diverse cohort, found that increased lactation intensity and duration for 2 or more months was associated with a graded 34-57% relative risk reduction in the 2-year incidence of T2D after GDM pregnancy, independent of prenatal glucose intolerance and perinatal outcomes [8]. Overall, this body of evidence suggested a significant association between intensive lactation and reduced risk of incident T2D. However, the mechanisms underlying these observed beneficial effects of lactation on future diabetes onset remain unknown.

Lipids play an important role in the pathogenesis of T2D. It has been demonstrated that elevated circulating triacylglycerol (TAG) and decreased high-density lipoprotein (HDL) cholesterol are directly associated with T2D [19, 20]. Some studies have focused on lipid metabolism during lactation and demonstrated that intensive lactation after a GDM pregnancy was associated with higher HDL-cholesterol and lower fasting TAGs [21, 22]. Similarly, a longitudinal study showed higher HDL-cholesterol persisted in women who had lactated for 3 months or longer [23]. In a previous study applying targeted metabolomics, Much et al. found that lactation > 3 months in women with previous GDM pregnancy was associated with a higher total lysophophatidylcholine/total phosphatidylcholine ratio at 30 and 120 min during a 2-h 75-g oral glucose tolerance test (OGTT) within 3.6 years postpartum [24]. They also observed lower branched-chain amino acid concentrations at 30 min within 0.7 years postpartum in this group [24]. Despite these intriguing findings, only a limited number of lipids (90 glycerophospholipids and 15 sphingolipids) were analyzed, warranting a more comprehensive and in-depth analysis of lipid metabolism associated with lactation.

Currently, the recommended test to reclassify glucose tolerance after GDM pregnancy is a 2-h 75-g OGTT performed at 6 to 12 weeks postpartum followed by testing for diabetes every 1-3 years via fasting plasma glucose (FPG) and 2-h OGTT [25]. However, the accuracy of a 2-h 75-g OGTT for prediction of future T2D is an unexceptional ~ 65% [26–28]. A more convenient and accurate predictive test is needed to assess glucose tolerance and predict future T2D following GDM pregnancy. Specific metabolites revealed by discovery-based metabolomics in addition to glucose were reported to facilitate the early prediction of T2D in the general population [29]. Therefore, a metabolite-based signature at early postpartum may contribute to effective prediction of the future onset of T2D.

In the present study, we aim to determine the association between intensive lactation and metabolic profiles in women with recent GDM pregnancy and subsequently use selected metabolites to predict future risk of T2D.

## Methods

### Design of SWIFT cohort

The Study of Women, Infant Feeding, and Type 2 Diabetes after GDM Pregnancy (SWIFT) is a prospective, longitudinal clinical research study that enrolled 1035 racially and ethnically diverse (Non-Hispanic white, 23%; Hispanic, 31%; Asian, 36%; Black, 8%; other, 2%) women (aged 20–45 years) with GDM (via 3-h 100-g OGTTs, based on Carpenter-Coustan’s criteria [30]) who delivered a singleton, live-born infant at or after 35 weeks of gestation at KPNC hospitals from September 2008 to December 2011. This clinical trial can be located at ClinicalTrials.gov with identifier NCT01967030. Details of study design and setting, study sample size, inclusion/exclusion criteria, study procedures, assessment of the main exposure, and other detailed information on data collection methodologies have been described elsewhere [31]. Briefly, participants were recruited from 13 KPNC medical centers/office facilities and pregnant women with a diagnosis of GDM were identified from electronic medical records and added into the study recruitment tracking system on a weekly basis. After pre-screening for eligibility by trained research staff, potential participants were invited to participate in the research study, and those interested were scheduled for an in-person research examination at 6–9 weeks postpartum (study baseline). At baseline, the 1035 participants were administered a 2-h 75-g OGTT to classify glucose tolerance status and measure plasma glucose and insulin. Additionally, lactation intensity and duration were evaluated, and other assessments were conducted under research protocols. Three in-person examinations were additionally performed annually for up to 2 years post-baseline, at which 2-h 75-g OGTTs and research assessments were performed. At each exam, plasma samples (fasting and 2-h timepoint) were collected during the 2-h 75-g OGTT to reclassify glucose tolerance. New diagnoses of T2D since baseline were also obtained electronically from medical records up to 8 years post-baseline. T2D was diagnosed via the ADA criteria [32].

Frequency and amount of breastmilk feeding (including expressed breast milk bottle feeding) and formula feeding for each woman were assessed by trained research staff via telephone calls, mailed feeding diaries, questionnaires during in-person visits, and mailed monthly surveys from birth to 12 months post-delivery as previously described [33]. Based on this information, breastfeeding behavior measurements within each month were operationalized as breastfeeding intensity and duration ratio (quantitative methodology), which was calculated as the number of breast milk feeds (on average in 24 h) divided by the total number of all liquid feeds (on average in 24 h) during the past 7 days to yield a score with a range from 0 to 1 as described by Piper et al. [34]. A score of “1” represents exclusive breastfeeding and a score of “0” represents exclusive formula feeding, with fractional scores representing levels of lactation intensity. We then constructed a summary score (LIR) for the baseline measures by adding the intensity ratios from delivery to 2 months postpartum to obtain a lactation score at study baseline, ranging from 0 to 2. We set a 2-month LIR score of 1.45 as the cut-off value to categorize the women into intensive breastfeeding (IBF) or intensive formula/mixed feeding (IFF/Mixed) groups. Women with LIR score ≥ 1.45 were defined as IBF, whereas women with LIR score < 1.45 were considered as IFF/Mixed. These two groups reflect different levels of lactation intensity during the first 2 months. The 1.45 cut-off value was achieved by 70–100% of feedings being breastmilk for each month. At least 27% of women in this category had exclusively breastfed for 2 months, and at least 96% had breastfed at 80% for 2 months.

Fasting plasma samples obtained from 2-h 75-g OGTTs at baseline and at follow-up exams were processed, aliquoted and stored in -70 °C freezers. The aliquoted plasma samples were then transported from the study sites to the KPNC Regional Laboratory and then further to the Division of Research (DOR) for storage at -70 °C. Upon arrival at the DOR research clinic, cryogenic vials were scanned into the SWIFT biospecimen database.

### Targeted metabolomic profiling and data pre-processing

The metabolomics data was obtained from our recently published paper where the details of metabolomics analysis were described [75]. Metabolomic profiling was applied on fasting plasma samples from 350 participants (216 IBF vs. 134 IFF/Mixed) at baseline and 303 participants (188 IBF vs. 115 IFF/Mixed) at follow-up (not all participants delivered follow-up samples). In this study, the AbsoluteIDQ p180 kit (Biocrates Life Sciences, Innsbruck, Austria), which quantifies broad metabolite spectrum and reflects diverse physiological processes, was applied to measure a total of 188 metabolites according to the manufacturer’s instructions using mass spectrometry-based techniques. These 188 analytes included 21 amino acids (AA), 40 acylcarnitine (AC), 21 biogenic amines (BA), 1 monosaccharide, 90 glycerophospholipids, and 15 SMs. For the data pre-processing, metabolites with missing values > 40% were excluded from the study, which reduced the total number of metabolites from 188 to 141 at baseline and from 188 to 145 at follow-up. The remaining missing values were imputed with half of the limit of detection (LOD) value of each metabolite. The value of each metabolite was normalized within the total value of each sample, followed by log-transformation and mean-centric scaling; distribution of data was then checked. Afterwards, dataset qualities for further bioinformatic analysis were examined for potential confounding factors and the existence of class separation between two groups by performing PCA and PLS-DA along with empirical Bayes estimation (1000 random permutations in this situation). A robust separation between two groups was confirmed by empirical *p* value < 0.05. The data pre-processing was performed on the online platform MetaboAnalyst 4.0 (https://www.metaboanalyst.ca/home.xhtml) [35].

### Targeted lipidomic profiling and data pre-processing

The lipidomics data was obtained from our recently published paper where the details of lipidomics analysis were described [36]. Baseline fasting plasma samples obtained from 350 women (216 IBF vs. 134 IFF/Mixed) were subjected to targeted-lipid profiling performed by Metabolon, Inc. (Morrisville, NC) based on gas chromatography–mass spectrometry and liquid chromatography–mass spectrometry techniques. The targeted lipidomic profiling allowed the measurements of 1008 lipid species from 15 classes as well as 296 fatty acids. The 1008 lipid species include 26 CE, 26 FFA, 26 MAG, 59 DAG, and 493 TAG from the neutral lipid group; 26 LPC, 26 LPE, 140 PC, 216 PE, and 28 PI from the phospholipid group; 12 CER, 13 DCER, 12 HCER, 12 LCER, and 12 SM from the sphingolipid group. For the data pre-processing, lipids with > 5% missing values were excluded at baseline, leaving 818 out of 1008 lipid species for the bioinformatic analysis. Other data pre-processing including missing value imputation, data normalization, and transformation, PCA and PLS-DA analyses were performed as stated above.

### Cross-sectional analyses at baseline and follow-up: differential expression analysis

At baseline, we selected 350 women (216 IBF vs. 134 IFF/Mixed) for the cross-sectional analysis using both lipidomics and metabolomics data. At follow-up, 303 of the 350 women (188 IBF vs. 115 IFF/Mixed) were used for the cross-sectional analysis using metabolomics data. As this is a secondary analysis of previous case-control data [36, 75], the data cannot be treated as a cohort in which the original case-control design is ignored. We took the nested case-control study design into consideration and performed secondary data analysis using a weighted regression model [37, 38] to detect differentially expressed metabolites/lipids between IBF and IFF/Mixed women at baseline and follow-up. The sampling probability was calculated for each individual in the case-control study. Sampling weights are calculated as the inverse of the sampling probability. Individual lipids/analytes along with calculated weights were subjected to generalized linear models (GLMs) and Type III ANOVA tests were performed. As pre-pregnancy BMI was significantly different (*p* = 0.02, Table 1) between the two groups, these models were adjusted for pre-pregnancy BMI. Afterwards, false discovery rate (FDR) was calculated using Benjamini-Hochberg method for multiple comparison. Metabolites and lipid species with FDR value < 0.05 were considered to be significantly differentially expressed between IBF and IFF/Mixed. Lipid species were further grouped and analyzed according to the number of carbon atoms, the number of double bonds, and fatty acid composition. The 350 women were then stratified based on the onset of future T2D; 171 women progressed to T2D during the follow-up whereas 179 women did not. In the future T2D subgroup, 98 women were IBF and 73 women were IFF/Mixed. In the no T2D subgroup, 118 women were IBF and 61 women were IFF/Mixed. We also stratified our analytic samples based on the status of glucose tolerance at baseline according to the 2-h 75-g OGTT results and found that 180 women had IFG or IGT, whereas 170 women exhibited NGT. In the IFG/IGT subgroup, 98 women were IBF and 82 women were IFF/Mixed. In the NGT subgroup, 118 women were IBF and 52 women were IFF/Mixed. Differential lipids between IBF and IFF/Mixed in each subgroup (Future T2D, No T2D, IFG/IGT, NGT) were identified as stated above. A cut-off of FDR < 0.05 was used for significance. The analyses were performed in open-source software RStudio (Version 1.2.5033).

### Longitudinal analysis of metabolites from baseline to follow-up

Metabolomics data from 303 samples (188 IBF and 115 IFF/Mixed) collected both at baseline and follow-up were included in the longitudinal analysis. Metabolites with missing values > 40% at either baseline or follow-up were excluded at both time points, leaving 130 analytes for further analysis. Data normalization and transformation was performed as stated above. Batch effects between baseline and follow-up were corrected by using the same internal control in these two batches. To further assess the difference in dynamic change of each metabolite between the IBF group and IFF/Mixed group, a mixed effect model was fitted for each metabolite and Type III ANOVA tests were carried out in SPSS Statistics (Version 26, IBM, Armonk, NY). In the mixed effect model, group (IBF or IFF/Mixed), time (baseline or follow-up), and their interactions were included as fixed effects, whereas patient ID was included as a random effect. Total lactation duration was adjusted during the longitudinal analysis. Next, *p* values were corrected by using Benjamini-Hochberg method, and a cut-off of FDR value < 0.05 was considered for significance.

### Pathway analysis and upstream transcription factors prediction

Lipidomics data from 350 samples at baseline were used for the pathway analysis and master regulon prediction. Using the KEGG (Kanehisa Laboratories, Kyoto, Japan) database, the differentially expressed lipid species, including upregulated and downregulated lipids, were subjected to pathway analysis, respectively. The KEGG pathway analysis was performed on MetaboAnalyst 4.0. Further, the significantly differentially expressed lipid species (FDR < 0.05) between IBF and IFF/Mixed at baseline were subjected to an online platform MetaBridge (https://www.metabridge.org/) to further identify all of the reactions in which the lipids participate in and all of the potential genes that are involved in these reactions [39]. The gene list generated by MetaBridge was subjected to a computational method called iRegulon to identify the master regulons of these genes using cis-regulatory sequence analysis [40]. A mapping of the network between regulons and their downstream targeted genes was generated by using Cytoscape (Cytoscape Consortium, San Diego, CA, USA).

### Prediction analysis

Sixty-nine significantly differentially expressed analytes (FDR < 0.05, Additional file 1: Table S13) between the future T2D (*n* = 98) and no T2D (*n* = 118) women in the IBF group were subjected to the prediction analysis. To build and evaluate the generated predictive model, we randomly selected 25 future T2D and 25 no T2D subjects as the hold-out testing set. The remaining participants (73 future T2D and 93 no T2D) were used as the training set. During the prediction analysis, the training set was randomly down-sampled to 73 future T2D and 73 no T2D in a case-control balanced set. Then, random forest classification was used to identify the predictive variables and generate a prediction model (package randomForest in R). The generated model was further applied on the hold-out testing set to evaluate the predictive performance. This process was repeated 100 times and the top 30 variable importance (VIP) analytes were recorded at each time (Additional file 1: Figure S7). Then the top 10 analytes with the highest frequency of appearance in the 100 times’ top VIP lists were chosen as the final predictive signature to generate the predictive model (Additional file 1: Figure S7). Model evaluation in the hold-out testing set was presented as area under the curve (AUC), accuracy, F1-score, precision, sensitivity, and specificity. Instead of presenting the best model, we reported the median values of the model parameters (AUC, accuracy, F1-score, precision, sensitivity, and specificity) to avoid potential bias and overfitting. All prediction analyses were performed in the RStudio (Version 1.2.5033).

## Results

### Overview of cohort and study design

**Fig. 1:**
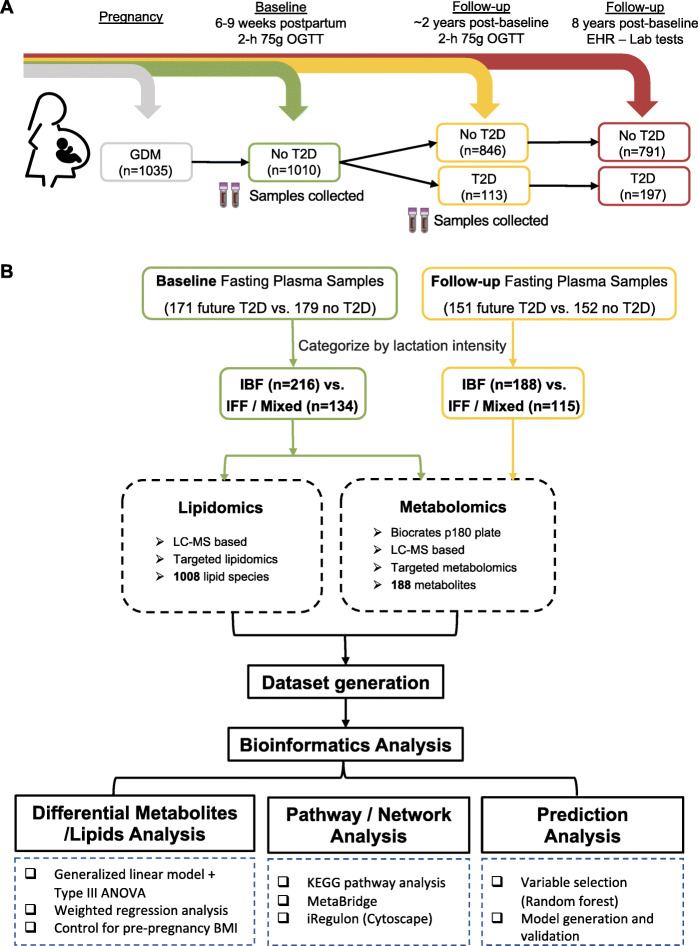
Overview of the study of women, infant feeding, and type 2 diabetes (SWIFT) cohort and study design. The SWIFT cohort is a prospective longitudinal research cohort of 1035 women with recent GDM enrolled at 6–9 weeks postpartum (baseline) from 2008 to 2011. There were 1010 out of 1035 women who were free of diabetes confirmed by a 2-h 75-g OGTT at baseline. Women were followed up annually via 2-h 75-g OGTT up to 2 years postpartum (*n* = 959 women), and their electronic medical records (EHR) were utilized to obtain additional clinical diagnoses of diabetes from baseline to 8 years post-baseline (till January 16, 2019). Fasting plasma samples were obtained at both baseline and follow-up during 2-h 75-g OGTTs. Baseline analysis included a total of 350 women (216 IBF vs 134 IFF/Mixed) whose stored fasting plasma samples were collected and were subjected to targeted metabolomics and lipidomics profiling. Follow-up and longitudinal analyses included 303 of the 350 women (188 IBF vs 115 IFF/Mixed) whose fasting plasma samples were subject to targeted metabolomics profiling. Targeted metabolomics allowed the detection of 188 analytes and targeted lipidomics allowed detection of 1008 lipid species based on liquid chromatography–mass spectrometry (LC-MS) technique. The generated dataset was then subjected to data pre-processing and bioinformatic analysis

In the SWIFT cohort (1035 women in total), women diagnosed with diabetes at baseline (*n* = 21), 2 dropouts, and 2 ineligible women were excluded from the follow-up. Of 1010 participants without diabetes at baseline, 959 women attended in-person research exams at 1 and/or 2 years post-baseline (follow-up), which included 2-h 75-g OGTTs to evaluate glucose tolerance status. Additionally, we supplemented this testing with clinical diagnoses of diabetes from Kaiser Permanente Northern California (KPNC) electronic medical records up to 8 years post-baseline (Fig. 1A).

We selected a subset of 350 women (171 future diabetes cases vs. 179 no subsequent diabetes controls) from the larger SWIFT cohort using a nested case-control design in which women were matched on age, prepregnancy BMI, and race/ethnicity as previously described [36]. In this current study, these 350 women were classified as either intensive breastfeeding (IBF, *n* = 216) or intensive formula or mixed feeding (IFF/Mixed, *n* = 134) according to their 2-month lactation intensity/duration ratio (LIR) score at study baseline. Fasting plasma samples were collected during the 2-h 75-g OGTTs performed at baseline and 1–2 years post-baseline to evaluate metabolic changes. Targeted metabolomics and lipidomics were applied on fasting plasma samples collected at baseline in 350 women (216 IBF vs. 134 IFF/Mixed), and targeted metabolic profiling was also performed on fasting samples collected at follow-up in 303 women (188 IBF vs. 115 IFF/Mixed) (Fig. 1B). We then applied bioinformatics analysis to identify lactation intensity-associated metabolites/pathways and generate a predictive signature for future T2D risk (Fig. 1B).

### Clinical characteristics of participants

Clinical, sociodemographic, and biochemical data in prenatal and postpartum periods for the 350 participants are summarized in Table 1. In the prenatal period, there were no statistically significant differences in age, race/ethnicity, prenatal 3-h 100-g OGTT sum of *z*-scores, and type of GDM treatment between IBF and IFF/Mixed groups (*p* > 0.05). Pre-pregnancy BMI was slightly higher in IFF/Mixed women compared to IBF women (Mean ± SD: 34.1 ± 8.6 vs. 32.2 ± 6.8 kg/m^2^; *p* = 0.02). At 6-9 weeks postpartum, the IBF group had lower fasting plasma glucose (FPG) (*p* < 0.001), fasting insulin (*p* < 0.001), HOMA-IR (*p* < 0.001), and HOMA-β (*p* = 0.006) than the IFF/Mixed group. Fewer IBF women had impaired glucose tolerance compared to IFF/Mixed women at baseline (*p* = 0.004). There was no difference in 2-h plasma glucose (2 h-PG) (*p* = 0.22) between the two groups. During the follow-up, there was no significant difference in the number of women with incident diabetes between IBF and IFF/Mixed groups. There was no significant difference in LIR score between women who developed future diabetes and those who did not.

**Table 1:** Clinical characteristics of women with GDM in the SWIFT cohort

	IBF (***n*** = 216)	IFF/Mixed (***n*** = 134)	***P*** value
**Prenatal characteristics**			
Age, years, mean (SD)	34.1 (4.7)	33.6 (5.1)	0.38
Pre-pregnancy BMI, kg/m^2^, mean (SD)	32.2 (6.8)	34.1 (8.6)	**0.02**
Race, *n* (%)			0.35
Non-Hispanic white	35 (16.2%)	23 (17.1%)	
Asian	68 (31.5%)	38 (28.4%)	
Non-Hispanic black	18 (8.3%)	19 (14.2%)	
Hispanic	93 (43.1%)	52 (38.8%)	
Others	2 (0.9%)	2 (1.5%)	
*Z*-score sum of 3-h 100-g OGTT during pregnancy, mean (SD)	0.5 (3.0)	0.6 (2.7)	0.81
Treatment for GDM, *n* (%)			0.88
Diet	124 (57.4%)	78 (58.2%)	
Oral medications/insulin	92 (42.6%)	56 (41.8%)	
**Baseline characteristics at 6-9 weeks postpartum (study baseline)**			
2-h 75-g OGTT			
FPG, mmol/l, mean (SD)	96.3 (9.4)	100.4 (9.9)	**< 0.001**
2 h-PG, mmol/l, Mean (SD)	118.6 (31.0)	122.7 (29.2)	0.22
Fasting insulin, pmol/l, median (IQR)	21.3 (15.2–31.4)	29.5 (19.6–41.1)	**< 0.001**
HOMA-IR, median (IQR)	5.0 (3.5–7.7)	7.5 (4.7–10.6)	**< 0.001**
HOMA-β, median (IQR)	238.9 (172.1–354.6)	282.0 (202.0–380.2)	**0.006**
Status of glucose tolerance, *n* (%)			**0.004**
NGT	118 (54.6%)	52 (38.8%)	
IFG/IGT	98 (45.4%)	82 (61.2%)	
2-month LIR score, Median (IQR)	1.98 (1.86–2.0)	0.53 (0.25–1.06)	**< 0.001**
No T2D 2-month LIR score	1.98 (1.88–2.0)	0.50 (0.25–1.0)	**< 0.001**
Future incident T2D 2-month LIR score	1.98 (1.81–2.0)	0.55 (0.23–1.06)	**< 0.001**
**Follow-up characteristics**			
Future T2D status up to 8 years post-baseline, *n* (%)			0.098
Future T2D	98 (45.4%)	73 (54.5%)	
No T2D	118 (54.6%)	61 (45.5%)	
Person-time of follow-up, months, mean (SD)	53.4 (32.2)	53.0 (33.6)	0.92

Data are presented as mean (SD) for continuous variables that are approximately normally distributed. Data are presented as median (IQR) for continuous variables with asymmetrical distributions. Chi-square test was used for categorical variables (*n*, %), *t*-test was used for continuous variables (mean, SD), and Mann-Whitney *U* test was used for continuous variables (median, IQR). *IBF*, intensive breastfeeding; IFF/Mixed, intensive formula feeding or mixed feeding; *BMI*, body mass index; *OGTT*, oral glucose tolerance test; *GDM*, gestational diabetes mellitus; *FPG*, fasting plasma glucose; *2 h-PG*, 2-h postload plasma glucose; *HOMA-IR*, homeostatic model assessment for insulin resistance; *HOMA-β*, homeostatic model assessment for beta cell function; *NGT*, normal glucose tolerance; *IFG*, impaired fasting glucose; *IGT*, impaired glucose tolerance; *LIR*, lactation intensity/duration ratio; *T2D*, type 2 diabetes

### Metabolic changes associated with lactation intensity in cross-sectional (baseline and follow-up) and longitudinal analyses

**Fig. 2:**
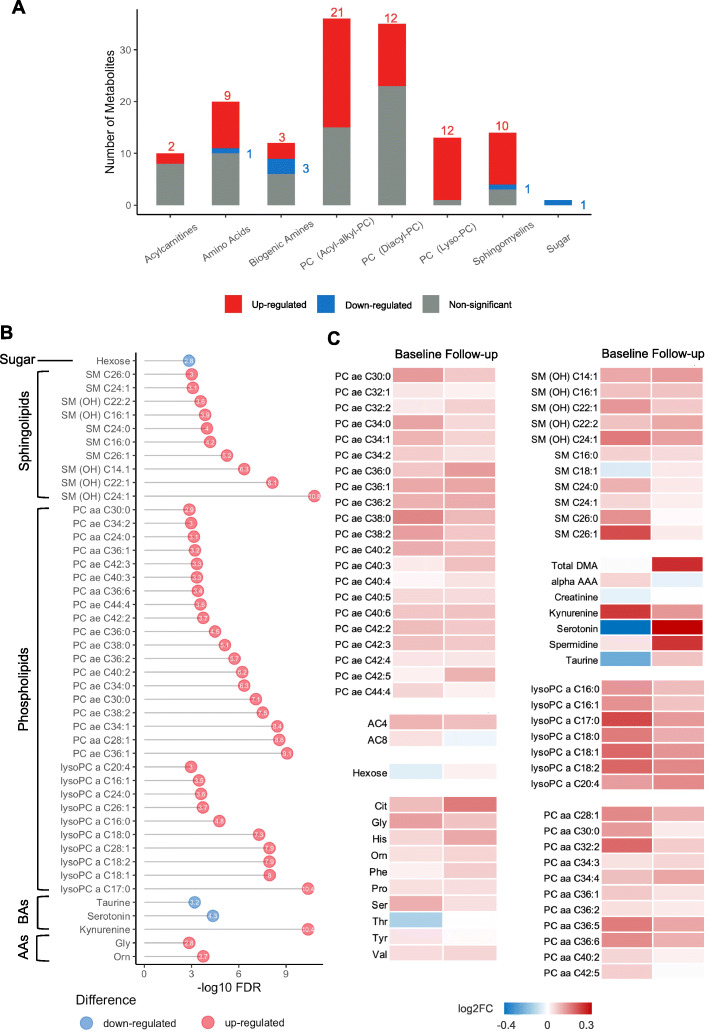
Metabolites associated with intensive lactation at baseline and follow-up. **A** Number of differentially expressed metabolites associated with lactation intensity in each class. Red indicates significantly upregulated metabolites whereas blue indicates significantly downregulated, and grey denotes no significant change. Significance was indicated by FDR < 0.05. **B** Bubble plot showing significantly differentially expressed metabolites (FDR < 0.002) associated with lactation intensity in each class at baseline. Red points indicate upregulation and blue points denote downregulation. The value of -log10 FDR of each metabolite is shown in the bubble. **C** Comparison of the log2 FC of the significantly differentially expressed metabolites identified at baseline and follow-up

At baseline, normality of the metabolomics dataset was checked (Additional file 1: Figure S1A). Principal component analysis (PCA) and partial least squares-discriminant analysis (PLS-DA) indicated separability between the IBF and IFF/Mixed women (Additional file 1: Figure S1B to S1D). To further identify the differentially expressed metabolites between IBF and IFF/Mixed groups, each metabolite was subjected to generalized linear model (GLM), and Type III ANOVA was carried out to evaluate significance. Calculated weights for cases (future T2D) and controls (no T2D) were applied in the model to account for the original nested case-control design. These models were adjusted for pre-pregnancy BMI. Between IBF and IFF/Mixed groups, 75 metabolites were identified that were differentially expressed with statistical significance (FDR < 0.05), including 69 upregulated analytes and 6 downregulated analytes (Fig. 2A, Additional file 1: Figure S1E and Table S1). All differential metabolites with FDR < 0.002 were summarized and shown in Fig. 2B. Strikingly, the majority of differentially expressed metabolites were clustered in the phospholipids and sphingolipids and were higher in IBF women compared to IFF/Mixed women (Fig. 2B).

At follow-up, the IBF group and IFF/Mixed group could not be distinguished in PLS-DA analysis (*p* = 0.859) (Additional file 1: Figure S1F to S1G), and only 3 metabolites (Histidine, Citrulline and total-DMA) were found to be differentially expressed between the two groups (Additional file 1: Table S2). Further, all significantly differentially expressed analytes that we identified at baseline or follow-up were compared in Fig. 2C. Most differentially regulated analytes maintained their trends at follow-up. However, these analytes did not show significant differences at follow-up, suggesting the effects of lactation intensity during concurrent lactation on metabolites were remarkably lessened after cessation of lactation. Moreover, we stratified these 303 women into no future T2D (*n* = 152), short-term T2D (T2D onset at 1-2 years post-baseline, *n* = 102) and long-term T2D (T2D onset > 2 years post-baseline, *n* = 49) subgroups. In women with no future T2D, the 3 metabolites stated above were still found to be differentially expressed between IBF and IFF/Mixed, whereas no differential metabolites were identified between IBF and IFF/Mixed in short- and long-term T2D subgroups.

To further determine whether postpartum lactation has a persistent impact on long-term maternal metabolism, we performed a longitudinal analysis to examine the dynamic changes of each metabolite within each individual between IBF and IFF/Mixed groups. A total of 303 women (188 IBF and 115 IFF/Mixed) who had metabolomics data at both baseline and follow-up were included in the longitudinal analysis. No metabolites were significantly changed from baseline to follow-up between IBF group and IFF/Mixed group (Additional file 1: Figure S2). We further stratified these 303 women into no T2D (*n* = 152), short-term T2D (*n* = 102), and long-term T2D (*n* = 49) subgroups and performed longitudinal analysis in each subgroup. No metabolites were found to be significantly changed over the time period.

### Lipid species changes associated with lactation intensity at baseline

**Fig. 3:**
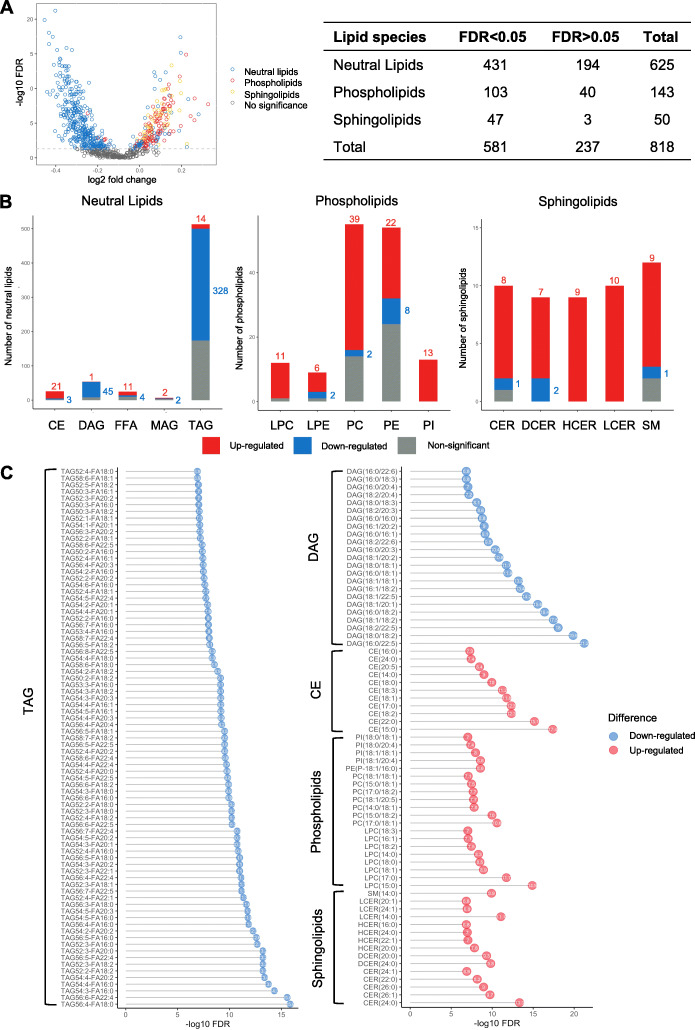
Lipid species associated with intensive lactation at baseline. **A** Volcano plot showing log2 FC against -log10 FDR of 818 lipid species measured at baseline in the IBF group compared to the IFF/Mixed group. Blue points indicate significantly differentially expressed neutral lipids, red denotes phospholipids, and yellow indicates sphingolipids. Grey points indicate no significant change. Table shows the sum number of differentially/non-differentially expressed lipid species in three lipid groups. Significance was indicated by FDR < 0.05. **B** Number of lactation intensity-associated metabolites in 15 lipid classes. Red indicates upregulated metabolites whereas blue indicates downregulated, and grey denotes no significant change. Significance was indicated by FDR < 0.05. **C** Bubble plot showing the top 150 (organized by FDR value) most significantly differentially expressed lipid species associated with lactation intensity. Red points indicate upregulation and blue points denote downregulation

The metabolomics showed that the majority of differential metabolites were clustered in the lipid class. To further explore these findings, we utilized lipidomics covering a wide-spectrum of lipid species (1008 lipid species from 15 classes along with 296 fatty acids) as previously described [36] to assess the lipid changes associated with lactation intensity at baseline among 350 women (216 IBF vs. 134 IFF/Mixed). A total of 818 lipid species were included in the final bioinformatic analysis. Normality of dataset was checked (Additional file 1: Figure S3A). PCA and PLS-DA analyses showed a distinct separation between the two groups which was not due to a random effect (Additional file 1: Figure S3B to S3D). Of the 818 lipid species, remarkably, 581 species were significantly associated with lactation intensity at baseline (FDR < 0.05), with 183 lipids upregulated and 398 downregulated in the IBF compared to IFF/Mixed group (Fig. 3A, B, Additional file 1: Table S3). These 581 differentially expressed lipids were composed of 431 neutral lipids, 103 phospholipids, and 47 sphingolipids (Fig. 3A). Of the 398 downregulated lipids, 328 were from TAG class while 45 were from diacylglycerol (DAG) class (Fig. 3B). In contrast, of the 183 upregulated lipids, 91 were phospholipids (11 from lysophosphatidylcholine (LPC) class, 6 from lysophosphatidylethanolamine (LPE) class, 39 from phosphatidylcholine (PC) class, 22 from phosphatidylethanolamine (PE) class, 13 from phosphatidylinositol (PI) class), 43 were sphingolipids (8 from ceramide (CER) class, 7 from dihydroceramide (DCER) class, 9 from hexosylceramide (HCER) class, 10 from lactosylceramide (LCER) class and 9 from sphingomyelin (SM) class), and 49 were neutral lipids (21 were from cholesterol ester (CE) class, one from DAG class, 11 from free fatty acid (FFA) class, 2 from monoacylglycerol (MAG) class, and 14 from TAG class) (Fig. 3B). Notably, 64% (328 out of 513) of measured TAGs and 83% (45 out of 54) of measured DAGs were significantly downregulated in the IBF women, suggesting women with higher lactation intensity at baseline had lower levels of circulating TAGs and DAGs than women who were intensive formula feeding or mixed feeding, which is consistent with clinical biochemistry measurements [22]. More strictly, we showed the top 150 most significantly differentially expressed lipid species (Fig. 3C). Among these lipid species, 81 TAGs and 23 DAGs were consistently negatively associated with lactation intensity at baseline. In contrast, 15 sphingolipids, 20 phospholipids, and 11 CEs were consistently positively correlated with intensive lactation (Fig. 3C). We also stratified the cohort according to the glucose tolerance status at baseline and found that the changes of lipid profiles observed above were maintained in both normal glucose tolerant (NGT) women and those with impaired glucose metabolism (impaired fasting glucose/impaired glucose tolerance, IFG/IGT) (Additional file 1: Figure S4).

Furthermore, based on the amount of formula and breastmilk feeding, we categorized the women into four groups as we reported previously; (i) exclusive BF (no formula or other feeds); (ii) mostly BF(≤ 6 oz of formula per 24 h); (iii) mostly FF (> 17 oz of formula per 24 h), mixed (7-17 oz of formula per 24 h) or inconsistent feeding; and (iv) exclusive FF (formula only) [8]. We compared the lipid profiles in these four groups (exclusive BF *n* = 62, mostly BF *n* = 131, mostly FF/Mixed *n* = 91, and exclusive FF *n* = 66) to further examine whether there was a dosage effect of lactation intensity on lipid profiles. A total of 260 lipid species were significantly differentially expressed among the four groups. TAGs/DAGs were negatively associated with lactation intensity, whereas phospholipids/sphingolipids were positively associated with lactation intensity. Moreover, there was a dosage effect of lactation intensity associated with lipid changes. (Additional file 1: Figure S5).

Additionally, we performed an extreme analysis by comparing the exclusive BF group with the exclusive FF group. A total of 267 lipid species were found to be significantly changed between the two groups. Similar to what we observed in the IBF/IFF or mixed groups, significant lower TAGs/DAGs but higher phospholipids/sphingolipids were detected in the exclusive BF group compared to exclusive FF group (Additional file 1: Figure S6).

### Characterization of lipid structure and composition associated with lactation intensity

**Fig. 4:**
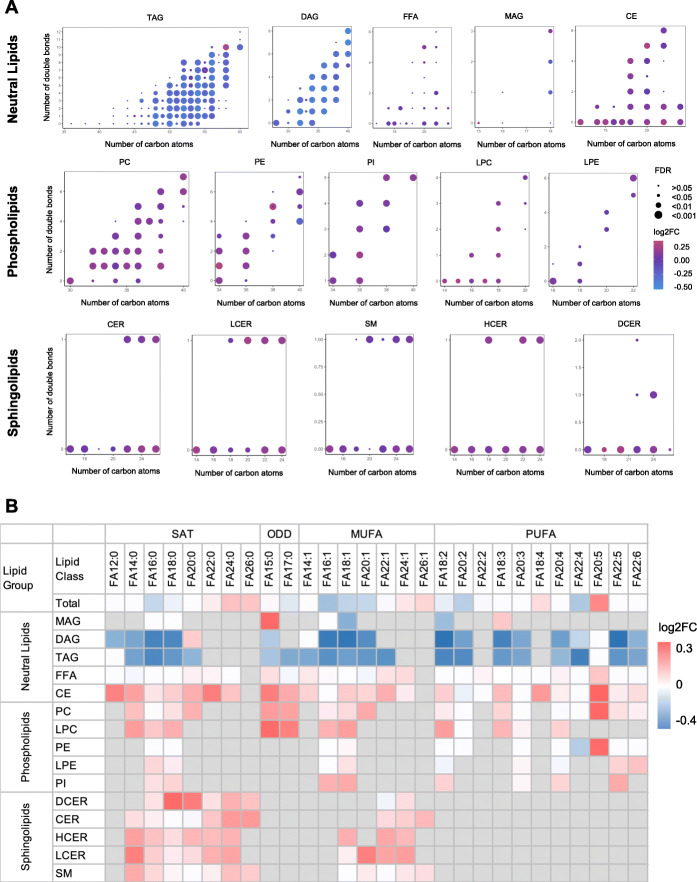
Characterization of lipid structure associated with intensive lactation at baseline. **A** Association between lactation intensity groups and lipid structure including the number of carbon atoms and double bonds in each lipid species from 15 lipid classes. Log2 FC of each lipid species are indicated with dots, with color of dots indicating log2 FC value, and dot size denoting significance by FDR value. **B** Association between lactation intensity and fatty acid composition in 15 lipid classes. Red and blue color indicate log2 FC with significance (FDR < 0.05), whereas white color denotes no significant difference, and grey color indicates not detected

In addition to lipid species, we also examined number of carbon atoms and double bonds in lipidomic profiling to gain insight into whether intensive lactation affected composition and configuration of lipids. The TAGs measured in this study possessed carbon atoms ranging from 35 to 60, and double bonds ranging from 0 to 12. Significantly downregulated TAGs in IBF women were clustered in the range of carbon atoms 50–56, especially those with even carbon atoms (50, 52, 54, and 60) (Fig. 4A). Similarly, DAGs with an even number of carbon atoms (32, 34, 36, 38, and 40) were significantly negatively associated with intensive lactation at baseline (Fig. 4A). We did not identify specific patterns in other lipid classes (Fig. 4A). As for the total FAs, most long-chain fatty acid (FA 16:0, FA 16:1, FA 17:0, FA 18:0, FA 18:1, FA 18:2, FA 20:1, and FA 20:2) were significantly downregulated in IBF women, whereas most very long-chain fatty acids (FA 22:0, FA 24:0, FA 24:1, FA 26:0, and FA 26:1) were upregulated. No changes were observed in medium-chain fatty acids (Fig. 4B and Additional file 1: Table S4).

### Metabolic pathways associated with lactation intensity at baseline

**Fig. 5:**
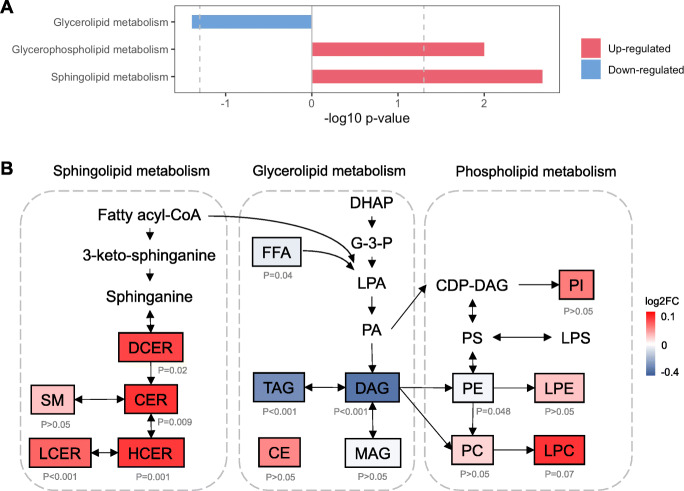
Metabolic pathways associated with intensive lactation at baseline. **A** Significantly regulated metabolic pathways (*p* < 0.05) associated with intensive lactation at baseline analyzed by Kyoto Encyclopedia of Genes and Genomes (KEGG) pathway analysis. Blue indicates downregulated pathway whereas red indicates upregulated pathway. **B** Integrated metabolic pathway of synthesis of 15 lipid classes from neutral lipids, phospholipids, and sphingolipids. Red indicates upregulation, and blue denotes downregulation with *p* value indicated

To identify metabolic pathways associated with lactation intensity at baseline, we performed Kyoto Encyclopedia of Genes and Genomes (KEGG) pathway analysis. We observed a significant downregulation of glycerolipid metabolism involving TAG/DAG biosynthesis in IBF women (*p* = 0.04) (Fig. 5A and Additional file 1: Table S5). Conversely, metabolism of sphingolipids (*p* = 0.002) and glycerophospholipids (*p* = 0.01) was shown to be significantly upregulated (Fig. 5A and Additional file 1: Table S5). These three significantly regulated pathways (glycerolipid, sphingolipid, and glycerophospholipid metabolism) are closely linked as they share common substrates such as phosphatidate and fatty acyl-CoA, suggesting a pathway switch and flux of carbon from TAG and DAG sources towards phospholipids and sphingolipids (Fig. 5B).

### Genes and master regulons related to the lactation-associated lipid species

**Fig. 6:**
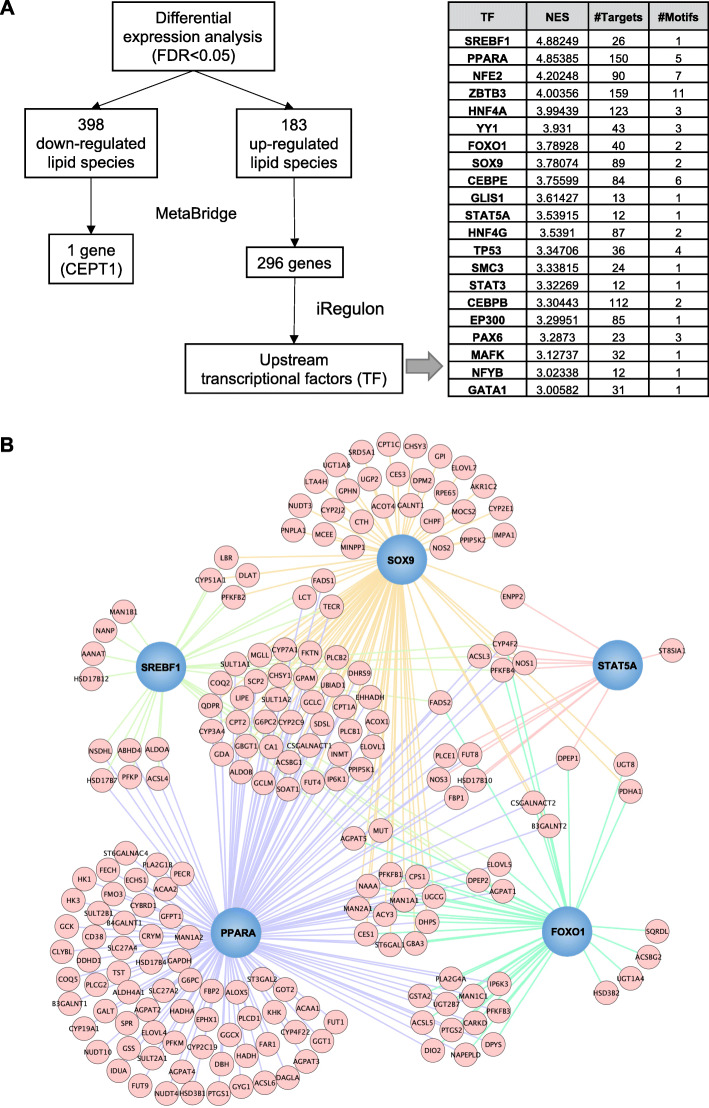
Regulatory network analysis of master regulons and genes that participate in metabolism of lipids associated with intensive lactation. **A** Flow chart of the identification of co-expressed genes and the master regulons from altered lipids by using MetaBridge and iRegulon. **B** iRegulon analysis depicting the regulatory network between the master regulons associated with lipid metabolism and their downstream target genes

Our findings suggested lactation intensity was associated with alterations in lipid metabolism. To further examine this biological change at the gene level, we used MetaBridge to cross-link genes with the differential lipids that were associated with lactation intensity [39]. iRegulon was then applied to detect master regulons from the set of genes and establish a regulatory network [40]. We found 183 upregulated lipids (mainly phospholipids and sphingolipids) were linked to 296 genes including ACSL, CERS, CPT, ELOVL, and G6PC (Additional file 1: Table S6) that participate in the biosynthesis of fatty acids, phospholipids, and sphingolipids [41–45]. By using iRegulon analysis, these 296 genes were matched to 21 master regulons (such as PPARA, SREBF1, FOXO1, SOX9, STAT5A), majority of which are involved in lipid metabolism (Fig. 6A). The cluster of targeted genes regulated by the master regulons involved in lipid metabolism during lactation was summarized in Fig. 6B. In contrast, 398 downregulated lipids (mainly TAG and DAG) were linked to only one gene CEPT1 (Fig. 6A). CEPT1 encodes choline/ethanolamine phosphotransferase 1, an enzyme that controls the formation of PC and PE from DAG [46], suggesting a close link between glycerolipids and phospholipids. No master regulon was identified due to only one gene being linked to the downregulated lipid species.

### Effects of lactation on lipid profiling at baseline in future T2D and no T2D women

**Fig. 7:**
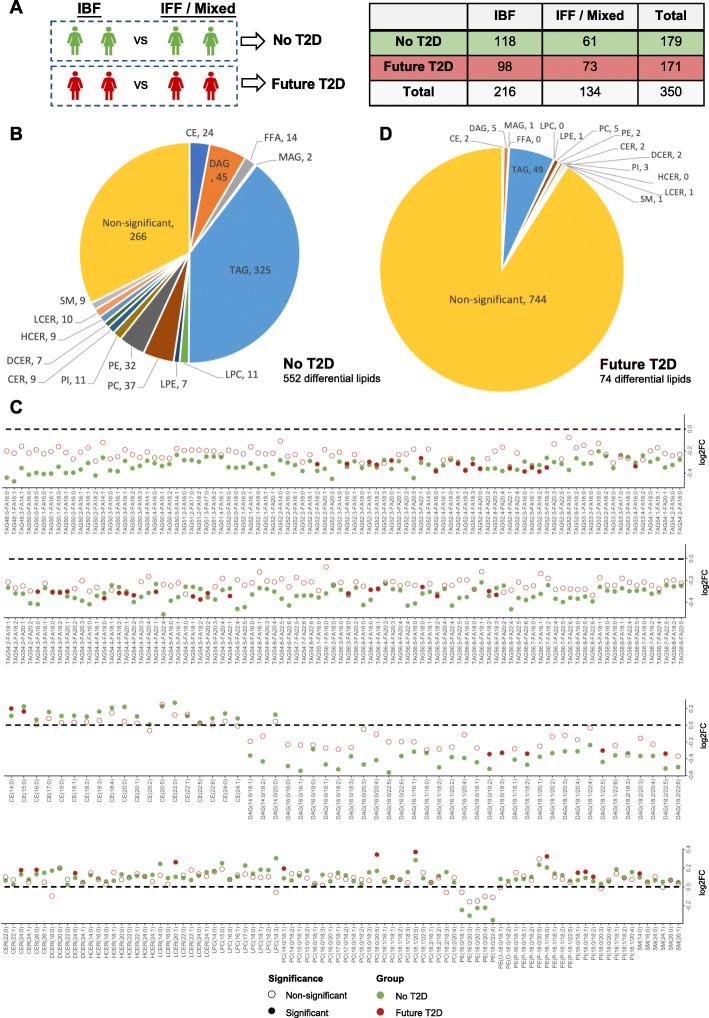
The effect of postpartum lactation on lipid profiling in future T2D and no T2D women at baseline. **A** The number of IBF and IFF/Mixed women in the future T2D subgroup and no T2D subgroup. **B** The number of significantly differentially expressed lipid species in 15 lipid classes between IBF and IFF/Mixed in the no T2D subgroup. **C** Differentially expressed lipids between IBF and IFF/Mixed in the T2D and no T2D subgroups. Log2 FC values of all differentially expressed lipids with FDR < 0.001 between IBF and IFF/Mixed in the no T2D subgroup are shown in green color. The log2 FC values of these lipid species between IBF and IFF/Mixed in future T2D subgroup are shown in red color. Solid dots represent significance, empty dots indicate non-significance. **D** The number of significantly differentially expressed lipid species in 15 lipid classes between IBF and IFF/Mixed in the future T2D subgroup

Our previous study showed that lactation intensity and duration were associated with 34-57% lower relative risk of incidence of T2D within 2 years postpartum [8]. We further stratified the subset of 350 women with recent GDM by future T2D status and examined whether intensive lactation affected lipid profiles in each subgroup. From the 350 women, 171 developed T2D and 179 did not (no T2D) during the follow-up period (up to 8 years post-baseline) (Fig. 7A). In the future T2D group, 98 (57.3%) women were categorized as IBF while 73 (42.7%) women were IFF/Mixed at baseline (Fig. 7A). In the no T2D group, 118 (65.9%) women were categorized as IBF, whereas 61 (34.1%) women were IFF/Mixed at baseline (Fig. 7A). Among the no T2D group, 552 lipid species were found to be significantly altered between IBF and IFF/Mixed women (FDR < 0.05) (Fig. 7B-C and Additional file 1: Table S7). A total of 327 differential lipid species with FDR < 0.001 were summarized and shown (Fig. 7D). Among these lipids, 185 TAGs and 35 DAGs were downregulated, whereas 19 CEs, 55 phospholipids, and 33 sphingolipids were upregulated in the IBF group compared to IFF/Mixed group (Fig. 7D). In the future T2D subgroup, we detected similar lipid changes with lower TAGs/DAGs and higher sphingolipids/phospholipids (Additional file 1: Table S8). However, the amount of significantly differentially expressed lipids in the future T2D subgroup was much less than those in women with no T2D. The same trend was also observed in the metabolomic profiling. A total of 44 phospholipids, 12 sphingolipids, 2 acylcarnitine, 6 biogenic amines, 10 amino acids, and hexose were found to be significantly different (FDR < 0.05) between IBF and IFF/Mixed groups in the no T2D women (Additional file 1: Table S9), whereas only 1 phospholipid and kynurenine were found to be significantly differentially expressed between the two groups in the future T2D women (Additional file 1: Table S10). This indicates that women who do not respond metabolically to lactation would be very likely to progress to future T2D after GDM pregnancy.

Furthermore, by comparing the clinical parameters of women who responded and did not respond metabolically to lactation, we found that the non-responders showed higher *z*-score sum of 3-h 100-g OGTT during pregnancy, and higher proportion of oral medications/insulin treatment for GDM (Additional file 1: Table S11). Moreover, at early postpartum, the non-responders exhibited higher FPG, fasting insulin, HOMA-IR, and higher proportion of glucose intolerance compared to responders.

### Prediction of future T2D in women with intensive breastfeeding

**Fig. 8:**
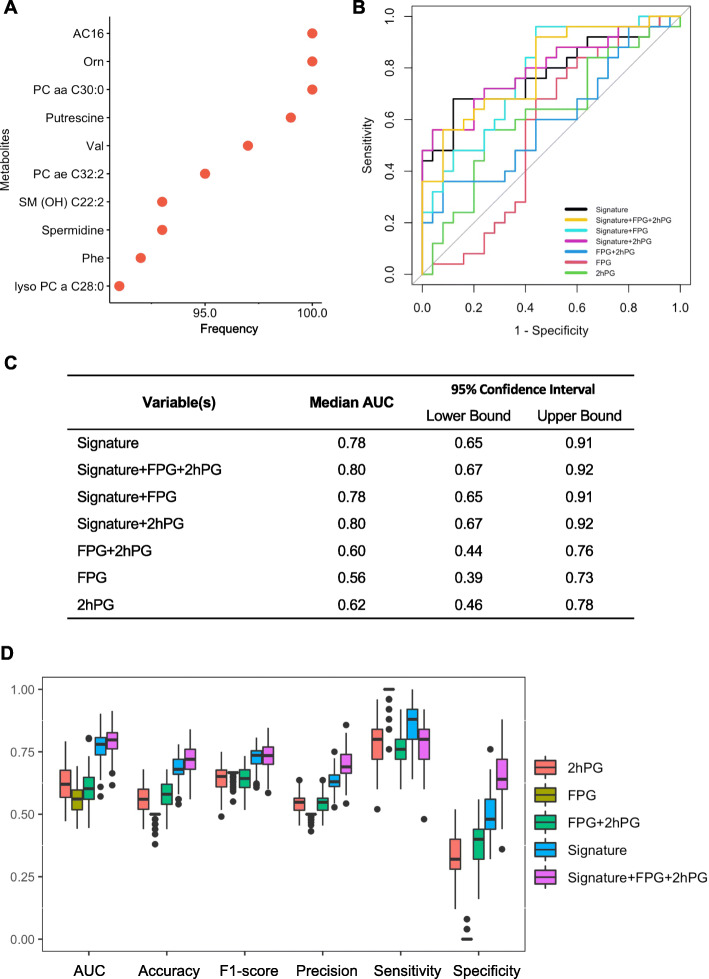
Metabolic signature to predict future T2D in IBF women at early postpartum. **A** Variable selection using random forest identified a set of 10 analytes with high predictive power. **B** ROC of the predictive models generated by 10-analyte signature and traditional clinical variables (fasting glucose and 2-h glucose) in the testing set. **C** AUC and 95% CI of the ROC curve. **D** Box plots showing the distribution of the performance of the metabolic signature and traditional clinical parameters (fasting glucose and 2-h plasma glucose) under 100 times’ repeat

To further identify who is more likely to develop future T2D even with intensive breastfeeding, we established a distinct predictive model with 10 analytes-1 acylcarnitine, 2 biogenic amines, 3 amino acids, and 4 lipids (Fig. 8A). Using the 10-analyte signature through 100 times’ validation (Additional file 1: Figure S7), we achieved a median AUC value of 0.78 (95% CI 0.65-0.91), which is far superior to FPG (median AUC 0.56, 95% CI 0.39-0.73) and 2 h-PG (median AUC 0.62, 95% CI 0.46-0.78) (Fig. 8B-D). Notably, after combining the clinical variables with the 10-analyte signature, the predictive performance was slightly improved (median AUC 0.80, 95% CI 0.67-0.92), suggesting the significance of the metabolic signature in predicting future T2D in IBF women. We also showed the prediction of future T2D by our 10-analyte signature is superior to those “non-invasive” variables and “standard measurements,” including pre-pregnancy BMI, treatment, race, family history of diabetes, total lactation duration, fasting plasma lipids, lipoproteins, and non-esterified free fatty acids (Additional file 1: Table S12). These data suggest that the metabolic changes appeared years before the real onset of T2D in women with intensive breastfeeding, which allows us to predict T2D in this specific group of women and further investigate the underlying mechanisms associated with T2D pathogenesis.

## Discussion

In the present study, we selected a subset of 350 women from a large well-characterized prospective cohort (SWIFT study) of women with recent GDM who were all systematically tested for newly onset of T2D up to 8 years post-baseline. We showed that high lactation intensity was associated with substantial effects on maternal lipid profiles in women with recent GDM. The most striking findings were downregulation of glycerol metabolism and the upregulation of sphingolipid/phospholipids metabolism appearing in participants with both normal and impaired glucose tolerance at baseline. Interestingly, these changes were not observed at follow-up or in a longitudinal analysis, indicating a convergence in the metabolome at this point. We further revealed that women who later progressed to T2D had fewer lipid changes associated with lactation intensity compared to no T2D women.

Several prenatal parameters could affect the changes in lipids observed in IBF women. In our analysis, all parameters have been considered as potential confounding factors, including age, pre-pregnancy BMI, race/ethnicity, *Z*-score sum of 3-h 100-g OGTT during pregnancy, and GDM treatment during pregnancy. We found there was no significant difference in age, race/ethnicity, *Z*-score sum of 3-h 100-g OGTT during pregnancy, and GDM treatment between IBF women and IFF/Mixed women. However, IBF women had lower average pre-pregnancy BMI levels compared to IFF/Mixed women. Therefore, in the following statistical analyses, we adjusted pre-pregnancy BMI. Since baseline measures of glucose (FPG, 2-h PG, HOMA-IR, HOMA-β, etc.) occurred after breastfeeding began, these variables might be influenced by breastfeeding in the first 6–9 weeks postpartum. Therefore, these parameters were not adjusted in the analysis.

At baseline, neutral lipids (TAGs/DAGs) were shown to be negatively associated with lactation intensity. Remarkably, 64% (328 out of 513) of measured TAGs and 83% (45 out of 54) of measured DAGs were significantly lower in IBF women. Additionally, the pathway of glycerolipid metabolism was significantly downregulated in IBF women compared to IFF/Mixed women. Decreased TAGs and DAGs in IBF women were detected in both NGT and IFG/IGT subgroups. This was consistent with previous findings by us and others [23, 47, 48]. During pregnancy, circulating TAGs surge up to 200-300% of prior pregnancy levels [49], indicating the body’s adaption for supporting fetus growth as well as preparation for lactation post-delivery. During lactation however, the maternal body utilizes TAGs and glycogen to meet the increased energy demands for mammary glands to produce milk, which is achieved mainly by promoting glycogenolysis and lipolysis [50, 51]. Thus, TAGs/DAGs are largely utilized for milk production leading to their clearance from circulation, fitting to our previous clinical biochemical data [22]. It has been previously shown that saturated fatty acids containing an even number of carbon atoms derive from endogenous sources including de novo lipogenesis [52–56].

We found that significantly downregulated TAGs/DAGs were clustered in those with backbones of an even carbon atom number, suggesting lactation may lead to suppressed endogenous lipogenesis or possibly upregulated catabolism of lipids. We particularly found that long-chain fatty acids were greatly decreased in IBF women. This could be due to the fact that long-chain fatty acids present in milk are directly transferred from plasma instead of de novo synthesized from glucose in the mammary glands [57]. The link between intensive lactation and maternal lipid metabolism was further supported by the identification of master regulons (PPARA, SREBF1, FOXO1, SOX9, STAT5A, etc.) via the integrative tools. These master regulons are involved in lipid metabolism associated with lactation. In particular, PPARA encodes peroxisome proliferator-activated receptor alpha (PPAR-α), which is known to regulate utilization and catabolism of fatty acids [58]. SREBF1 encodes the sterol regulatory element-binding transcription factor 1 (SREBP1), a transcription factor (TF) which is required for de novo biosynthesis of fatty acids, cholesterol, and triglycerides [59]. FOXO1 encodes forkhead box protein O1 (FOXO1), a TF that is involved in regulation of gluconeogenesis and glycogenolysis by insulin signaling. FOXO1 also promotes SOX9 expression and suppresses fatty acid oxidation in response to low lipid levels [60]. STAT5A encodes signal transducer and activator of transcription 5A (STAT5A), which is a TF that plays an important role in intensive breastfeeding by activating prolactin-induced transcription and regulates the expression of milk proteins during lactation [61]. Overall, this decrease of TAGs/DAGs along with the identified master regulons may contribute to the reduced risk of metabolic disorders in later life [62]. In addition to the pathways that we identified from KEGG, other lactation-associated pathways were reported previously [63]. Five metabolic pathways, including gluconeogenesis, pyruvate metabolism, the tricarboxylic acid cycle (TCA cycle), glycerolipid metabolism, and aspartate metabolism, were found to be involved in lactation. Among them, the TCA cycle was the most upregulated pathway suggesting that lactation is a process with high energy demand.

Importantly, we are the first to report that a decrease of TAGs/DAGs was accompanied by large increases in phospholipids and sphingolipids during lactation. These three lipid classes are intimately intertwined as they share common substrates such as phosphatidate and fatty acyl-CoA [64]. Therefore, it is possible that the observed downregulation of glycerolipid metabolism, in particular the suppression of lipogenesis, may shift the carbon source flux of substrates from lipogenesis (TAG/DAG formation) towards formation of phospholipids and sphingolipids. Additionally, by using MetaBridge, we identified that the downregulation of TAGs/DAGs was associated with the CEPT1 gene, which encodes choline/ethanolamine phosphotransferase 1, an enzyme that regulates the formation of phospholipids from DAG [65]. These findings suggest a close relationship between these three lipid classes. Phospholipids and sphingolipids are deeply involved in cell signaling and therefore their deficiency might lead to impaired insulin receptor signaling and insulin resistance [66–68]. We very recently showed that TAGs/DAGs were positively correlated with HOMA-IR (insulin resistance) while phospholipids/sphingolipids had a negative correlation [36]. Therefore, upregulation of sphingolipids and phospholipids accompanied by downregulation of glycerolipids may lead to reduced insulin resistance [69, 70]. Indeed, we and others reported lactating women were shown to have lower HOMA-IR than less or non-lactating women [71, 72].

In addition to lipids, we also showed significantly decreased hexose was associated with intensive lactation. This may be explained by the increased glucose uptake in mammary glands during lactation. In contrast, peripheral glucose uptake in other tissues such as liver and muscle is reduced during lactation, which has been suggested to occur in order to prioritize the glucose for milk production [50, 73]. This may lead to reduced/lower insulin demand, and therefore explain the lowered circulating insulin observed in IBF women compared to IFF/Mixed group [71, 74]. Distinct from the significant changes of lipids, hexose and amino acids were marginally changed between intensively and non-intensively lactating women, suggesting amino acid metabolism remains more stable while the TAGs/DAGs are largely utilized for milk production.

In our current study using a subset of 350 women from SWIFT (171 future diabetes vs. 179 non diabetes), we did not observe a significant difference in women who developed future diabetes between IBF and IFF/Mixed groups. This could largely be attributed to the fact that this study was a secondary analysis based on a previously selected subset, which has much higher incident T2D case numbers than the general population (50% vs. 10%). In our previous study, intensive lactation was associated with low incident diabetes rates [7, 8]. Additionally, expanding the sample size could also help reveal a difference.

In contrast to the baseline results of the 350 women examined, we did not observe significant changes in metabolites associated with intensive lactation in the subset of 303 women during cross-sectional analysis at follow-up (~ 2 years post-baseline), nor in the longitudinal analysis. We could not exclude the possibility that significant differences in metabolites between IBF and IFF/Mixed at follow-up may be observed by performing metabolomics on a larger sample set. Therefore, in future studies, we could apply omics on a larger sample size of the SWIFT study whose fasting plasma samples are available at both baseline and follow-up. Furthermore, the longer-term benefits associated with postpartum lactation may also involve other pathways including inflammatory markers or changes in lipid markers that we were unable to evaluate at follow-up in this analysis. Importantly, the changes related to concurrent lactation intensity, such as modifications at the gene level with a more long-term and persistent effect compared to metabolic changes, should be investigated.

Very recently, in an analysis of women in the SWIFT Study, we reported that higher TAGs/DAGs and lower phospholipids/sphingolipids postpartum were associated with future T2D after GDM pregnancy [36]. Interestingly, in this study, we observed a remarkable opposing lipid profile associated with IBF. It is well known that elevated TAGs/DAGs are associated with diabetes onset and other metabolic disorders [19, 20, 36]. Therefore, these current findings, from a standpoint of metabolism, support our previous findings that women with intensive lactation postpartum have reduced risk of developing diabetes compared to those who do not breast feed intensively [8].

We reported that metabolic dysregulation (including impaired glucose metabolism) was present at the early postpartum period in GDM women who would develop T2D in later years [36, 75]. In the SWIFT study, higher intensity and longer lactation duration were associated with 50% lower relative risk of T2D [8], accounting for maternal obesity and metabolic status. Other studies in women with obesity reported shortened breastfeeding duration, delayed onset of lactogenesis and lactation outcomes [76–79]. Clinically, impaired glucose metabolism and insulin sensitivity may be associated with poor lactation performance and low milk supply in women [80]. These risk factors may also influence maternal circulating lipid profiles. Thus, the association between lactation and metabolic changes could differ by future T2D status. Therefore, in our case-control subsample, we stratified according to future diabetes status. We observed that women who went on to develop T2D had far fewer lipid changes during lactation at early postpartum compared to those who did not develop T2D. This indicates that the favorable effects of intensive lactation which are inversely associated with T2D may be attributed to significant changes in lipid metabolism during lactation. However, the IBF women whose lipid profiles were not significantly altered were more likely to develop future T2D during follow-up. Further insight is required to address this directly.

Additionally, in women with intensive breastfeeding, we identified a 10-analyte signature panel to effectively predict future T2D risk, which is far superior to the predictive performance of non-invasive clinical parameters and standard measurements. This signature panel included 4 major metabolite groups, including acylcarnitine, amino acid, biogenic amine, and lipid, supporting the fact that diabetes is a metabolic disorder with dysregulation of carbohydrates, lipids, and amino acids. Three of these analytes (PC aa C30:0, SM (OH) C22:2 and spermidine) were also identified in our previous study, where we developed a 20-analyte signature to effectively predict future T2D onset after GDM pregnancy [75], suggesting the importance of these analytes in predicting future T2D. Moreover, the predictive performance of the 10-analyte signature reported here does not rely on accompanying clinical variables, suggesting the significance of the metabolic signature to predict future T2D in this specific group of women with intensive breastfeeding.

In our current study, we show a significant change in the lipid profile (lower TAGs and DAGs but higher sphingolipids and phospholipids) with higher lactation intensity, which may be the physiology underlying our previous findings on lactation’s negative association with future risk of developing metabolic syndrome and T2D in women [8, 71]. Our current findings further suggested that the favorable effects of lactation on maternal metabolic health may be exerted through changes in lipid metabolism. Longitudinal studies with both lipidomics and metabolomics performed on a larger sample size and in an independent cohort would further illuminate the persistent effects of lactation on the metabolic pathways related to diabetogenesis. In terms of the effects of prolonged storage time on the human plasma metabolome, a study reported only 2% tested plasma metabolites were found to be altered in the first 7 years of storage, and up to 26% of metabolites were changed upon longer storage periods up to 16 years [81]. Therefore, the changes in the metabolites of the plasma over the storage time should also be considered. Regardless, our study has advanced our understanding of lactation-associated biochemical pathways and their relationship with diabetes risk in women. These may help to identify specific molecular targets to improve women’s health.

## Conclusions

This study showed that intensive lactation significantly alters the circulating lipid profile at early postpartum and that women who do not respond metabolically to lactation are more likely to develop T2D. We also identified a metabolic signature that accurately predicts future onset of T2D in IBF women. Our findings provide novel insight into how lactation influences maternal metabolism and its link to future diabetes onset.

## Supplementary Information

**Additional file 1: Supplementary Figure S1-S7 and Table S1-S13. Figure S1.** Quality control of the final metabolomics dataset at baseline and follow-up. **Figure S2.** Longitudinal analysis of metabolites between IBF and IFF/Mixed women from baseline to follow-up. **Figure S3.** Quality control of the final lipidomics dataset at baseline. **Figure S4.** Effects of postpartum lactation intensity on lipid profiling at baseline in IFG/IGT and NGT women. **Figure S5.** Effects of different lactation intensity on lipid profiling at early postpartum. **Figure S6.** Metabolites associated with extreme lactation intensity at baseline. **Figure S7.** Generation of the predictive models. **Table S1.** Differential analytes between IBF and IFF/Mixed women at baseline. **Table S2.** Differential analytes between IBF and IFF/Mixed women at follow-up. **Table S3.** Differential lipid species between IBF and IFF/Mixed women at baseline. **Table S4.** Relationship between lactation intensity and fatty acid composition in lipids. **Table S5.** Pathways associated with lactation intensity at baseline. **Table S6.** Potential genes associated with differentially expressed lipid species at baseline. **Table S7.** Differential lipid species between IBF and IFF/Mixed women in the no T2D subgroup. **Table S8.** Differential lipid species between IBF and IFF/Mixed women in the future T2D subgroup. **Table S9.** Differential analytes between IBF and IFF/Mixed women in the no T2D subgroup. **Table S10.** Differential analytes between IBF and IFF/Mixed women in the future T2D subgroup. **Table S11.** Baseline clinical characteristics of responders and non-responders in the present study. **Table S12.** Predictive performance of 10-analyte signature, non-invasive variables and standard measurements. **Table S13.** Differential analytes between T2D and no T2D women in the IBF group.

## Data Availability

The datasets used and/or analyzed during the current study are available from the corresponding authors upon reasonable request.

## Abbreviations

2 h-PG: 2 h plasma glucose
AA: Amino acid
AC: Acylcarnitine
AUC: Area under the curve
BA: Biogenic amine
CARDIA: Coronary Artery Risk Development Study in Young Adults
CE: Cholesterol ester
CER: Ceramide
DCER: Dihydroceramide
DAG: Diacylglycerol
FDR: False discovery rate
FFA: Free fatty acid
FPG: Fasting plasma glucose
GDM: Gestational diabetes mellitus
GWAS: Genome-wide association study
HCER: Hexosylceramide
HDL: High-density lipoprotein
HR: Hazard ratio
IBF: Intensive breastfeeding
IFF/Mixed: Intensive formula feeding or mixed feeding
IFG: Impaired fasting glucose
IGT: Impaired glucose tolerance
KEGG: Kyoto Encyclopedia of Genes and Genomes
KPNC: Kaiser Permanente Northern California
LCER: Lactosylceramide
LDL: Low-density lipoprotein
LIR: Lactation intensity/duration ratio
LOD: Limit of detection
LPC: Lysophosphatidylcholine
LPE: Lysophosphatidylethanolamine
MAG: Monoacylglycerol
NGT: Normal glucose tolerance
OGTT: Oral glucose tolerance test
PC: Phosphatidylcholine
PCA: Principal component analysis
PE: Phosphatidylethanolamine
PI: Phosphatidylinositol
PLS-DA: Partial least squares-discriminant analysis
SM: Sphingomyelin
SNP: Single-nucleotide polymorphism
SWIFT: Study of Women, Infant Feeding, and Type 2 Diabetes after GDM Pregnancy
T2D: Type 2 diabetes mellitus
TAG: Triacylglycerol
TF: Transcription factor
VIP: Variable importance
