# Supplementary material for: Intensive lactation among women with recent gestational diabetes significantly alters the early postpartum circulating lipid profile: the SWIFT study
Source: BMC Med. 2021 Oct 8;19:241. doi: 10.1186/s12916-021-02095-1 (PMC8499506; doi:10.1186/s12916-021-02095-1)
Supplement: Supplementary file 1 — Additional file 1: Supplementary Figure S1-S7 and Table S1-S13. Figure S1. Quality control of the final metabolomics dataset at baseline and follow-up. Figure S2. Longitudinal analysis of metabolites between IBF and IFF/Mixed women from baseline to follow-up. Figure S3. Quality control of the final lipidomics dataset at baseline. Figure S4. Effects of postpartum lactation intensity on lipid profiling at baseline in IFG/IGT and NGT women. Figure S5. Effects of different lactation intensity on lipid profiling at early postpartum. Figure S6. Metabolites associated with extreme lactation intensity at baseline. Figure S7. Generation of the predictive models. Table S1. Differential analytes between IBF and IFF/Mixed women at baseline. Table S2. Differential analytes between IBF and IFF/Mixed women at follow-up. Table S3. Differential lipid species between IBF and IFF/Mixed women at baseline. Table S4. Relationship between lactation intensity and fatty acid composition in lipids. Table S5. Pathways associated with lactation intensity at baseline. Table S6. Potential genes associated with differentially expressed lipid species at baseline. Table S7. Differential lipid species between IBF and IFF/Mixed women in the no T2D subgroup. Table S8. Differential lipid species between IBF and IFF/Mixed women in the future T2D subgroup. Table S9. Differential analytes between IBF and IFF/Mixed women in the no T2D subgroup. Table S10. Differential analytes between IBF and IFF/Mixed women in the future T2D subgroup. Table S11. Baseline clinical characteristics of responders and non-responders in the present study. Table S12. Predictive performance of 10-analyte signature, non-invasive variables and standard measurements. Table S13. Differential analytes between T2D and no T2D women in the IBF group. [file 12916_2021_2095_MOESM1_ESM.pdf]

Intensive lactation among women with recent gestational diabetes significantly alters the early postpartum circulating lipid profile: the SWIFT study - Supplementary Material

Contents

Figure S1. Quality control of the final metabolomics dataset at baseline and follow-up……….….3

Figure S2. Longitudinal analysis of metabolites between IBF and IFF/Mixed women from baseline to follow-up…………………………………………………………………………..……5

Figure S3. Quality control of the final lipidomics dataset at baseline……………………….……..8

Figure S4. Effects of postpartum lactation intensity on lipid profiling at baseline in IFG/IGT and NGT women………………………………………………………………………..….10

Figure S5. Effects of different lactation intensity on lipid profiling at early postpartum…………12

Figure S6. Metabolites associated with extreme lactation intensity at baseline…………………..14

Figure S7. Generation of the predictive models…………………………………………………..16

Table S1. Differential analytes between IBF and IFF/Mixed women at baseline………………..17

Table S2. Differential analytes between IBF and IFF/Mixed women at follow-up………………19

Table S3. Differential lipid species between IBF and IFF/Mixed women at baseline……………20

Table S4. Relationship between lactation intensity and fatty acid composition in lipids…………33

Table S5. Pathways associated with lactation intensity at baseline………………………………34

Table S6. Potential genes associated with differentially expressed lipid species at baseline……..35

Table S7. Differential lipid species between IBF and IFF/Mixed women in the no T2D subgroup……………………………………………………………………………………42

Table S8. Differential lipid species between IBF and IFF/Mixed women in the future T2D subgroup………………………………………………………………………...…………..55

Table S9. Differential analytes between IBF and IFF/Mixed women in the no T2D subgroup….57

Table S10. Differential analytes between IBF and IFF/Mixed women in the future T2D subgroup…………………………………………………………………………………….59

Table S11. Baseline clinical characteristics of responders and non-responders in the present study……………………………………………………………………………………..….60

Table S12. Predictive performance of 10-analyte signature, non-invasive variables and standard measurements……………………………………………………………………...…..62

Table S13. Differential analytes between T2D and no T2D women in the IBF group……………63

Supplementary Figures

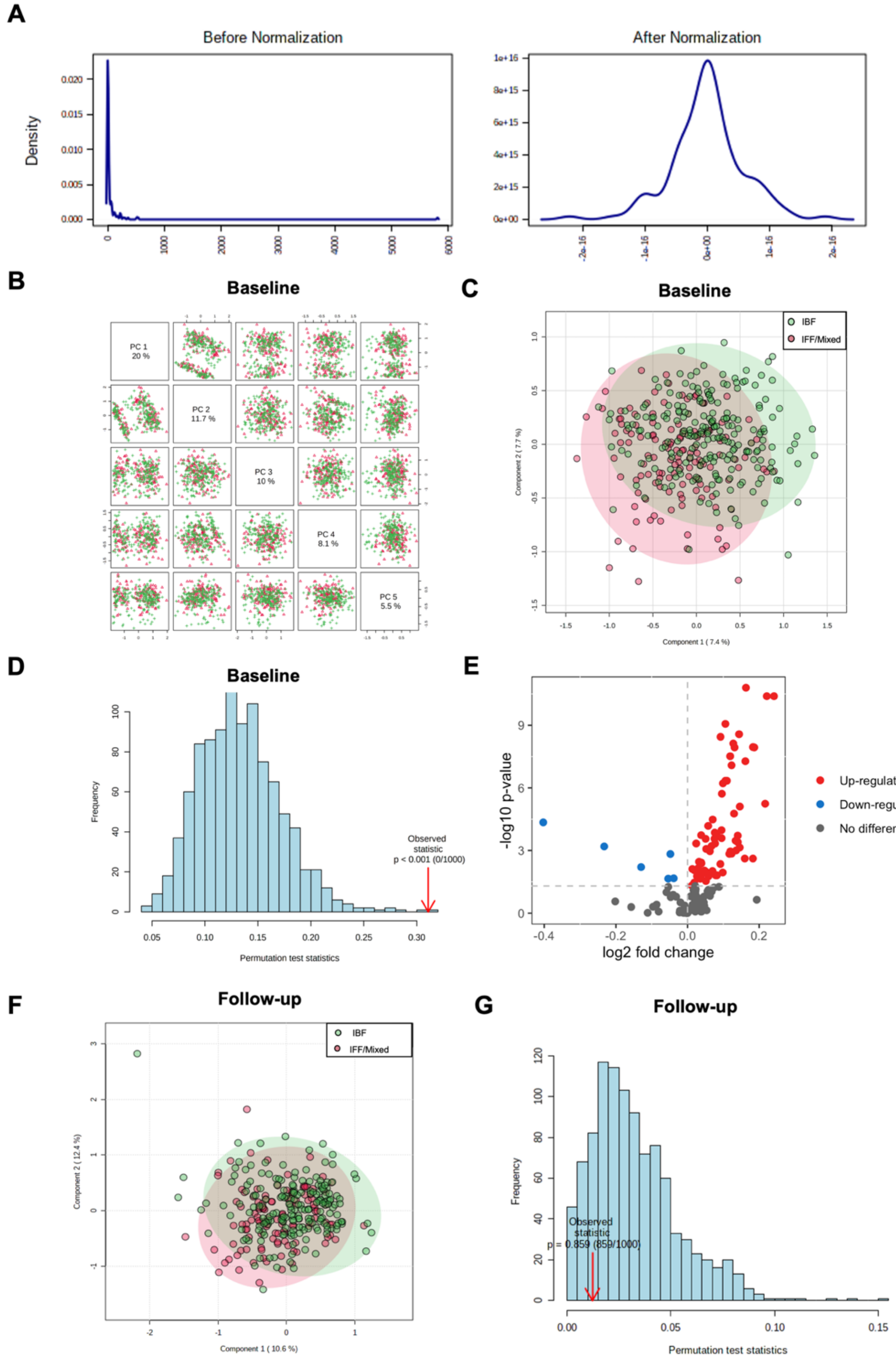

Figure S1. Quality control of the final metabolomics dataset at baseline and follow-up. (**A**) The distribution plot of the dataset before and after data normalization and transformation. (**B**) The contribution of different principal components in the PCA analysis. (**C**) PLS-DA score plot indicated the class separation between IBF group and IFF/Mixed group at baseline. (**D**) The empirical Bayes estimation with 1000 random permutations of PLS-DA analysis at baseline. Significance was indicated by p-value<0.05. (**E**) Volcano plot showed log2 fold change (log2FC) against -log10 FDR of 141 metabolites measured at baseline in IBF group compared with IFF/Mixed group. Red points indicate significantly up-regulated metabolites, blue denotes significantly down-regulated, and grey indicates no significant change. Significance was indicated by FDR<0.05. (**F**) PLS-DA score plot indicating the class separation between IBF group and IFF/Mixed group at follow-up. (**G**) The empirical Bayes estimation with 1000 random permutations of PLS-DA analysis at follow-up. Significance was indicated by p-value<0.05.

A

	IBF	IFF/Mixed	Total
**No T2D**	101 (53.7%)	51 (44.3%)	152
**Short-term T2D***	60 (31.9%)	42 (36.5%)	102
**Long-term T2D***	27 (14.4%)	22 (19.1%)	49
**Total**	188	115	303

*Short-term T2D: <32 months, Long-term T2D:>=32 months

B

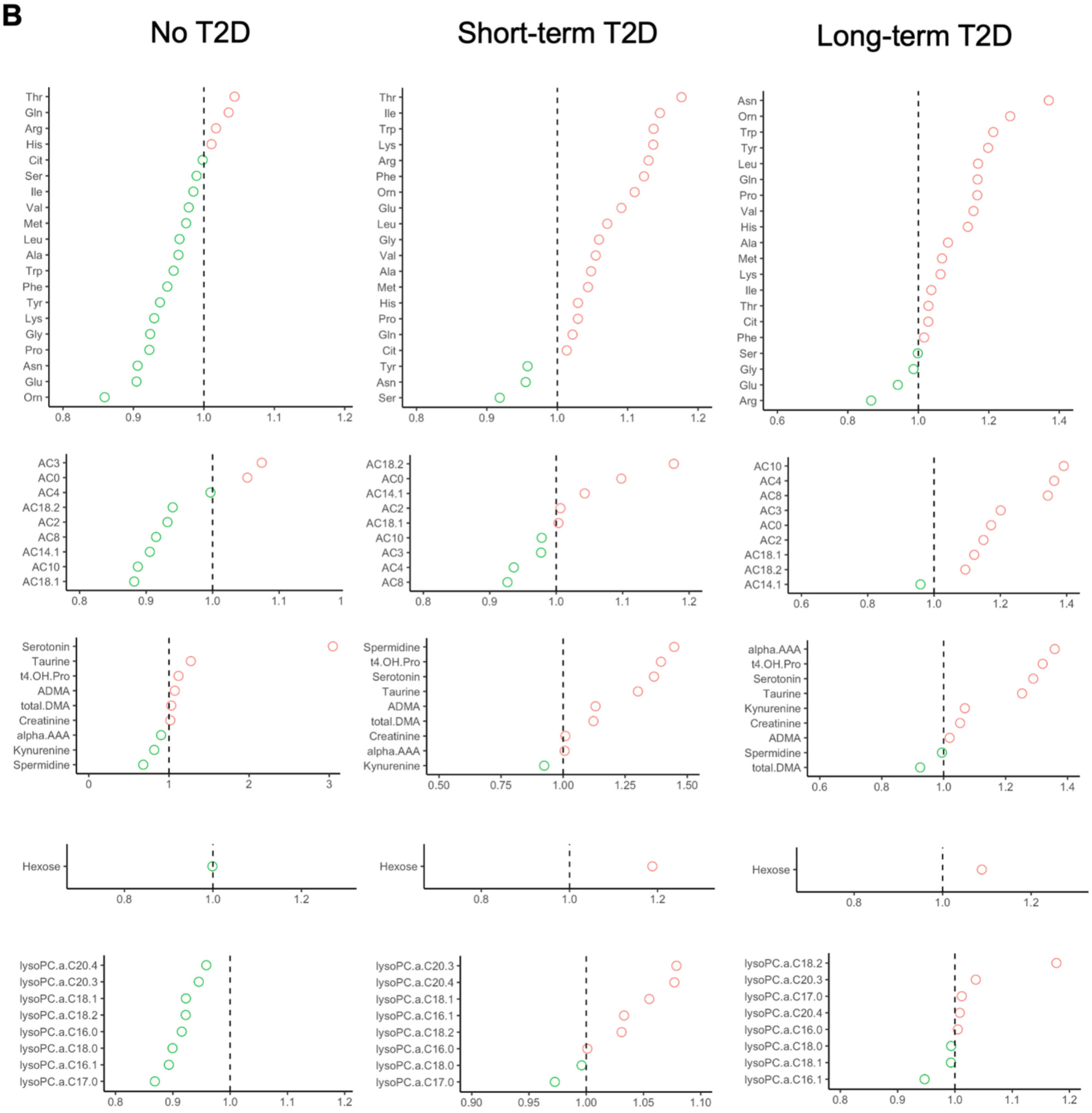

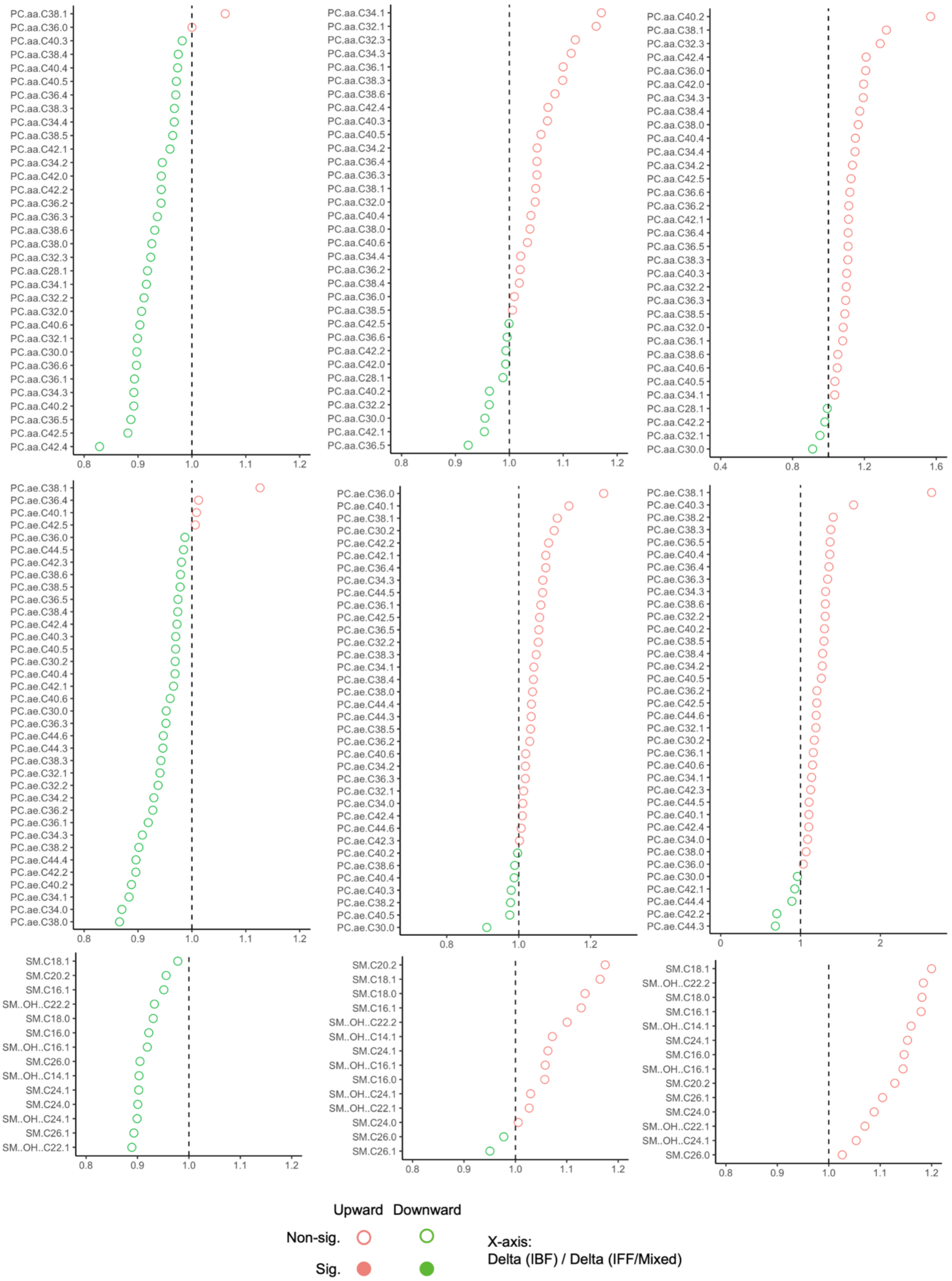
PC.aa.C38.1
PC.aa.C36.0
PC.aa.C40.3
PC.aa.C38.4
PC.aa.C40.4
PC.aa.C40.5
PC.aa.C36.4
PC.aa.C38.3
PC.aa.C34.4
PC.aa.C38.5
PC.aa.C42.1
PC.aa.C34.2
PC.aa.C42.0
PC.aa.C42.2
PC.aa.C36.2
PC.aa.C36.3
PC.aa.C38.6
PC.aa.C38.0
PC.aa.C32.3
PC.aa.C28.1
PC.aa.C34.1
PC.aa.C32.2
PC.aa.C32.0
PC.aa.C40.6
PC.aa.C32.1
PC.aa.C30.0
PC.aa.C36.6
PC.aa.C36.1
PC.aa.C34.3
PC.aa.C40.2
PC.aa.C36.5
PC.aa.C42.5
PC.aa.C42.4
0.8 0.9 1.0 1.1 1.2
PC.aa.C34.1
PC.aa.C32.1
PC.aa.C32.3
PC.aa.C34.3
PC.aa.C36.1
PC.aa.C38.3
PC.aa.C38.6
PC.aa.C42.4
PC.aa.C40.3
PC.aa.C40.5
PC.aa.C34.2
PC.aa.C36.4
PC.aa.C36.3
PC.aa.C38.1
PC.aa.C32.0
PC.aa.C40.4
PC.aa.C38.0
PC.aa.C40.6
PC.aa.C34.4
PC.aa.C36.2
PC.aa.C38.4
PC.aa.C36.0
PC.aa.C38.5
PC.aa.C42.5
PC.aa.C36.6
PC.aa.C42.2
PC.aa.C42.0
PC.aa.C28.1
PC.aa.C40.2
PC.aa.C32.2
PC.aa.C30.0
PC.aa.C42.1
PC.aa.C36.5
0.8 0.9 1.0 1.1 1.2
PC.aa.C40.2
PC.aa.C38.1
PC.aa.C32.3
PC.aa.C42.4
PC.aa.C36.0
PC.aa.C42.0
PC.aa.C34.3
PC.aa.C38.4
PC.aa.C38.0
PC.aa.C40.4
PC.aa.C34.4
PC.aa.C34.2
PC.aa.C42.5
PC.aa.C36.6
PC.aa.C36.2
PC.aa.C42.1
PC.aa.C36.4
PC.aa.C36.5
PC.aa.C38.3
PC.aa.C40.3
PC.aa.C32.2
PC.aa.C36.3
PC.aa.C38.5
PC.aa.C32.0
PC.aa.C36.1
PC.aa.C38.6
PC.aa.C40.6
PC.aa.C40.5
PC.aa.C34.1
PC.aa.C28.1
PC.aa.C42.2
PC.aa.C32.1
PC.aa.C30.0
0.4 0.8 1.2 1.6
PC.ae.C38.1
PC.ae.C36.4
PC.ae.C40.1
PC.ae.C42.5
PC.ae.C36.0
PC.ae.C44.5
PC.ae.C42.3
PC.ae.C38.6
PC.ae.C38.5
PC.ae.C36.5
PC.ae.C38.4
PC.ae.C42.4
PC.ae.C40.3
PC.ae.C40.5
PC.ae.C30.2
PC.ae.C40.4
PC.ae.C42.1
PC.ae.C40.6
PC.ae.C30.0
PC.ae.C36.3
PC.ae.C44.6
PC.ae.C44.3
PC.ae.C38.3
PC.ae.C32.1
PC.ae.C32.2
PC.ae.C34.2
PC.ae.C36.2
PC.ae.C36.1
PC.ae.C34.3
PC.ae.C38.2
PC.ae.C44.4
PC.ae.C42.2
PC.ae.C40.2
PC.ae.C34.1
PC.ae.C34.0
PC.ae.C38.0
0.8 0.9 1.0 1.1 1.2
PC.ae.C36.0
PC.ae.C40.1
PC.ae.C38.1
PC.ae.C30.2
PC.ae.C42.2
PC.ae.C42.1
PC.ae.C36.4
PC.ae.C34.3
PC.ae.C44.5
PC.ae.C36.1
PC.ae.C42.5
PC.ae.C36.5
PC.ae.C32.2
PC.ae.C38.3
PC.ae.C34.1
PC.ae.C38.4
PC.ae.C38.0
PC.ae.C44.4
PC.ae.C44.3
PC.ae.C38.5
PC.ae.C36.2
PC.ae.C40.6
PC.ae.C34.2
PC.ae.C36.3
PC.ae.C32.1
PC.ae.C34.0
PC.ae.C42.4
PC.ae.C44.6
PC.ae.C42.3
PC.ae.C40.2
PC.ae.C38.6
PC.ae.C40.4
PC.ae.C40.3
PC.ae.C38.2
PC.ae.C40.5
PC.ae.C30.0
0.8 1.0 1.2
PC.ae.C38.1
PC.ae.C40.3
PC.ae.C38.2
PC.ae.C38.3
PC.ae.C36.5
PC.ae.C40.4
PC.ae.C36.4
PC.ae.C36.3
PC.ae.C34.3
PC.ae.C38.6
PC.ae.C32.2
PC.ae.C40.2
PC.ae.C38.5
PC.ae.C38.4
PC.ae.C34.2
PC.ae.C40.5
PC.ae.C36.2
PC.ae.C42.5
PC.ae.C44.6
PC.ae.C32.1
PC.ae.C30.2
PC.ae.C36.1
PC.ae.C40.6
PC.ae.C34.1
PC.ae.C42.3
PC.ae.C44.5
PC.ae.C40.1
PC.ae.C42.4
PC.ae.C34.0
PC.ae.C38.0
PC.ae.C36.0
PC.ae.C30.0
PC.ae.C42.1
PC.ae.C44.4
PC.ae.C42.2
PC.ae.C44.3
0 1 2
SM.C18.1
SM.C20.2
SM.C16.1
SM..OH..C22.2
SM.C18.0
SM.C16.0
SM..OH..C16.1
SM.C26.0
SM..OH..C14.1
SM.C24.1
SM.C24.0
SM..OH..C24.1
SM.C26.1
SM..OH..C22.1
0.8 0.9 1.0 1.1 1.2
SM.C20.2
SM.C18.1
SM.C18.0
SM.C16.1
SM..OH..C22.2
SM..OH..C14.1
SM.C24.1
SM..OH..C16.1
SM.C16.0
SM..OH..C24.1
SM..OH..C22.1
SM.C24.0
SM.C26.0
SM.C26.1
0.8 0.9 1.0 1.1 1.2
SM.C18.1
SM..OH..C22.2
SM.C18.0
SM.C16.1
SM..OH..C14.1
SM.C24.1
SM.C16.0
SM..OH..C16.1
SM.C20.2
SM.C26.1
SM.C24.0
SM..OH..C22.1
SM..OH..C24.1
SM.C26.0
0.8 0.9 1.0 1.1 1.2
Upward Downward
Non-sig.
Sig.
X-axis:
Delta (IBF) / Delta (IFF/Mixed)

Figure S2. Longitudinal analysis of metabolites between IBF and IFF/Mixed women from baseline to follow-up. (A) The number of IBF and IFF/Mixed women in the no T2D, short-term T2D and long-term T2D subgroups. **(B)**The bubble plot represents the comparison of dynamic change of each metabolite between IBF and IFF/Mixed groups in no T2D, short-term T2D and long-term T2D, calculated by dividing the delta value (follow-up/baseline) of IBF group by the delta value of IFF/Mixed group. Red indicates dynamic change in IBF group greater than IFF/Mixed group, whereas green indicates dynamic change in IFF/Mixed group greater than IBF group. Solid dots represent significance, empty dots indicate no significance.

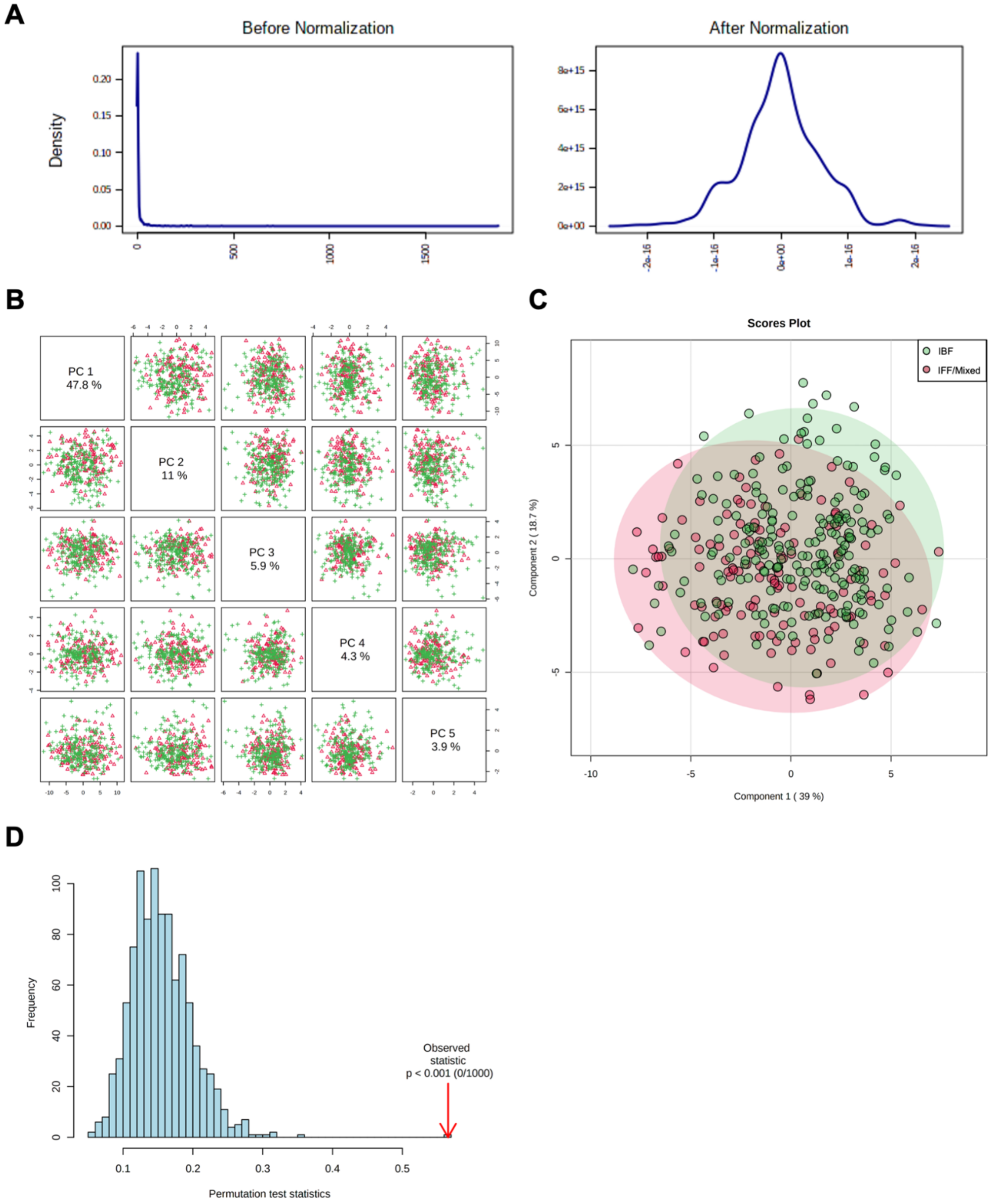

Figure S3. Quality control of the final lipidomics dataset at baseline. (**A**) The distribution plot of the dataset before and after data normalization and transformation. (**B**) The contribution of different principal components in the PCA analysis. (**C**) PLS-DA score plot indicated the class separation between IBF group and IFF/Mixed group at baseline. (**D**) The empirical Bayes

estimation with 1000 random permutations of PLS-DA analysis at baseline. Significance was indicated by p-value<0.05.

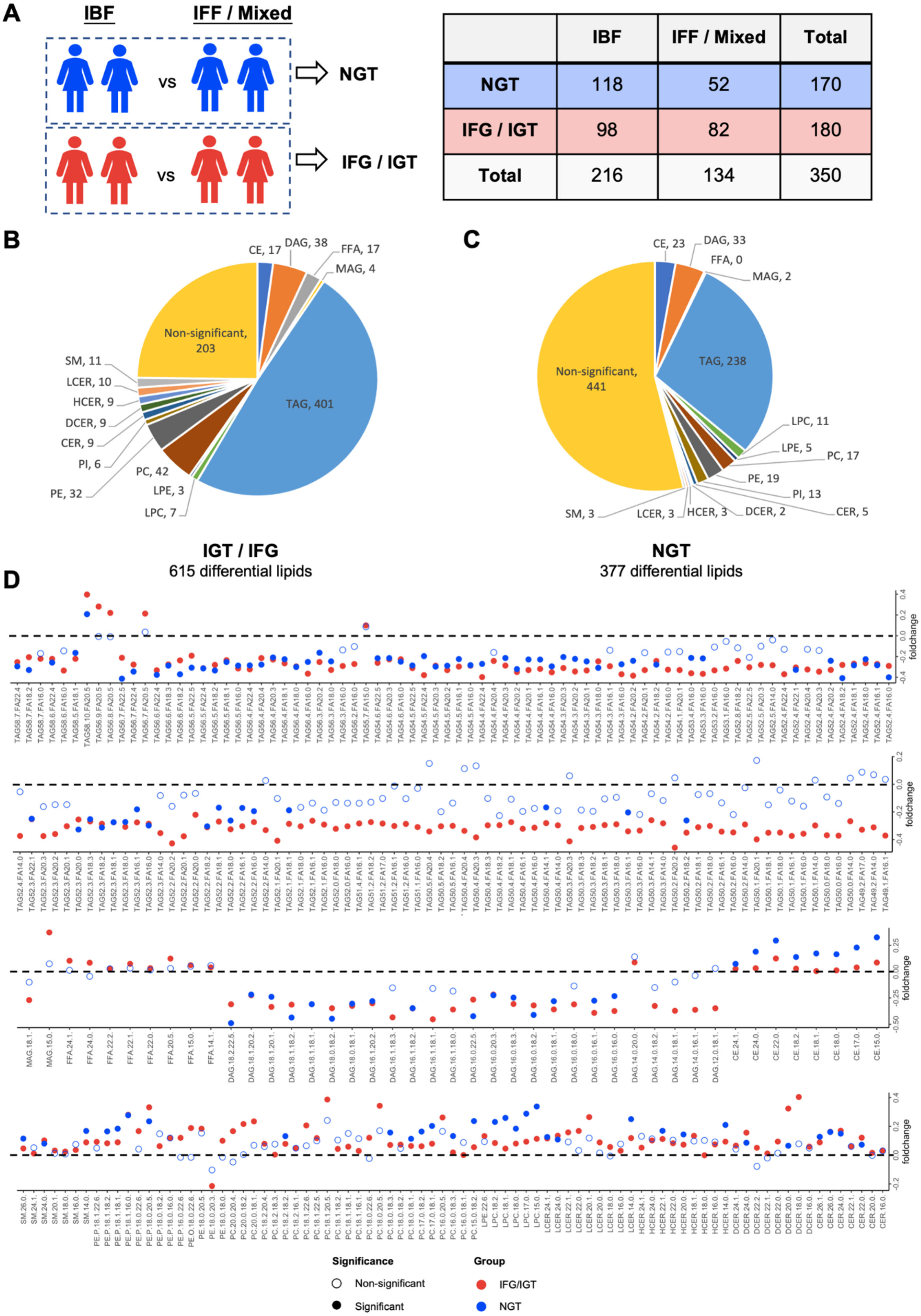

	IBF	IFF / Mixed	Total
NGT	118	52	170
IFG / IGT	98	82	180
Total	216	134	350

Figure S4. Effects of postpartum lactation intensity on lipid profiling at baseline in IFG/IGT and NGT women. (**A**) The number of IBF and IFF/Mixed women in the NGT subgroup and IFG/IGT subgroup. (**B** and **C**) The number of significantly differentially expressed lipid species in 15 lipid classes between IBF and IFF/Mixed in the NGT subgroup and IFG/IGT subgroup. (**D**) Log2 FC values of all differentially expressed lipids with FDR<0.001 between IBF and IFF/Mixed in IFG/IGT subgroup were shown in red. The log2 FC values of these lipid species between IBF and IFF/Mixed in NGT subgroup were shown in blue. Solid dots represent significance, empty dots indicate non-significance.

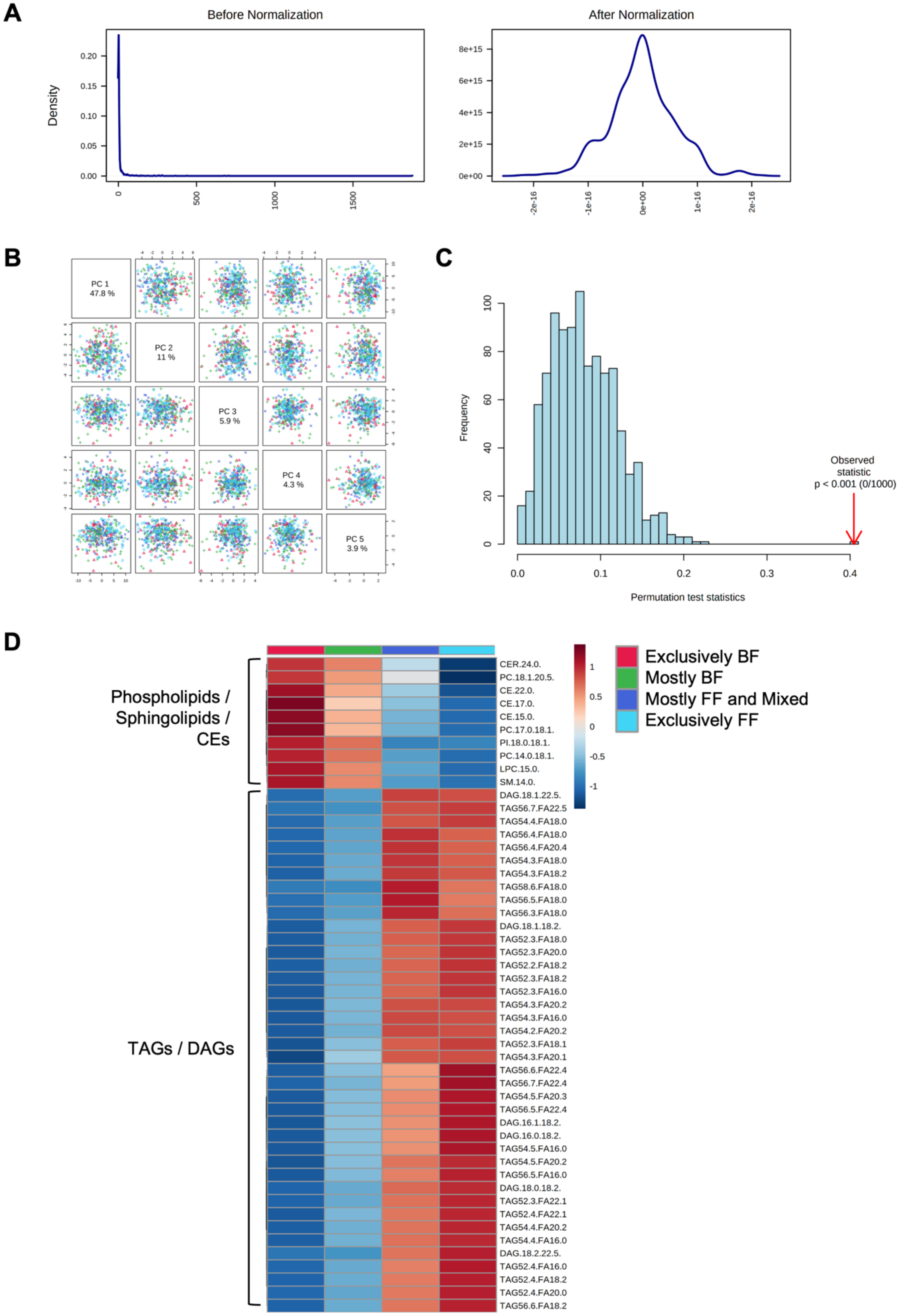
A
Before Normalization
After Normalization
Density
0.20
0.15
0.10
0.05
0.00
0
500
1000
1500
8e+15
6e+15
4e+15
2e+15
0e+00
-2e-16
-1e-16
0e+00
1e-16
2e-16
B
PC 1
47.8 %
PC 2
11 %
PC 3
5.9 %
PC 4
4.3 %
PC 5
3.9 %
C
Frequency
Observed
statistic
p < 0.001 (0/1000)
Permutation test statistics
0.0
0.1
0.2
0.3
0.4
D
Phospholipids /
Sphingolipids /
CEs
TAGs / DAGs
Exclusively BF
Mostly BF
Mostly FF and Mixed
Exclusively FF
CER.24.0.
PC.18.1.20.5.
CE.22.0.
CE.17.0.
CE.15.0.
PC.17.0.18.1.
PI.18.0.18.1.
PC.14.0.18.1.
LPC.15.0.
SM.14.0.
DAG.18.1.22.5.
TAG56.7.FA22.5
TAG54.4.FA18.0
TAG56.4.FA18.0
TAG56.4.FA20.4
TAG54.3.FA18.0
TAG54.3.FA18.2
TAG58.6.FA18.0
TAG56.5.FA18.0
TAG56.3.FA18.0
DAG.18.1.18.2.
TAG52.3.FA18.0
TAG52.3.FA20.0
TAG52.2.FA18.2
TAG52.3.FA18.2
TAG52.3.FA16.0
TAG54.3.FA20.2
TAG54.3.FA16.0
TAG54.2.FA20.2
TAG52.3.FA18.1
TAG54.3.FA20.1
TAG56.6.FA22.4
TAG56.7.FA22.4
TAG54.5.FA20.3
TAG56.5.FA22.4
DAG.16.1.18.2.
DAG.16.0.18.2.
TAG54.5.FA16.0
TAG54.5.FA20.2
TAG56.5.FA16.0
DAG.18.0.18.2.
TAG52.3.FA22.1
TAG52.4.FA22.1
TAG54.4.FA20.2
TAG54.4.FA16.0
DAG.18.2.22.5.
TAG52.4.FA16.0
TAG52.4.FA18.2
TAG52.4.FA20.0
TAG56.6.FA18.2
1
0.5
0
-0.5
-1

Figure S5. Effects of different lactation intensity on lipid profiling at early postpartum. (**A**) The distribution plot of the dataset before and after data normalization and transformation. (**B**) The contribution of different principal components in the PCA analysis. (**C**) The empirical Bayes estimation with 1000 random permutations of PLS-DA analysis at baseline. Significance was indicated by p-value<0.05. (**D**) Women were classified into four lactation intensity groups based on the infant feeding data collected from delivery to 6-9 weeks postpartum. These four groups include: 1) exclusively breastfeeding (no formula or other feeds); 2) mostly breastfeeding (≤6 oz of formula per 24 hours); 3) mostly formula (>17 oz per 24 hours) and mixed feeding (7-17 oz of formula per 24 hours or change to increase formula); 4) exclusively formula feeding (formula only; no breastfeeding or breastfeeding <3 weeks since birth). A heatmap of the top 50 differentially expressed lipid species between the four groups (exclusively BF, mostly BF, mostly FF/Mixed, exclusively FF) at study baseline.

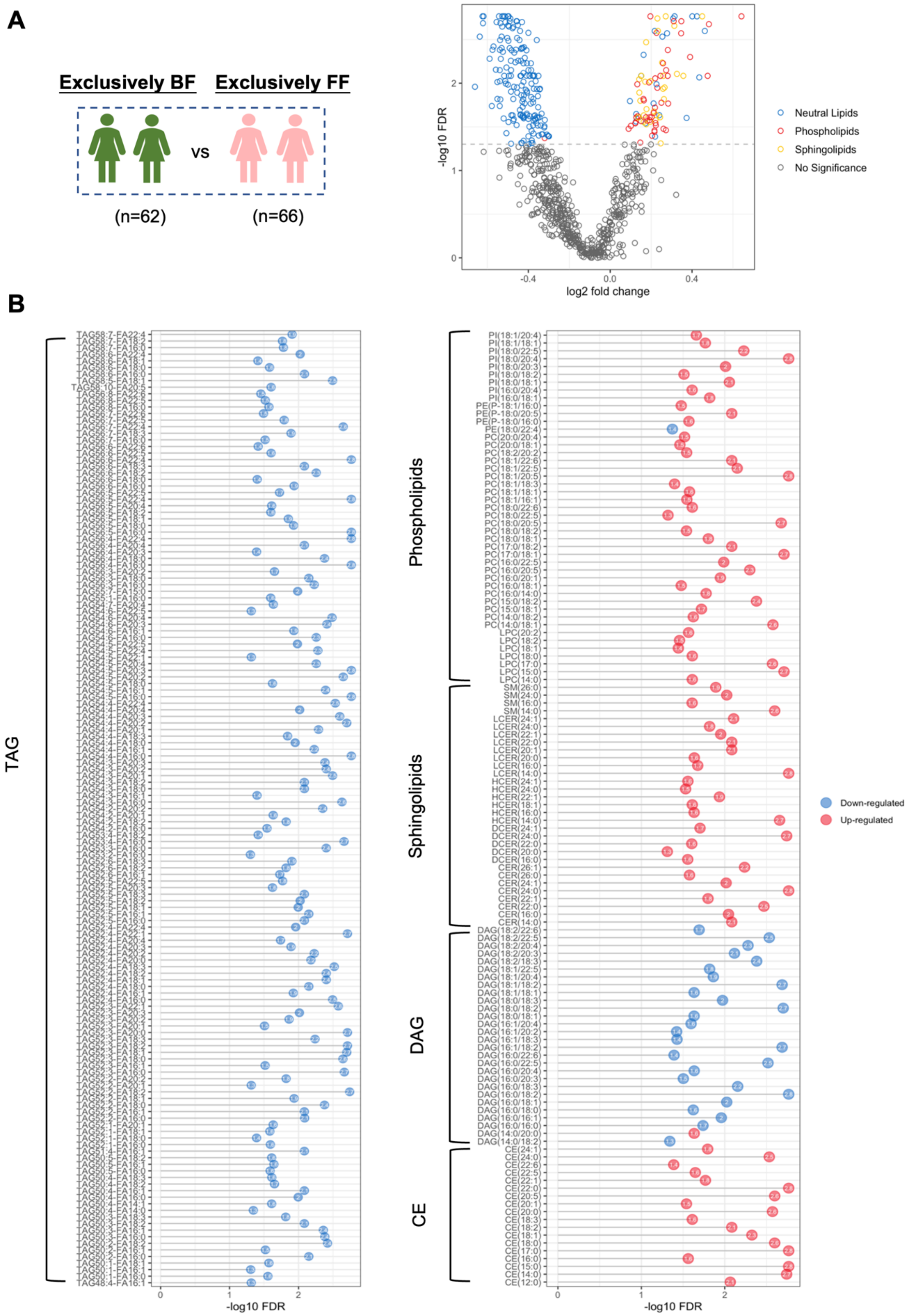
A
Exclusively BF
Exclusively FF
vs
(n=62)
(n=66)
-log10 FDR
log2 fold change
Neutral Lipids
Phospholipids
Sphingolipids
No Significance
B
TAG
Phospholipids
Sphingolipids
DAG
CE
-log10 FDR
-log10 FDR
Down-regulated
Up-regulated

Figure S6. Metabolites associated with extreme lactation intensity at baseline. (**A**) Comparison of exclusively breastfeeding women (n=62) with exclusively formula feeding women (n=66). Volcano plot showing log2 FC against -log10 FDR of 818 lipid species measured at baseline in the exclusively BF group compared to the exclusively FF group. Blue points indicate significantly differentially expressed neutral lipids, red denotes phospholipids and yellow indicate sphingolipids. Grey points indicate no significant change. Significance was indicated by FDR<0.05. (**B**) Bubble plot showing the significantly differentially expressed lipid species associated with extreme lactation intensity. Red points indicate up-regulation and blue points denote down-regulation.

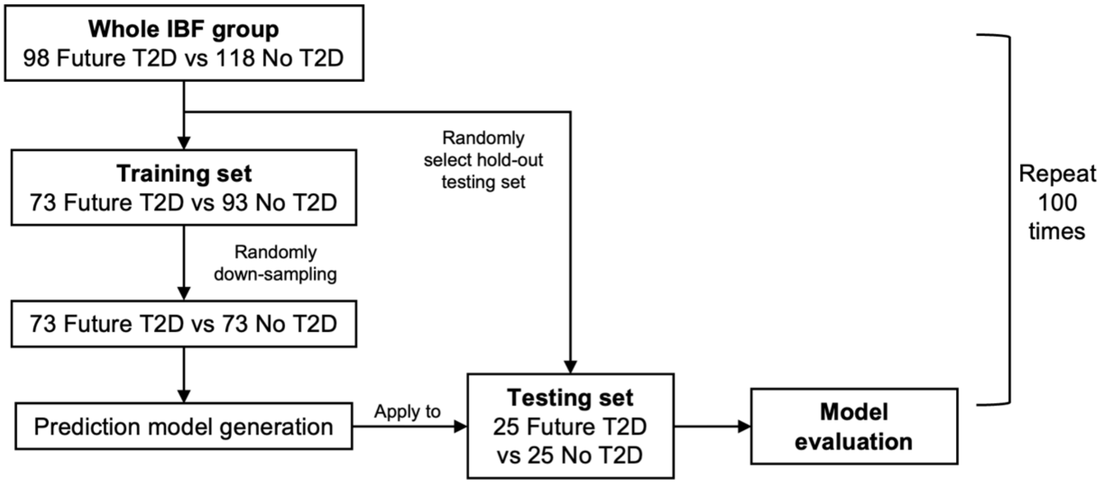

Figure S7. Generation of the predictive models. Workflow of generation of predictive model and model evaluation.

Table S1. Differential analytes between IBF and IFF/Mixed women at baseline.

Metabolites	IFF/Mixed_Mean	IFF/Mixed_SD	IBF_Mean	IBF_SD	P-value	FDR
SM (OH) C24:1	1.33	0.39	1.48	0.39	1.13E-13	1.59E-11
lysoPC a C17:0	0.82	0.30	0.96	0.37	5.73E-13	4.04E-11
Kynurenine	2.58	0.80	3.21	2.30	8.60E-13	4.04E-11
PC ae C36:1	6.40	1.66	6.89	1.69	2.43E-11	8.58E-10
PC aa C28:1	2.17	0.56	2.40	0.67	9.51E-11	2.68E-09
PC ae C34:1	7.80	1.74	8.35	2.08	1.52E-10	3.58E-09
SM (OH) C22:1	15.88	3.96	17.35	4.33	3.71E-10	7.46E-09
lysoPC a C18:1	9.64	3.93	10.72	3.64	6.32E-10	1.11E-08
lysoPC a C18:2	19.16	7.87	21.17	6.97	8.17E-10	1.15E-08
lysoPC a C28:1	0.39	0.11	0.43	0.13	7.41E-10	1.15E-08
PC ae C38:2	1.85	0.67	1.98	0.54	2.37E-09	3.04E-08
lysoPC a C18:0	14.44	5.15	16.08	6.07	4.48E-09	5.27E-08
PC ae C30:0	0.32	0.08	0.35	0.11	7.59E-09	8.23E-08
PC ae C34:0	1.14	0.27	1.25	0.36	4.54E-08	4.54E-07
SM (OH) C14:1	6.01	1.51	6.48	1.72	4.83E-08	4.54E-07
PC ae C40:2	1.40	0.41	1.49	0.38	6.87E-08	6.06E-07
PC ae C36:2	14.91	3.72	16.00	4.16	2.30E-07	1.91E-06
SM C26:1	0.22	0.10	0.24	0.10	7.33E-07	5.75E-06
PC ae C38:0	1.85	0.57	2.09	0.80	1.06E-06	7.83E-06
lysoPC a C16:0	55.31	19.08	60.25	21.52	2.43E-06	1.71E-05
PC ae C36:0	0.90	0.24	0.94	0.22	4.87E-06	3.27E-05
Serotonin	0.31	0.49	0.24	0.32	6.99E-06	4.48E-05
SM C16:0	113.48	22.63	118.42	25.74	1.08E-05	6.61E-05
SM C24:0	21.71	5.55	23.18	5.79	1.80E-05	1.06E-04
SM (OH) C16:1	3.71	0.92	3.91	1.00	2.46E-05	1.39E-04
PC ae C42:2	0.46	0.14	0.49	0.13	3.52E-05	1.84E-04
Orn	70.30	20.71	72.56	24.21	3.41E-05	1.84E-04
lysoPC a C26:1	0.11	0.04	0.12	0.05	3.84E-05	1.93E-04
lysoPC a C24:0	0.15	0.05	0.16	0.05	5.25E-05	2.55E-04
SM (OH) C22:2	12.25	3.26	12.88	3.30	5.78E-05	2.72E-04
PC ae C44:4	0.24	0.06	0.25	0.06	6.06E-05	2.76E-04
lysoPC a C16:1	1.89	0.82	2.05	0.78	7.64E-05	3.37E-04
PC aa C36:6	0.73	0.29	0.83	0.44	9.26E-05	3.96E-04
PC ae C40:3	1.02	0.69	0.99	0.27	1.11E-04	4.60E-04
PC ae C42:3	0.56	0.17	0.59	0.15	1.14E-04	4.60E-04
PC aa C36:1	39.06	12.22	40.62	11.34	1.56E-04	6.13E-04
Taurine	58.67	40.26	50.51	34.48	1.68E-04	6.39E-04
PC aa C24:0	0.07	0.02	0.07	0.03	1.93E-04	7.17E-04
SM C24:1	46.63	11.45	48.36	12.10	2.33E-04	8.41E-04
SM C26:0	0.20	0.09	0.21	0.10	2.81E-04	9.89E-04
PC ae C34:2	13.36	3.74	13.98	3.85	3.14E-04	1.08E-03
lysoPC a C20:4	4.50	1.68	4.86	1.76	3.27E-04	1.10E-03
PC aa C30:0	2.91	1.03	3.21	1.29	4.28E-04	1.40E-03
Gly	261.90	119.66	277.97	115.71	4.50E-04	1.44E-03

Hexose	5927.76	1054.95	5731.62	993.10	4.69E-04	1.47E-03
PC aa C36:5	19.84	8.39	23.34	14.81	7.90E-04	2.42E-03
PC aa C32:2	4.27	2.03	4.68	2.07	8.08E-04	2.42E-03
PC ae C42:4	0.65	0.18	0.66	0.17	1.17E-03	3.44E-03
Pro	212.99	57.02	218.96	62.32	1.30E-03	3.73E-03
PC aa C36:2	297.80	66.81	305.94	70.00	1.45E-03	4.10E-03
AC4	0.22	0.09	0.23	0.12	1.61E-03	4.44E-03
Thr	138.91	42.96	126.74	41.13	2.29E-03	6.22E-03
PC ae C32:1	2.12	0.49	2.17	0.49	2.66E-03	7.06E-03
lysoPC a C26:0	0.27	0.09	0.27	0.08	2.94E-03	7.69E-03
Phe	66.88	12.01	68.05	14.10	3.43E-03	8.79E-03
PC ae C40:5	2.60	0.72	2.67	0.68	3.63E-03	9.15E-03
PC ae C40:6	3.36	0.91	3.55	1.06	3.87E-03	9.59E-03
His	97.20	31.78	100.71	31.47	4.28E-03	1.04E-02
Val	220.23	61.32	225.33	59.41	4.40E-03	1.05E-02
Tyr	76.60	21.45	78.25	21.33	4.59E-03	1.08E-02
Ser	123.28	48.98	131.64	51.86	4.92E-03	1.13E-02
alpha-AAA	1.08	0.49	1.08	0.49	4.97E-03	1.13E-02
PC aa C34:4	2.03	0.70	2.13	0.71	6.85E-03	1.53E-02
Cit	31.32	10.95	33.78	13.46	6.98E-03	1.54E-02
Spermidine	0.21	0.08	0.20	0.06	7.12E-03	1.54E-02
AC8	0.28	0.09	0.29	0.07	8.22E-03	1.70E-02
PC aa C42:5	0.25	0.07	0.27	0.09	8.06E-03	1.70E-02
PC ae C32:2	0.62	0.16	0.63	0.15	8.17E-03	1.70E-02
Creatinine	70.65	15.91	68.72	15.03	1.05E-02	2.15E-02
lysoPC a C28:0	0.25	0.12	0.26	0.11	1.13E-02	2.23E-02
PC aa C34:3	22.10	6.21	22.60	6.20	1.14E-02	2.23E-02
SM C18:1	13.47	3.48	12.97	3.31	1.12E-02	2.23E-02
PC aa C40:2	0.28	0.14	0.29	0.09	1.49E-02	2.89E-02
PC ae C42:5	1.43	0.35	1.44	0.33	1.81E-02	3.45E-02
PC ae C40:4	2.17	0.63	2.18	0.53	2.56E-02	4.81E-02

Table S2. Differential analytes between IBF and IFF/Mixed women at follow-up.

Metabolites	IFF/Mixed_Mean	IFF/Mixed_SD	IBF_Mean	IBF_SD	P-value	FDR
His	74.55	17.45	79.53	15.81	8.73E-06	1.27E-03
Cit	22.25	9.18	24.74	9.81	1.81E-05	1.31E-03
total-DMA	0.59	0.36	0.66	0.37	9.99E-04	4.83E-02

Table S3. Differential lipid species between IBF and IFF/Mixed women at baseline.

Lipid species	IFF/Mixed_Mean	IFF/Mixed_SD	IBF_Mean	IBF_SD	P-value	FDR
DAG(16:0/22:5)	0.06	0.04	0.05	0.04	7.33E-25	5.99E-22
DAG(18:0/18:2)	0.73	0.55	0.53	0.44	3.28E-23	1.34E-20
DAG(18:2/22:5)	0.09	0.06	0.07	0.04	3.35E-21	9.13E-19
DAG(18:1/18:2)	6.51	4.42	4.74	3.14	1.69E-20	3.46E-18
CE(15:0)	9.09	7.28	10.01	6.14	2.50E-20	4.10E-18
DAG(16:0/18:2)	2.33	1.90	1.70	1.53	2.92E-19	3.98E-17
TAG56:4-FA18:0	1.08	0.75	0.84	0.63	1.21E-18	1.42E-16
DAG(18:1/20:1)	0.10	0.06	0.08	0.04	3.08E-18	2.80E-16
TAG56:6-FA22:4	1.77	1.41	1.35	1.26	2.86E-18	2.80E-16
CE(22:0)	0.82	0.54	0.94	0.67	8.65E-18	7.08E-16
LPC(15:0)	0.69	0.28	0.81	0.33	1.73E-17	1.29E-15
TAG54:3-FA16:0	6.31	4.60	4.83	4.50	7.03E-17	4.79E-15
DAG(18:1/22:5)	0.12	0.07	0.09	0.05	1.03E-16	6.47E-15
TAG54:4-FA16:0	8.46	6.93	6.44	6.12	3.03E-16	1.77E-14
DAG(16:1/18:2)	0.74	0.62	0.53	0.43	6.95E-16	3.79E-14
TAG54:4-FA20:2	3.09	2.29	2.34	2.01	8.34E-16	4.27E-14
CER(24:0)	2.82	0.96	3.11	1.03	9.69E-16	4.66E-14
TAG52:2-FA18:2	37.88	29.20	29.31	28.20	1.51E-15	6.19E-14
TAG52:3-FA18:2	291.73	198.47	228.44	178.89	1.39E-15	6.19E-14
TAG56:5-FA22:4	2.17	1.75	1.68	1.61	1.50E-15	6.19E-14
TAG52:3-FA20:0	0.88	0.61	0.69	0.56	1.61E-15	6.28E-14
DAG(18:1/18:1)	5.84	4.06	4.40	2.94	1.70E-15	6.31E-14
TAG52:3-FA16:0	317.18	213.97	250.29	203.94	5.99E-15	2.13E-13
TAG56:5-FA16:0	1.92	1.56	1.50	1.44	7.78E-15	2.65E-13
CE(18:2)	1827.87	980.09	1907.20	1067.99	1.40E-14	4.56E-13
CE(17:0)	9.22	6.36	9.88	6.26	1.51E-14	4.75E-13
TAG54:2-FA20:2	0.49	0.38	0.38	0.37	1.80E-14	5.45E-13
DAG(16:0/18:1)	4.38	3.63	3.26	3.02	4.25E-14	1.24E-12
TAG56:4-FA16:0	0.44	0.38	0.34	0.34	5.13E-14	1.45E-12
TAG54:5-FA16:0	8.59	7.38	6.73	6.51	6.19E-14	1.69E-12
CE(18:1)	688.71	406.53	715.12	406.04	6.43E-14	1.70E-12
LPC(17:0)	1.16	0.46	1.32	0.57	7.31E-14	1.85E-12
TAG54:5-FA20:3	5.67	4.64	4.41	3.90	7.45E-14	1.85E-12
DAG(18:0/18:1)	1.16	0.94	0.86	0.76	7.94E-14	1.91E-12
TAG56:3-FA18:0	0.54	0.39	0.41	0.32	1.03E-13	2.41E-12
TAG52:4-FA22:1	0.34	0.23	0.26	0.20	2.05E-13	4.66E-12
CE(18:3)	71.68	47.85	75.56	47.50	2.56E-13	5.66E-12
TAG56:7-FA22:5	4.18	2.82	3.35	2.76	3.31E-13	7.12E-12
TAG52:3-FA18:1	321.99	219.87	254.59	197.44	3.40E-13	7.14E-12
TAG56:4-FA22:4	0.27	0.24	0.21	0.22	3.77E-13	7.71E-12
LCER(14:0)	0.11	0.06	0.12	0.10	4.31E-13	8.61E-12
TAG52:3-FA22:1	0.21	0.14	0.17	0.13	5.45E-13	1.06E-11
TAG54:3-FA20:2	3.86	2.89	3.01	2.66	5.70E-13	1.06E-11
TAG56:5-FA18:0	1.92	1.18	1.55	1.08	5.67E-13	1.06E-11

DAG(18:1/20:2)	0.11	0.06	0.09	0.04	8.23E-13	1.47E-11
TAG52:4-FA16:0	136.51	97.51	104.33	89.58	8.25E-13	1.47E-11
TAG54:3-FA20:1	2.39	1.77	1.84	1.53	1.06E-12	1.85E-11
TAG54:5-FA20:2	0.61	0.46	0.47	0.38	1.11E-12	1.85E-11
TAG56:7-FA22:4	0.28	0.22	0.22	0.18	1.09E-12	1.85E-11
PC(17:0/18:1)	2.00	0.76	2.19	0.79	1.62E-12	2.65E-11
DAG(16:0/20:3)	0.13	0.09	0.10	0.08	2.61E-12	4.18E-11
TAG56:6-FA22:5	5.03	3.36	4.13	3.42	3.74E-12	5.88E-11
TAG52:4-FA18:2	213.86	149.77	163.73	135.43	4.10E-12	6.32E-11
TAG52:3-FA18:0	5.19	4.18	3.97	3.77	4.28E-12	6.48E-11
TAG52:2-FA18:0	26.62	22.27	20.58	21.91	4.46E-12	6.63E-11
PC(15:0/18:2)	1.79	0.72	1.94	0.70	7.22E-12	1.06E-10
CE(18:0)	55.97	34.38	58.57	36.89	7.84E-12	1.12E-10
TAG56:6-FA16:0	3.73	2.73	3.03	2.62	8.24E-12	1.16E-10
SM(14:0)	19.05	6.10	20.75	6.96	8.94E-12	1.22E-10
TAG54:3-FA18:0	25.22	18.29	19.89	15.90	8.96E-12	1.22E-10
TAG56:6-FA18:2	3.42	2.32	2.66	1.84	9.27E-12	1.24E-10
TAG54:5-FA22:5	1.31	1.24	1.05	1.25	1.14E-11	1.51E-10
DCER(24:0)	0.58	0.22	0.62	0.21	1.17E-11	1.52E-10
TAG52:4-FA20:0	1.09	0.80	0.84	0.70	1.37E-11	1.75E-10
CER(26:1)	0.02	0.01	0.03	0.01	1.49E-11	1.88E-10
TAG54:4-FA22:4	0.61	0.68	0.48	0.65	1.62E-11	2.00E-10
TAG58:6-FA22:4	0.41	0.25	0.33	0.22	2.13E-11	2.61E-10
TAG52:4-FA20:2	0.46	0.36	0.36	0.28	2.41E-11	2.90E-10
TAG56:5-FA22:5	0.65	0.49	0.54	0.50	2.52E-11	2.95E-10
TAG58:7-FA18:2	0.43	0.27	0.35	0.24	2.50E-11	2.95E-10
DAG(18:2/22:6)	0.09	0.08	0.07	0.07	2.61E-11	2.99E-10
TAG56:5-FA18:1	5.13	3.39	4.13	2.87	2.63E-11	2.99E-10
DCER(20:0)	0.08	0.04	0.09	0.07	4.33E-11	4.86E-10
TAG56:4-FA20:4	0.59	0.37	0.49	0.34	5.13E-11	5.67E-10
TAG54:4-FA20:3	6.79	5.37	5.41	4.72	5.92E-11	6.46E-10
TAG54:5-FA16:1	1.27	1.04	0.98	0.80	6.29E-11	6.77E-10
TAG54:4-FA16:1	0.85	0.63	0.66	0.50	6.56E-11	6.97E-10
TAG54:3-FA20:3	0.90	0.81	0.71	0.73	6.68E-11	7.01E-10
DAG(16:0/16:1)	0.55	0.62	0.39	0.49	7.21E-11	7.38E-10
TAG54:3-FA18:2	25.81	18.86	20.29	16.00	7.15E-11	7.38E-10
TAG53:3-FA16:0	1.44	1.32	1.09	1.03	7.42E-11	7.49E-10
TAG50:2-FA18:2	69.36	65.04	53.60	61.27	7.55E-11	7.53E-10
DAG(16:1/20:2)	0.06	0.03	0.05	0.03	9.65E-11	9.51E-10
CE(14:0)	31.83	25.56	34.35	26.47	1.03E-10	1.00E-09
CER(26:0)	0.06	0.02	0.06	0.02	1.17E-10	1.12E-09
LPC(18:1)	14.05	5.94	15.19	5.30	1.23E-10	1.17E-09
TAG54:2-FA18:2	3.30	2.75	2.62	2.52	1.47E-10	1.38E-09
DAG(16:0/16:0)	1.02	1.06	0.76	0.91	1.76E-10	1.63E-09
DAG(18:2/20:3)	0.20	0.13	0.16	0.09	2.78E-10	2.56E-09

PI(18:1/20:4)	1.37	0.67	1.46	0.64	3.00E-10	2.72E-09
PE(P-18:1/16:0)	0.31	0.13	0.36	0.13	3.04E-10	2.73E-09
TAG58:6-FA18:0	0.26	0.18	0.21	0.15	3.25E-10	2.89E-09
LPC(18:0)	21.85	8.08	23.57	9.30	3.63E-10	3.20E-09
CE(20:5)	25.63	17.47	32.39	35.52	4.08E-10	3.55E-09
TAG54:4-FA18:0	11.99	9.19	9.16	7.23	5.47E-10	4.71E-09
LPC(14:0)	0.74	0.35	0.80	0.35	5.55E-10	4.72E-09
TAG56:8-FA22:5	0.67	0.44	0.54	0.41	5.60E-10	4.72E-09
CER(22:0)	1.06	0.37	1.11	0.36	8.09E-10	6.75E-09
DAG(18:0/18:3)	0.10	0.07	0.07	0.05	9.30E-10	7.68E-09
TAG56:5-FA18:2	1.88	1.36	1.43	1.01	9.85E-10	8.06E-09
TAG58:7-FA22:4	0.35	0.23	0.28	0.18	1.10E-09	8.94E-09
TAG53:4-FA16:0	0.50	0.48	0.38	0.37	1.21E-09	9.72E-09
TAG56:7-FA16:0	3.09	2.14	2.57	2.27	1.35E-09	1.07E-08
PI(18:1/18:1)	1.90	0.99	2.07	0.92	1.38E-09	1.08E-08
TAG52:2-FA16:0	259.16	185.91	208.84	175.56	1.44E-09	1.12E-08
TAG54:4-FA20:1	0.47	0.34	0.36	0.28	1.53E-09	1.18E-08
TAG54:2-FA20:1	3.03	2.20	2.40	2.03	1.67E-09	1.28E-08
HCER(20:0)	0.08	0.03	0.09	0.03	1.92E-09	1.45E-08
PC(14:0/18:1)	5.08	2.19	5.49	2.04	2.14E-09	1.61E-08
PC(18:1/20:5)	1.71	0.95	2.21	1.57	2.55E-09	1.89E-08
TAG54:5-FA22:4	0.63	0.71	0.47	0.55	2.56E-09	1.89E-08
TAG52:4-FA18:1	53.80	37.08	42.00	31.87	2.60E-09	1.90E-08
PC(17:0/18:2)	4.65	1.65	4.97	1.57	2.90E-09	2.10E-08
TAG54:6-FA16:0	4.74	4.18	3.83	3.54	3.29E-09	2.36E-08
TAG52:2-FA20:2	1.04	1.01	0.81	0.96	4.00E-09	2.85E-08
PC(15:0/18:1)	1.16	0.57	1.27	0.53	4.73E-09	3.30E-08
TAG54:2-FA16:0	3.46	2.67	2.73	2.44	4.73E-09	3.30E-08
LPC(18:2)	20.93	9.04	22.49	8.13	5.09E-09	3.50E-08
TAG56:4-FA20:3	0.87	0.58	0.72	0.50	5.10E-09	3.50E-08
CE(24:0)	0.25	0.44	0.24	0.21	5.46E-09	3.70E-08
TAG52:4-FA16:1	35.61	24.21	27.95	19.70	5.47E-09	3.70E-08
PI(18:0/20:4)	20.66	7.30	21.41	7.40	6.09E-09	4.09E-08
TAG50:2-FA16:0	182.68	176.48	140.16	159.85	6.39E-09	4.25E-08
TAG58:6-FA22:5	0.34	0.20	0.28	0.20	6.67E-09	4.40E-08
CE(16:0)	434.05	275.80	441.50	282.84	8.19E-09	5.36E-08
TAG52:2-FA18:1	410.87	284.39	335.77	267.66	1.00E-08	6.50E-08
DAG(18:2/20:4)	0.46	0.35	0.36	0.24	1.07E-08	6.87E-08
TAG56:3-FA20:2	0.29	0.19	0.24	0.16	1.10E-08	7.00E-08
TAG54:1-FA20:1	0.40	0.33	0.32	0.29	1.15E-08	7.31E-08
TAG52:1-FA18:1	55.11	43.20	44.47	44.05	1.29E-08	8.14E-08
TAG50:3-FA18:2	69.44	60.33	53.92	50.38	1.34E-08	8.34E-08
PC(18:1/18:1)	16.26	6.02	17.35	5.96	1.37E-08	8.47E-08
LPC(16:1)	2.36	1.09	2.49	0.89	1.40E-08	8.64E-08
DAG(16:0/20:4)	0.23	0.21	0.18	0.17	1.50E-08	9.09E-08

HCER(22:1)	0.07	0.03	0.08	0.03	1.50E-08	9.09E-08
TAG50:3-FA16:0	54.36	54.23	41.35	46.01	1.51E-08	9.09E-08
LPC(18:3)	0.30	0.17	0.32	0.16	1.58E-08	9.38E-08
PI(18:0/18:1)	2.14	0.94	2.26	0.87	1.58E-08	9.38E-08
TAG52:3-FA20:2	1.35	1.25	1.05	1.02	1.59E-08	9.38E-08
TAG50:3-FA16:1	47.72	46.21	36.07	36.75	1.62E-08	9.46E-08
TAG52:5-FA18:2	33.56	24.41	25.56	20.55	1.73E-08	1.00E-07
HCER(24:0)	0.79	0.25	0.85	0.25	1.88E-08	1.09E-07
LCER(24:1)	0.19	0.07	0.21	0.07	2.02E-08	1.15E-07
TAG58:6-FA18:1	0.61	0.39	0.51	0.34	2.18E-08	1.24E-07
CER(24:1)	1.05	0.37	1.09	0.35	2.25E-08	1.26E-07
TAG52:4-FA18:0	0.55	0.44	0.43	0.38	2.24E-08	1.26E-07
DAG(16:0/18:3)	0.24	0.21	0.18	0.17	2.54E-08	1.42E-07
DAG(16:0/22:6)	0.05	0.04	0.04	0.04	2.62E-08	1.44E-07
HCER(16:0)	0.90	0.27	0.95	0.27	2.62E-08	1.44E-07
LCER(20:1)	0.03	0.01	0.04	0.01	2.67E-08	1.45E-07
TAG52:5-FA16:1	15.61	10.92	11.96	8.79	3.41E-08	1.85E-07
DAG(16:1/18:0)	0.26	0.20	0.21	0.16	3.53E-08	1.90E-07
CE(20:0)	1.73	3.14	1.76	2.66	3.60E-08	1.91E-07
TAG56:5-FA20:4	4.48	2.61	3.70	2.41	3.58E-08	1.91E-07
TAG54:6-FA22:5	1.31	1.18	1.06	1.04	3.95E-08	2.09E-07
TAG52:3-FA20:3	1.92	2.14	1.51	1.92	4.07E-08	2.14E-07
TAG56:7-FA16:1	0.33	0.22	0.28	0.19	4.14E-08	2.16E-07
TAG56:8-FA16:0	1.18	0.92	1.00	1.04	4.25E-08	2.20E-07
HCER(24:1)	0.58	0.19	0.62	0.20	4.65E-08	2.39E-07
TAG52:2-FA16:1	6.51	5.15	5.14	4.58	4.81E-08	2.46E-07
TAG56:6-FA18:0	1.27	0.78	1.04	0.71	5.18E-08	2.63E-07
TAG56:4-FA18:1	2.90	2.00	2.31	1.55	5.27E-08	2.66E-07
PI(18:0/18:2)	7.51	2.61	7.89	2.77	5.87E-08	2.95E-07
CE(18:4)	1.41	1.23	1.52	1.28	5.96E-08	2.97E-07
TAG56:6-FA18:3	0.32	0.22	0.25	0.18	6.12E-08	3.03E-07
DAG(14:0/18:2)	0.35	0.34	0.27	0.25	6.42E-08	3.16E-07
HCER(14:0)	0.02	0.01	0.02	0.01	6.76E-08	3.31E-07
PC(18:2/18:2)	21.30	7.91	22.75	8.13	7.09E-08	3.43E-07
TAG52:3-FA20:1	0.41	0.32	0.32	0.25	7.08E-08	3.43E-07
TAG52:1-FA16:0	45.10	36.96	36.36	38.87	7.59E-08	3.65E-07
PE(18:0/22:4)	1.21	0.74	1.01	0.57	8.06E-08	3.83E-07
TAG55:1-FA16:0	1.25	0.98	1.06	1.12	8.05E-08	3.83E-07
TAG54:6-FA22:6	1.49	1.73	1.25	1.77	9.69E-08	4.58E-07
TAG52:4-FA18:3	30.47	22.75	24.19	21.57	9.80E-08	4.61E-07
PC(18:0/18:1)	43.21	15.99	44.70	13.91	1.04E-07	4.86E-07
HCER(22:0)	0.66	0.22	0.70	0.20	1.14E-07	5.28E-07
TAG58:10-FA20:5	0.15	0.11	0.19	0.16	1.16E-07	5.37E-07
TAG52:1-FA20:1	0.82	0.74	0.64	0.71	1.32E-07	6.09E-07
SM(24:0)	32.90	7.66	35.00	7.91	1.41E-07	6.45E-07

PC(14:0/18:2)	7.65	2.75	8.14	2.82	1.47E-07	6.69E-07
PE(P-18:1/18:1)	1.01	0.38	1.10	0.42	1.50E-07	6.76E-07
TAG52:5-FA16:0	17.68	14.85	13.51	13.09	1.51E-07	6.81E-07
SM(26:0)	0.50	0.13	0.53	0.13	1.60E-07	7.10E-07
TAG54:6-FA20:4	13.54	11.64	11.07	9.66	1.59E-07	7.10E-07
DAG(18:2/18:3)	0.39	0.31	0.29	0.21	1.61E-07	7.11E-07
TAG58:5-FA18:1	0.21	0.14	0.18	0.13	1.69E-07	7.42E-07
DCER(16:0)	0.13	0.15	0.14	0.18	1.70E-07	7.45E-07
DAG(14:0/18:1)	0.77	0.66	0.60	0.50	1.73E-07	7.53E-07
TAG52:1-FA18:0	34.14	29.66	27.42	31.39	1.83E-07	7.90E-07
TAG52:3-FA18:3	3.91	3.18	3.08	3.07	2.09E-07	8.99E-07
TAG56:8-FA22:6	4.84	4.29	4.16	4.89	2.30E-07	9.83E-07
PC(18:0/20:5)	7.15	3.75	9.04	6.82	2.32E-07	9.90E-07
TAG56:6-FA22:6	0.76	0.72	0.65	0.79	2.56E-07	1.08E-06
LPC(16:0)	82.33	28.77	86.49	31.67	2.71E-07	1.14E-06
DAG(18:1/20:4)	0.65	0.46	0.53	0.33	2.76E-07	1.16E-06
TAG54:4-FA20:4	2.37	2.07	1.96	1.90	3.02E-07	1.26E-06
DAG(16:0/18:0)	0.84	0.60	0.67	0.50	3.31E-07	1.37E-06
PC(16:0/18:3)	9.97	4.93	10.16	4.40	3.40E-07	1.41E-06
LCER(24:0)	0.10	0.04	0.11	0.03	3.47E-07	1.43E-06
TAG50:1-FA16:0	187.57	180.25	148.30	182.93	3.63E-07	1.49E-06
TAG54:7-FA22:5	0.37	0.28	0.31	0.25	3.89E-07	1.58E-06
TAG50:4-FA14:1	1.34	1.09	1.05	0.72	3.97E-07	1.61E-06
TAG50:1-FA18:1	107.03	102.00	85.25	101.74	4.16E-07	1.68E-06
CE(24:1)	0.16	0.16	0.16	0.12	4.55E-07	1.83E-06
PC(16:0/20:1)	1.44	0.50	1.53	0.50	4.88E-07	1.94E-06
PC(18:1/16:1)	9.78	3.76	10.09	3.28	4.85E-07	1.94E-06
TAG52:5-FA22:5	0.78	0.72	0.62	0.64	5.12E-07	2.02E-06
TAG56:3-FA16:0	0.21	0.17	0.17	0.18	5.52E-07	2.17E-06
PI(16:0/18:1)	1.96	1.12	2.01	0.93	5.85E-07	2.28E-06
SM(16:0)	188.81	47.49	195.33	46.53	5.85E-07	2.28E-06
TAG54:5-FA22:1	0.38	0.30	0.29	0.21	6.68E-07	2.59E-06
TAG54:2-FA18:0	20.88	16.77	17.25	17.96	6.97E-07	2.69E-06
PC(18:1/22:5)	1.83	0.76	1.96	0.72	7.45E-07	2.86E-06
DAG(18:1/20:3)	0.28	0.17	0.23	0.13	7.72E-07	2.95E-06
TAG54:3-FA16:1	0.46	0.34	0.36	0.27	7.88E-07	3.00E-06
TAG54:6-FA20:3	1.00	0.80	0.80	0.63	8.17E-07	3.09E-06
TAG52:3-FA16:1	32.14	23.08	25.68	18.56	8.35E-07	3.15E-06
TAG52:4-FA22:4	0.28	0.30	0.22	0.25	8.91E-07	3.33E-06
TAG54:5-FA20:4	15.80	13.48	13.08	11.86	8.87E-07	3.33E-06
PE(P-18:0/20:5)	1.49	1.03	1.83	1.68	1.03E-06	3.85E-06
TAG54:4-FA18:2	73.71	53.49	57.93	40.16	1.05E-06	3.88E-06
TAG56:7-FA22:6	5.67	5.14	4.91	5.63	1.10E-06	4.07E-06
TAG58:7-FA18:0	0.24	0.15	0.21	0.16	1.20E-06	4.40E-06
TAG52:1-FA16:1	0.67	0.58	0.54	0.54	1.25E-06	4.57E-06

PC(18:0/18:3)	3.28	1.89	3.41	1.75	1.35E-06	4.91E-06
LCER(16:0)	2.11	0.61	2.21	0.64	1.43E-06	5.16E-06
TAG52:4-FA20:3	2.16	2.22	1.68	1.73	1.51E-06	5.43E-06
PC(18:1/18:2)	83.60	27.86	87.83	28.37	1.58E-06	5.68E-06
TAG58:6-FA16:0	0.20	0.15	0.16	0.16	1.67E-06	5.95E-06
TAG54:5-FA18:0	1.81	1.53	1.39	1.22	2.06E-06	7.34E-06
DAG(18:1/22:6)	0.12	0.10	0.10	0.10	2.09E-06	7.41E-06
PC(18:2/20:4)	11.73	3.24	12.35	3.22	2.12E-06	7.49E-06
TAG52:2-FA20:1	0.88	0.82	0.68	0.64	2.21E-06	7.75E-06
DAG(16:1/20:4)	0.11	0.12	0.09	0.08	2.55E-06	8.92E-06
TAG53:3-FA18:2	5.51	4.32	4.36	3.10	2.57E-06	8.95E-06
TAG50:4-FA16:1	9.04	8.24	6.85	6.33	3.07E-06	1.07E-05
PE(P-18:1/18:2)	3.16	1.27	3.42	1.55	3.15E-06	1.09E-05
PC(18:2/18:3)	1.20	0.43	1.24	0.41	3.28E-06	1.12E-05
TAG50:4-FA18:2	18.78	14.98	14.65	12.04	3.26E-06	1.12E-05
LCER(22:0)	0.11	0.05	0.11	0.04	3.74E-06	1.28E-05
TAG52:5-FA18:3	22.05	17.45	17.18	15.16	3.77E-06	1.28E-05
TAG53:2-FA16:0	1.77	1.73	1.39	1.41	3.87E-06	1.31E-05
TAG54:5-FA18:1	51.69	39.98	39.76	28.60	4.01E-06	1.35E-05
LCER(22:1)	0.04	0.01	0.04	0.01	4.06E-06	1.36E-05
PC(16:0/18:1)	258.63	90.87	260.40	79.92	4.86E-06	1.61E-05
TAG50:1-FA16:1	8.62	9.39	6.58	8.35	4.83E-06	1.61E-05
TAG50:2-FA16:1	65.36	64.96	50.51	54.33	4.83E-06	1.61E-05
PC(16:0/14:0)	3.25	1.76	3.54	1.73	6.00E-06	1.98E-05
PC(18:0/18:2)	275.64	79.13	282.99	75.29	6.03E-06	1.98E-05
TAG50:0-FA16:0	26.26	27.52	20.51	27.89	6.30E-06	2.06E-05
TAG54:5-FA18:2	66.24	54.34	49.97	36.35	6.39E-06	2.08E-05
TAG54:6-FA16:1	1.30	1.05	1.08	0.84	6.48E-06	2.10E-05
PE(P-16:0/18:1)	1.25	0.53	1.33	0.52	6.89E-06	2.23E-05
TAG54:2-FA18:1	38.13	30.12	31.73	30.73	7.08E-06	2.28E-05
PC(18:1/18:3)	1.33	0.66	1.39	0.64	7.22E-06	2.32E-05
PI(18:1/18:2)	1.30	0.55	1.39	0.64	7.37E-06	2.36E-05
SM(24:1)	67.11	15.89	68.50	15.57	7.51E-06	2.39E-05
CER(22:1)	0.06	0.02	0.06	0.02	7.65E-06	2.43E-05
DCER(24:1)	0.40	0.12	0.41	0.12	7.87E-06	2.49E-05
TAG54:4-FA22:1	0.32	0.23	0.25	0.17	7.94E-06	2.50E-05
PC(16:0/20:5)	13.68	7.15	16.77	13.48	8.07E-06	2.53E-05
DCER(22:0)	0.30	0.10	0.31	0.10	9.30E-06	2.90E-05
TAG52:4-FA20:4	4.29	5.10	3.46	4.61	9.45E-06	2.94E-05
TAG54:4-FA18:3	3.06	2.28	2.42	1.95	9.67E-06	3.00E-05
TAG53:4-FA18:2	5.15	3.69	4.10	2.69	1.01E-05	3.10E-05
TAG55:5-FA18:2	0.44	0.50	0.32	0.24	1.01E-05	3.10E-05
HCER(18:1)	0.03	0.01	0.03	0.01	1.05E-05	3.20E-05
TAG56:2-FA18:0	0.36	0.30	0.29	0.28	1.21E-05	3.71E-05
TAG58:8-FA18:2	0.52	0.31	0.45	0.29	1.49E-05	4.53E-05

TAG52:0-FA18:0	5.25	5.10	4.14	5.03	1.51E-05	4.58E-05
TAG52:6-FA18:2	2.73	2.00	2.12	1.70	1.52E-05	4.58E-05
CE(20:1)	1.23	0.95	1.22	0.87	1.53E-05	4.59E-05
TAG56:6-FA20:2	0.46	0.32	0.36	0.25	1.53E-05	4.59E-05
TAG54:4-FA18:1	119.86	86.03	95.44	64.20	1.66E-05	4.96E-05
CE(22:1)	0.54	0.45	0.60	0.59	1.70E-05	5.05E-05
TAG52:0-FA16:0	3.88	3.73	3.12	3.92	1.72E-05	5.09E-05
CE(22:5)	3.36	1.87	3.42	1.90	1.75E-05	5.16E-05
TAG50:3-FA18:3	6.82	7.01	5.34	6.71	2.19E-05	6.44E-05
TAG53:3-FA17:0	5.10	3.83	4.10	2.84	2.22E-05	6.52E-05
TAG56:7-FA18:2	3.92	2.55	3.30	2.27	2.39E-05	6.99E-05
PC(18:2/16:1)	16.38	5.56	16.81	5.27	2.49E-05	7.22E-05
TAG55:4-FA18:1	0.75	0.68	0.58	0.42	2.48E-05	7.22E-05
SM(26:1)	0.66	0.16	0.68	0.16	2.51E-05	7.25E-05
TAG56:9-FA22:6	0.78	0.69	0.69	0.83	2.63E-05	7.58E-05
TAG56:4-FA20:2	0.97	0.64	0.80	0.51	2.68E-05	7.70E-05
TAG58:7-FA22:5	0.93	0.51	0.81	0.51	2.71E-05	7.74E-05
TAG52:5-FA18:1	5.09	3.62	4.03	3.02	2.79E-05	7.96E-05
PE(P-18:1/22:5)	0.98	0.29	1.03	0.36	2.81E-05	7.98E-05
PE(P-18:0/18:1)	1.68	0.67	1.78	0.67	2.87E-05	8.13E-05
TAG54:8-FA22:6	0.43	0.42	0.38	0.46	2.93E-05	8.27E-05
TAG50:3-FA18:1	33.31	27.25	26.56	21.37	3.04E-05	8.55E-05
TAG50:4-FA16:0	4.72	5.04	3.57	3.99	3.28E-05	9.19E-05
TAG51:2-FA18:2	3.43	3.41	2.68	2.45	3.56E-05	9.94E-05
TAG56:1-FA16:0	0.16	0.18	0.13	0.18	3.69E-05	1.03E-04
HCER(18:0)	0.12	0.04	0.12	0.03	3.73E-05	1.03E-04
TAG54:7-FA22:6	1.54	1.74	1.28	1.53	3.91E-05	1.08E-04
TAG53:4-FA17:0	2.19	1.72	1.72	1.24	4.00E-05	1.10E-04
TAG53:2-FA18:2	0.94	0.87	0.74	0.61	4.08E-05	1.12E-04
PE(P-18:0/16:0)	0.33	0.13	0.35	0.11	4.26E-05	1.16E-04
TAG56:6-FA18:1	5.88	3.75	5.07	3.28	4.46E-05	1.22E-04
CE(12:0)	1.07	0.94	1.28	1.27	4.57E-05	1.24E-04
PI(18:0/22:5)	0.48	0.19	0.52	0.25	4.78E-05	1.29E-04
TAG54:1-FA18:1	4.64	4.22	3.92	4.80	4.79E-05	1.29E-04
PC(18:2/20:3)	3.60	1.51	3.64	1.23	4.84E-05	1.29E-04
PE(O-18:0/18:2)	0.96	0.43	0.97	0.34	4.83E-05	1.29E-04
TAG52:3-FA14:0	0.53	0.44	0.42	0.36	4.81E-05	1.29E-04
PC(17:0/20:4)	2.43	0.98	2.55	0.99	4.99E-05	1.33E-04
TAG51:2-FA16:0	8.29	7.95	6.53	5.71	5.21E-05	1.38E-04
TAG52:4-FA14:0	0.71	0.64	0.56	0.52	5.37E-05	1.42E-04
TAG48:1-FA16:1	13.56	19.52	9.90	16.52	5.46E-05	1.44E-04
PE(18:0/20:5)	1.39	0.74	1.56	0.86	5.49E-05	1.44E-04
TAG51:2-FA17:0	3.70	3.73	2.90	2.62	5.52E-05	1.45E-04
TAG52:8-FA18:2	0.25	0.24	0.20	0.18	5.54E-05	1.45E-04
PC(20:0/18:1)	1.77	0.91	1.96	0.94	5.93E-05	1.55E-04

TAG50:4-FA14:0	9.89	8.07	7.80	6.67	6.98E-05	1.81E-04
CER(16:0)	0.38	0.12	0.38	0.11	7.08E-05	1.83E-04
TAG50:0-FA18:0	9.45	10.13	7.39	10.16	7.09E-05	1.83E-04
TAG54:6-FA18:2	30.70	30.08	22.56	18.92	7.17E-05	1.84E-04
PE(P-16:0/22:5)	3.30	1.17	3.46	1.28	7.29E-05	1.87E-04
TAG58:8-FA22:5	0.87	0.51	0.75	0.47	8.41E-05	2.15E-04
PI(16:0/20:4)	2.65	1.69	2.59	1.20	8.50E-05	2.17E-04
PE(P-18:0/18:2)	6.72	2.92	7.14	3.08	8.62E-05	2.19E-04
TAG50:5-FA18:2	2.04	1.72	1.59	1.37	8.64E-05	2.19E-04
TAG52:5-FA20:3	0.61	0.53	0.49	0.40	9.00E-05	2.27E-04
TAG50:2-FA18:1	114.11	104.34	90.83	84.93	9.26E-05	2.32E-04
TAG51:4-FA16:1	0.59	0.49	0.46	0.33	9.25E-05	2.32E-04
TAG55:7-FA15:0	0.32	0.21	0.32	0.19	9.41E-05	2.34E-04
TAG56:5-FA20:2	0.95	0.64	0.77	0.50	9.41E-05	2.34E-04
TAG56:7-FA20:5	1.05	0.69	1.16	0.92	9.43E-05	2.34E-04
CER(14:0)	0.02	0.01	0.02	0.01	9.68E-05	2.39E-04
TAG54:1-FA18:0	5.45	5.40	4.64	7.59	9.67E-05	2.39E-04
TAG51:3-FA18:2	8.14	7.13	6.48	4.78	1.22E-04	3.01E-04
TAG50:5-FA14:1	0.67	0.52	0.54	0.35	1.30E-04	3.20E-04
TAG58:10-FA20:4	0.64	0.51	0.68	0.47	1.36E-04	3.34E-04
DAG(14:0/20:0)	0.09	0.16	0.12	0.46	1.38E-04	3.36E-04
LPE(22:6)	0.15	0.06	0.16	0.07	1.38E-04	3.36E-04
TAG53:2-FA18:0	1.14	0.80	0.96	0.58	1.42E-04	3.44E-04
TAG51:3-FA17:0	1.00	0.96	0.79	0.66	1.43E-04	3.45E-04
TAG56:9-FA20:5	0.48	0.34	0.54	0.47	1.48E-04	3.56E-04
TAG56:2-FA16:0	0.21	0.21	0.18	0.26	1.51E-04	3.64E-04
PE(16:0/20:3)	0.56	0.42	0.46	0.28	1.56E-04	3.73E-04
TAG52:6-FA16:1	2.16	1.68	1.68	1.38	1.57E-04	3.74E-04
TAG55:4-FA18:2	0.58	0.59	0.44	0.32	1.57E-04	3.74E-04
PE(P-16:0/18:2)	4.36	2.05	4.62	2.11	1.59E-04	3.77E-04
DAG(16:1/16:1)	0.15	0.16	0.12	0.17	1.60E-04	3.78E-04
PC(18:1/22:6)	4.85	2.03	5.28	2.18	1.62E-04	3.83E-04
TAG58:7-FA22:6	0.46	0.38	0.43	0.48	1.67E-04	3.94E-04
TAG53:1-FA16:0	0.74	0.85	0.58	0.69	1.69E-04	3.98E-04
SM(22:0)	81.95	24.40	82.69	23.41	1.77E-04	4.15E-04
PI(18:0/20:3)	3.74	1.81	3.62	1.48	1.80E-04	4.22E-04
DAG(12:0/16:0)	0.16	0.08	0.14	0.09	2.09E-04	4.86E-04
TAG60:10-FA22:6	0.15	0.11	0.14	0.14	2.19E-04	5.09E-04
TAG54:3-FA18:1	128.96	90.26	106.12	75.52	2.27E-04	5.27E-04
DAG(16:1/18:3)	0.19	0.16	0.15	0.14	2.38E-04	5.51E-04
LPE(16:0)	1.23	0.55	1.25	0.44	2.46E-04	5.67E-04
TAG52:8-FA16:1	0.21	0.25	0.17	0.17	2.52E-04	5.79E-04
TAG56:5-FA20:1	0.46	0.38	0.35	0.25	2.81E-04	6.44E-04
PI(16:0/18:2)	3.31	1.74	3.31	1.51	3.15E-04	7.19E-04
TAG58:7-FA16:0	0.22	0.16	0.19	0.18	3.16E-04	7.20E-04

TAG58:9-FA22:5	0.44	0.28	0.38	0.25	3.17E-04	7.21E-04
CE(20:4)	257.74	146.39	259.97	149.16	3.19E-04	7.22E-04
TAG56:4-FA20:1	1.00	0.74	0.80	0.52	3.19E-04	7.22E-04
TAG48:3-FA14:1	2.65	3.10	1.95	2.03	3.22E-04	7.25E-04
LPC(20:4)	2.57	0.97	2.71	1.11	3.29E-04	7.40E-04
TAG48:0-FA16:0	35.42	46.74	26.78	42.57	3.30E-04	7.40E-04
CE(22:6)	13.63	8.81	14.03	8.98	3.64E-04	8.12E-04
TAG56:4-FA18:2	1.16	0.92	0.92	0.63	3.68E-04	8.19E-04
PE(O-18:0/18:1)	0.36	0.15	0.36	0.12	3.79E-04	8.42E-04
CE(20:2)	2.79	1.74	2.69	1.48	3.95E-04	8.73E-04
TAG52:2-FA20:0	0.21	0.16	0.18	0.14	3.95E-04	8.73E-04
SM(20:1)	6.84	1.69	6.97	1.81	4.02E-04	8.87E-04
TAG50:3-FA14:0	25.78	21.13	20.80	17.02	4.29E-04	9.44E-04
TAG50:2-FA20:2	0.37	0.42	0.29	0.35	4.37E-04	9.57E-04
TAG50:5-FA16:1	0.88	0.86	0.67	0.61	4.56E-04	9.98E-04
TAG56:7-FA18:3	0.52	0.36	0.42	0.29	4.57E-04	9.98E-04
PC(18:1/20:2)	1.36	0.60	1.36	0.48	4.62E-04	1.01E-03
TAG50:1-FA18:0	9.62	10.69	7.45	9.25	4.64E-04	1.01E-03
FFA(20:5)	5.82	2.04	6.14	2.11	4.96E-04	1.07E-03
TAG50:3-FA18:0	0.51	0.50	0.40	0.37	5.04E-04	1.09E-03
TAG48:4-FA16:1	0.67	0.73	0.49	0.47	5.17E-04	1.11E-03
TAG52:6-FA18:3	3.38	2.72	2.61	2.23	5.48E-04	1.18E-03
LPE(18:1)	1.03	0.51	1.04	0.50	5.77E-04	1.23E-03
TAG55:5-FA18:1	0.38	0.30	0.31	0.20	5.77E-04	1.23E-03
TAG56:8-FA20:5	1.00	0.65	1.10	0.86	5.88E-04	1.25E-03
TAG50:2-FA18:0	3.19	3.19	2.52	2.57	6.06E-04	1.29E-03
TAG50:3-FA14:1	1.46	1.28	1.19	0.92	6.20E-04	1.31E-03
PE(18:0/20:3)	4.28	2.10	3.67	1.73	6.32E-04	1.34E-03
TAG48:2-FA16:0	26.22	31.43	19.69	24.37	6.52E-04	1.37E-03
PC(16:0/22:5)	25.30	8.38	26.02	9.64	6.59E-04	1.39E-03
TAG52:2-FA14:0	0.30	0.25	0.24	0.21	6.74E-04	1.41E-03
TAG54:0-FA18:0	0.88	0.89	0.75	1.10	6.82E-04	1.43E-03
LCER(18:1)	0.15	0.03	0.16	0.03	7.40E-04	1.54E-03
TAG58:7-FA18:1	0.77	0.44	0.68	0.43	7.42E-04	1.55E-03
DAG(14:0/16:1)	0.11	0.12	0.08	0.08	7.69E-04	1.60E-03
TAG52:6-FA22:6	0.50	0.62	0.42	0.61	7.78E-04	1.61E-03
LPC(20:3)	1.86	0.82	1.85	0.72	8.43E-04	1.74E-03
TAG46:3-FA16:0	0.49	0.60	0.36	0.46	8.50E-04	1.75E-03
TAG56:8-FA16:1	0.29	0.20	0.26	0.21	8.54E-04	1.76E-03
TAG51:3-FA16:1	1.43	1.29	1.12	0.87	8.59E-04	1.76E-03
LCER(18:0)	0.12	0.03	0.12	0.03	8.65E-04	1.77E-03
TAG48:2-FA18:2	18.80	20.56	14.52	17.02	8.80E-04	1.80E-03
TAG48:2-FA16:1	14.83	19.32	10.88	13.80	8.93E-04	1.82E-03
TAG48:4-FA14:1	0.48	0.54	0.36	0.33	9.21E-04	1.87E-03
LCER(20:0)	0.04	0.01	0.04	0.01	9.24E-04	1.87E-03

PE(18:0/20:4)	19.31	8.56	17.12	7.09	9.36E-04	1.89E-03
PE(P-18:0/22:5)	2.30	0.88	2.36	0.83	9.51E-04	1.92E-03
FFA(15:0)	7.44	7.77	7.76	7.19	1.03E-03	2.08E-03
TAG50:4-FA18:1	3.43	2.87	2.73	2.20	1.08E-03	2.17E-03
CER(20:0)	0.14	0.05	0.14	0.05	1.09E-03	2.18E-03
TAG54:6-FA18:1	10.88	8.92	8.42	6.60	1.12E-03	2.23E-03
TAG58:9-FA20:4	0.82	0.60	0.84	0.53	1.15E-03	2.29E-03
PE(16:0/18:1)	1.85	1.22	1.59	0.84	1.16E-03	2.31E-03
TAG56:2-FA20:1	0.33	0.27	0.28	0.24	1.17E-03	2.32E-03
TAG48:3-FA14:0	4.81	5.10	3.63	3.80	1.20E-03	2.38E-03
MAG(18:1)	0.29	0.17	0.26	0.29	1.22E-03	2.41E-03
SM(18:0)	33.05	9.04	33.35	9.57	1.29E-03	2.54E-03
TAG48:1-FA16:0	56.67	72.33	42.92	61.47	1.30E-03	2.54E-03
TAG48:2-FA14:0	19.21	20.85	14.68	16.39	1.38E-03	2.71E-03
PC(16:0/18:0)	56.23	32.16	59.64	36.50	1.41E-03	2.75E-03
PE(18:0/22:6)	6.27	3.25	5.73	3.43	1.47E-03	2.86E-03
CE(14:1)	1.86	1.81	1.91	1.65	1.51E-03	2.93E-03
TAG50:4-FA18:3	6.94	6.74	5.43	5.47	1.54E-03	2.98E-03
TAG48:3-FA16:1	4.91	5.36	3.71	3.76	1.55E-03	2.99E-03
PE(P-18:1/20:3)	0.80	0.38	0.82	0.38	1.65E-03	3.19E-03
PC(20:0/18:2)	5.45	2.33	5.94	2.59	1.67E-03	3.21E-03
PE(18:2/16:1)	0.56	0.33	0.57	0.27	1.67E-03	3.21E-03
TAG54:7-FA18:2	7.05	7.40	5.21	4.83	1.68E-03	3.21E-03
DAG(16:1/18:1)	3.83	4.10	3.00	3.31	1.68E-03	3.21E-03
TAG51:1-FA17:0	4.19	4.30	3.36	3.31	1.72E-03	3.28E-03
TAG56:5-FA20:3	2.29	1.42	1.98	1.24	1.76E-03	3.34E-03
DAG(18:1/22:4)	0.05	0.03	0.04	0.02	1.77E-03	3.36E-03
PC(18:2/20:2)	0.94	0.38	0.98	0.37	1.79E-03	3.39E-03
PE(P-18:0/22:6)	6.37	2.98	6.82	3.60	1.87E-03	3.52E-03
TAG52:5-FA14:0	0.68	0.64	0.55	0.51	1.87E-03	3.52E-03
TAG52:5-FA20:4	4.27	5.36	3.41	3.76	1.92E-03	3.61E-03
TAG54:5-FA18:3	11.42	8.41	9.05	6.54	2.04E-03	3.83E-03
FFA(14:0)	21.64	6.39	21.34	5.65	2.08E-03	3.88E-03
PC(16:0/18:2)	677.96	188.28	676.01	196.46	2.08E-03	3.88E-03
FFA(22:2)	1.02	0.39	1.02	0.33	2.08E-03	3.88E-03
TAG54:6-FA18:3	11.40	9.45	8.78	6.94	2.09E-03	3.88E-03
PC(18:1/20:4)	28.11	9.98	29.07	10.13	2.11E-03	3.92E-03
TAG53:0-FA16:0	0.91	0.80	0.79	0.78	2.14E-03	3.95E-03
PC(14:0/20:3)	1.54	0.72	1.52	0.64	2.26E-03	4.17E-03
TAG48:3-FA16:0	4.39	5.12	3.29	3.80	2.28E-03	4.21E-03
MAG(18:3)	0.20	0.06	0.21	0.06	2.34E-03	4.31E-03
TAG56:3-FA20:1	1.03	0.74	0.85	0.57	2.39E-03	4.39E-03
TAG51:1-FA16:0	5.32	5.72	4.20	4.29	2.52E-03	4.61E-03
TAG48:4-FA16:0	0.44	0.56	0.33	0.37	2.55E-03	4.66E-03
TAG50:3-FA20:3	0.62	0.78	0.49	0.62	2.57E-03	4.67E-03

TAG55:3-FA18:1	0.99	0.90	0.79	0.63	2.72E-03	4.95E-03
TAG49:1-FA16:1	0.77	1.07	0.58	0.70	2.85E-03	5.16E-03
TAG55:7-FA20:3	0.22	0.03	0.22	0.02	2.89E-03	5.22E-03
TAG54:2-FA20:0	0.70	0.56	0.59	0.52	3.02E-03	5.46E-03
LPE(18:0)	1.19	0.52	1.18	0.40	3.10E-03	5.57E-03
TAG58:6-FA20:4	0.15	0.09	0.13	0.07	3.10E-03	5.57E-03
TAG51:4-FA18:2	3.51	2.90	2.83	2.05	3.14E-03	5.63E-03
LPE(20:3)	0.22	0.11	0.22	0.09	3.19E-03	5.70E-03
TAG53:5-FA18:1	6.52	0.51	6.56	0.42	3.29E-03	5.88E-03
PE(O-18:0/22:5)	0.59	0.22	0.60	0.22	3.55E-03	6.32E-03
MAG(18:2)	0.22	0.10	0.21	0.13	3.65E-03	6.50E-03
TAG50:2-FA14:0	22.98	19.68	18.86	16.10	4.08E-03	7.24E-03
FFA(22:0)	3.74	1.11	3.77	1.06	4.12E-03	7.29E-03
FFA(16:1)	17.72	6.94	17.72	6.75	4.23E-03	7.47E-03
TAG56:7-FA18:0	0.23	0.14	0.20	0.14	4.24E-03	7.47E-03
TAG58:9-FA22:6	1.19	0.98	1.14	1.31	4.59E-03	8.07E-03
PC(18:0/18:0)	3.14	1.61	3.16	1.58	4.61E-03	8.09E-03
TAG50:5-FA18:1	0.37	0.30	0.30	0.22	4.63E-03	8.09E-03
TAG53:6-FA18:1	1.94	0.14	1.95	0.15	4.63E-03	8.09E-03
LPE(22:5)	0.11	0.05	0.11	0.04	4.84E-03	8.44E-03
FFA(20:0)	7.51	3.83	7.51	2.49	4.85E-03	8.45E-03
TAG52:7-FA16:0	0.86	1.00	0.67	0.76	4.89E-03	8.49E-03
TAG53:2-FA18:1	8.37	6.68	6.92	4.93	5.04E-03	8.73E-03
TAG58:8-FA22:6	1.29	1.04	1.25	1.41	5.19E-03	8.98E-03
LPE(20:4)	0.79	0.32	0.79	0.33	5.29E-03	9.12E-03
DCER(18:0)	0.54	2.34	0.63	2.85	5.68E-03	9.78E-03
TAG50:2-FA14:1	0.41	0.43	0.34	0.32	5.86E-03	1.01E-02
SM(22:1)	42.46	11.71	42.06	10.78	5.90E-03	1.01E-02
PE(P-18:1/22:6)	3.53	1.55	3.73	2.04	5.97E-03	1.02E-02
TAG48:2-FA14:1	3.97	4.80	3.04	3.46	6.05E-03	1.03E-02
TAG53:1-FA17:0	0.89	0.86	0.73	0.65	6.09E-03	1.04E-02
TAG54:7-FA18:3	5.21	5.29	3.89	3.56	6.09E-03	1.04E-02
TAG55:6-FA18:1	1.05	0.15	1.05	0.13	6.34E-03	1.08E-02
FFA(22:1)	11.72	5.02	11.83	4.45	6.53E-03	1.11E-02
TAG53:1-FA18:0	0.87	0.95	0.69	0.68	6.64E-03	1.12E-02
DAG(14:0/16:0)	0.43	0.70	0.35	0.57	6.76E-03	1.14E-02
PE(16:0/22:5)	1.46	0.86	1.31	0.72	6.97E-03	1.17E-02
TAG51:2-FA18:0	0.61	0.51	0.52	0.34	6.98E-03	1.17E-02
TAG51:2-FA16:1	1.31	1.36	1.03	0.92	7.18E-03	1.20E-02
FFA(20:4)	4.90	1.28	4.99	1.53	7.28E-03	1.21E-02
TAG58:10-FA22:6	0.61	0.50	0.58	0.70	7.28E-03	1.21E-02
TAG53:3-FA18:1	11.75	5.83	10.32	4.10	7.45E-03	1.24E-02
TAG48:1-FA14:1	0.62	0.79	0.47	0.59	7.51E-03	1.25E-02
TAG51:1-FA18:1	5.32	5.31	4.31	3.98	7.52E-03	1.25E-02
DCER(26:0)	0.14	0.08	0.15	0.08	7.73E-03	1.28E-02

TAG55:8-FA20:4	0.32	0.04	0.32	0.04	7.72E-03	1.28E-02
TAG56:9-FA20:4	0.67	0.58	0.64	0.44	7.79E-03	1.28E-02
TAG50:5-FA16:0	0.43	0.47	0.34	0.36	7.89E-03	1.30E-02
TAG48:3-FA18:2	9.52	9.64	7.42	7.45	7.91E-03	1.30E-02
TAG53:4-FA18:0	0.91	0.07	0.91	0.06	8.18E-03	1.34E-02
TAG51:0-FA17:0	0.61	0.67	0.49	0.51	8.24E-03	1.35E-02
TAG58:9-FA18:2	0.42	0.26	0.38	0.30	8.29E-03	1.35E-02
TAG52:7-FA18:1	0.39	0.36	0.32	0.28	8.53E-03	1.39E-02
CE(20:3)	42.43	24.47	41.06	23.84	8.81E-03	1.43E-02
TAG49:2-FA16:0	3.65	4.18	2.90	2.90	9.09E-03	1.47E-02
TAG53:7-FA18:3	0.24	0.04	0.24	0.03	9.08E-03	1.47E-02
TAG48:1-FA14:0	26.23	29.36	20.74	25.75	9.21E-03	1.49E-02
PE(O-16:0/22:5)	0.93	0.40	0.92	0.40	9.52E-03	1.54E-02
TAG53:1-FA18:1	1.47	1.38	1.21	1.06	9.79E-03	1.58E-02
TAG55:7-FA20:4	2.53	0.24	2.53	0.21	9.93E-03	1.59E-02
TAG58:7-FA20:4	0.25	0.16	0.22	0.13	9.91E-03	1.59E-02
TAG56:6-FA20:4	8.19	4.86	7.14	4.18	1.00E-02	1.61E-02
CE(16:1)	149.82	129.83	142.38	103.74	1.01E-02	1.61E-02
TAG54:7-FA20:4	2.28	1.98	1.92	1.48	1.03E-02	1.65E-02
PI(18:0/16:1)	0.49	0.40	0.47	0.30	1.04E-02	1.66E-02
FFA(24:1)	6.57	2.83	6.70	2.54	1.06E-02	1.68E-02
FFA(14:1)	3.47	5.16	3.62	4.64	1.06E-02	1.68E-02
TAG54:7-FA16:1	0.74	0.60	0.64	0.47	1.07E-02	1.69E-02
TAG50:0-FA14:0	0.48	0.51	0.40	0.45	1.08E-02	1.71E-02
TAG56:3-FA18:1	1.93	1.44	1.62	1.13	1.10E-02	1.74E-02
TAG55:6-FA20:3	1.66	0.16	1.66	0.14	1.11E-02	1.74E-02
TAG52:7-FA22:6	0.22	0.25	0.19	0.23	1.11E-02	1.74E-02
FFA(17:0)	8.87	1.82	8.76	1.62	1.14E-02	1.78E-02
FFA(20:1)	2.36	0.78	2.30	0.61	1.15E-02	1.80E-02
TAG51:0-FA16:0	0.95	1.13	0.75	0.87	1.20E-02	1.88E-02
PC(18:0/22:5)	8.29	3.36	8.43	3.41	1.23E-02	1.92E-02
DAG(14:0/18:3)	0.05	0.04	0.04	0.03	1.25E-02	1.94E-02
LPE(18:2)	1.75	1.10	1.71	1.06	1.25E-02	1.94E-02
TAG48:1-FA18:1	33.10	35.92	26.64	31.63	1.25E-02	1.94E-02
TAG48:0-FA14:0	3.79	4.45	2.98	4.04	1.27E-02	1.96E-02
TAG53:3-FA18:0	6.12	0.43	6.12	0.39	1.28E-02	1.97E-02
TAG49:3-FA16:1	0.75	0.79	0.59	0.49	1.29E-02	1.99E-02
PI(16:0/20:3)	0.67	0.48	0.62	0.32	1.38E-02	2.11E-02
TAG47:0-FA17:0	0.53	0.54	0.46	0.41	1.39E-02	2.13E-02
DAG(15:0/18:2)	0.08	0.06	0.07	0.04	1.43E-02	2.20E-02
DCER(22:1)	0.12	0.03	0.12	0.03	1.45E-02	2.22E-02
TAG51:4-FA15:0	2.05	1.75	1.66	1.16	1.48E-02	2.25E-02
TAG52:6-FA18:1	0.36	0.24	0.30	0.21	1.49E-02	2.26E-02
TAG47:1-FA16:1	0.60	0.90	0.47	0.56	1.49E-02	2.27E-02
TAG55:3-FA18:2	0.36	0.34	0.29	0.22	1.49E-02	2.27E-02

TAG46:1-FA16:1	3.82	5.88	2.75	4.33	1.50E-02	2.28E-02
TAG53:2-FA17:0	4.26	3.30	3.54	2.45	1.52E-02	2.30E-02
TAG49:0-FA17:0	1.01	1.34	0.79	0.98	1.58E-02	2.38E-02
TAG49:2-FA18:2	3.15	3.57	2.53	2.41	1.59E-02	2.40E-02
TAG50:4-FA20:3	0.30	0.33	0.24	0.25	1.64E-02	2.46E-02
TAG53:6-FA18:3	2.25	0.22	2.22	0.19	1.65E-02	2.47E-02
TAG51:0-FA18:0	0.54	0.65	0.43	0.47	1.68E-02	2.52E-02
TAG56:8-FA18:2	2.09	1.40	1.85	1.35	1.71E-02	2.55E-02
TAG50:1-FA14:0	4.47	4.56	3.66	3.84	1.71E-02	2.55E-02
TAG49:3-FA16:0	0.46	0.52	0.36	0.34	1.73E-02	2.57E-02
DAG(14:0/14:0)	0.08	0.09	0.07	0.09	1.81E-02	2.70E-02
TAG45:1-FA18:1	0.18	0.24	0.16	0.20	1.83E-02	2.72E-02
TAG54:1-FA16:0	0.94	0.86	0.81	0.85	1.88E-02	2.79E-02
MAG(15:0)	6.71	38.39	6.71	25.13	1.92E-02	2.84E-02
DCER(22:2)	0.21	0.09	0.21	0.09	2.08E-02	3.07E-02
TAG53:4-FA18:1	14.42	2.02	14.17	1.41	2.09E-02	3.08E-02
TAG48:0-FA18:0	3.08	3.75	2.45	3.44	2.14E-02	3.15E-02
PC(18:0/16:1)	2.09	1.64	1.91	0.99	2.32E-02	3.40E-02
TAG46:0-FA14:0	5.03	7.21	3.82	6.19	2.39E-02	3.51E-02
TAG50:5-FA18:3	2.16	1.87	1.72	1.50	2.43E-02	3.56E-02
PC(16:0/16:0)	61.13	35.32	62.44	36.84	2.47E-02	3.61E-02
TAG52:6-FA16:0	1.11	1.07	0.90	0.94	2.51E-02	3.66E-02
PE(P-18:1/20:4)	11.57	5.16	11.77	5.30	2.55E-02	3.71E-02
DAG(12:0/18:1)	0.12	0.11	0.10	0.10	2.62E-02	3.81E-02
TAG51:3-FA18:3	0.36	0.38	0.29	0.27	2.71E-02	3.93E-02
PE(18:0/18:0)	0.61	0.23	0.62	0.27	2.73E-02	3.96E-02
TAG50:5-FA14:0	1.41	1.28	1.11	1.05	2.75E-02	3.97E-02
TAG55:7-FA22:6	0.21	0.20	0.20	0.20	2.75E-02	3.97E-02
TAG49:2-FA16:1	1.88	2.23	1.46	1.39	2.81E-02	4.04E-02
TAG48:4-FA14:0	0.44	0.49	0.34	0.35	2.86E-02	4.12E-02
TAG48:4-FA18:2	2.30	2.26	1.84	1.96	3.00E-02	4.30E-02
FFA(24:0)	2.80	0.96	2.82	0.88	3.10E-02	4.44E-02
TAG49:3-FA18:2	1.23	1.26	0.98	0.78	3.19E-02	4.56E-02
TAG52:6-FA14:0	0.35	0.34	0.29	0.28	3.21E-02	4.59E-02
TAG53:5-FA18:2	9.36	1.32	9.14	0.93	3.28E-02	4.67E-02
DAG(14:0/20:4)	0.05	0.05	0.04	0.04	3.29E-02	4.68E-02
TAG56:10-FA18:2	0.30	0.28	0.24	0.18	3.34E-02	4.75E-02
TAG51:5-FA18:2	0.37	0.31	0.31	0.22	3.35E-02	4.75E-02
DAG(15:0/18:1)	0.13	0.09	0.11	0.06	3.40E-02	4.80E-02
FFA(22:5)	2.34	0.99	2.26	0.75	3.40E-02	4.80E-02
TAG50:1-FA20:1	0.29	0.30	0.24	0.27	3.44E-02	4.85E-02
TAG53:6-FA18:2	1.40	0.30	1.36	0.23	3.48E-02	4.90E-02

Table S4. Relationship between lactation intensity and fatty acid composition in lipids.

		SAT								ODD		MUFA							PUFA										
Lipid Group	Lipid Class	FA12:0	FA14:0	FA16:0	FA18:0	FA20:0	FA22:0	FA24:0	FA26:0	FA15:0	FA17:0	FA14:1	FA16:1	FA18:1	FA20:1	FA22:1	FA24:1	FA26:1	FA18:2	FA20:2	FA22:2	FA18:3	FA20:3	FA18:4	FA20:4	FA22:4	FA20:5	FA22:5	FA22:6
	Total	-0.15	-0.11	-0.11	-0.03	-0.02	0.02	0.09	0.08	0.01	-0.05	-0.19	-0.17	-0.12	-0.11	-0.01	0.03	0.06	-0.04	-0.13	-0.01	-0.11	-0.09	0.05	-0.02	-0.15	0.18	-0.04	-0.02
Neutral Lipids	MAG			-0.18	-0.27					0.23			0.09	-0.22					-0.18			0.08							
	DAG	-0.22	-0.25	-0.35	-0.34	0.07				-0.14		-0.13	-0.38	-0.39	-0.32				-0.40	-0.27		-0.35	-0.25		-0.27	-0.11	-0.08	-0.40	-0.22
	TAG	-0.18	-0.26	-0.34	-0.30	-0.21				-0.16	-0.24	-0.25	-0.32	-0.28	-0.30	-0.31			-0.34	-0.33		-0.29	-0.26		-0.20	-0.36	-0.01	-0.31	-0.26
	FFA	-0.04	-0.01	-0.09	-0.07	0.02	0.01	0.02		0.05	-0.01	0.03	0.01	-0.04	-0.02	0.04	0.05		-0.04	-0.05	0.01		-0.05	-0.04	0.02	-0.05	0.08	-0.02	0.00
	CE	0.19	0.14	0.04	0.07	0.12	0.20	0.09		0.19	0.12	0.07	-0.02	0.07	0.06	0.12	0.03		0.07	-0.03	-0.01	0.10	-0.04	0.15	0.02	-0.04	0.23	0.03	0.05
Phospholipids	PC		0.09	0.00	0.04	0.12				0.15	0.14		0.02	0.05	0.13				0.03	-0.02		0.06	-0.07		0.00	-0.09	0.23	0.04	0.02
	LPC		0.15	0.08	0.12					0.22	0.19		0.12	0.15					0.15	0.04		0.11	0.02		0.07				
	PE			-0.02	-0.05								-0.04	0.01					-0.04	-0.05			-0.11		-0.04	-0.14	0.22	0.00	-0.02
	LPE			0.06	0.02								-0.01	0.04					-0.02				0.01		-0.01			0.07	0.09
	PI			0.04	0.06								0.12	0.13					0.07				0.01		0.06			0.13	
Sphingolipids	DCER			0.04	0.22	0.20	0.04	0.12	0.09							-0.02	0.05												
	CER		0.05	0.02	-0.09	-0.01	0.08	0.15	0.15							0.05	0.07	0.11											
	HCER		0.14	0.09	0.05	0.11	0.10	0.11						0.12		0.13	0.10												
	LCER		0.19	0.07	0.02	0.05	0.12	0.13						0.02	0.19	0.14	0.15												
	SM		0.13	0.06	0.01	-0.03	0.02	0.09	0.07					-0.04	0.03	0.00	0.03	0.05											

Numbers in the table indicate log2FC, red color denotes $p<0.05$, black color denotes no significance

Table S5. Pathways associated with lactation intensity at baseline.

	Total	Hits	P-value
**Up-regulated lipid species associated pathways**			
Sphingolipid metabolism	21	3	2.11E-03
Glycerophospholipid metabolism	36	3	1.00E-02
Linoleic acid metabolism	5	1	6.30E-02
Biosynthesis of unsaturated fatty acids	36	2	7.67E-02
Arachidonic acid metabolism	36	2	7.67E-02
alpha-Linolenic acid metabolism	13	1	1.56E-01
Glycosylphosphatidylinositol (GPI)-anchor biosynthesis	14	1	1.67E-01
Steroid biosynthesis	42	1	4.25E-01
**Down-regulated lipid species associated pathways**			
Glycerolipid metabolism	16	1	4.07E-02
Steroid biosynthesis	42	1	1.04E-01

Table S6. Potential genes associated with differentially expressed lipid species at baseline.

**Gene list**
GSTA2
ALOX12
EPHX1
ACAA2
CPT2
ST8SIA1
MAN1A2
AGPAT5
HSD17B7
PLCB2
MGLL
ACOT2
CTH
MANEA
GGT5
ABCC1
GOT2
FUT4
SULT1A1
NAT8
OAS3
MINPP1
UGT1A4
COX10
PFKM
HSD3B1
DPEP1
NUDT4
ACSBG1
SPR
AOX1
CSGALNACT1
GDE1
CYP2C9
GPHN
GYS1
CHSY1
ETHE1
DHPS
HMOX1
CARKD
PFKFB1
MAN1A1
MAN1C1

GDA
ACSL3
B4GALNT1
FUT9
SRD5A1
B3GALNT1
IDUA
ACADL
ELOVL3
CAD
UGT8
UGCG
HK3
PGGHG
ST6GAL1
ACSL5
PFKFB3
ERO1B
SLC27A2
DPM1
ALDH4A1
HK1
CYP39A1
IMPA2
OAS1
GYG2
DAGLA
GCLC
ELOVL1
KHK
NUDT3
AKR1C2
NSDHL
MAN1B1
UBIAD1
CES3
AGPAT3
PDHB
CLYBL
GALE
CYP2J2
CERS3
G6PC
DHRS9
FKRP

UGT1A3
HSD17B12
SOAT1
SQRDL
FAR1
RPE65
HADHA
MAN2A2
CYP4F22
GAPDH
HACL1
PDHA1
DIO3
COQ6
NTPCR
B3GALNT2
CRYM
FADS1
FAAH2
LCT
GPI
PLA2G1B
TST
COQ5
CA1
IP6K3
FAAH
IMPA1
ALOX15
ACAA1
COX15
CERS6
BHMT2
CPT1A
DPEP2
SULT2A1
GGCX
FBP2
FECH
ALDOB
MSMO1
HADH
FUT8
ACSBG2
TYR

CHPF
LBR
MCEE
AGPAT4
CBS
DPM3
GALT
PNPLA1
CYP2C19
PFKFB4
PNLIP
QDPR
NANP
GCLM
DPM2
MGEA5
PFKL
ST3GAL2
ACSL4
ALDOC
FUT1
AGPAT1
DDHD1
COQ2
GGT1
SULT1A3
OAS2
GNPAT
ACOX1
PCBD2
NOS2
IMPAD1
FADS2
CYP51A1
NQO1
CCBL2
DLAT
PLCG2
ELOVL7
AANAT
ACADM
NAPEPLD
PPIP5K2
AKR1E2
SULT2B1

SDSL
PFKFB2
CPS1
NUDT10
HK2
CERS4
COQ7
HSD17B10
DYRK1B
SDS
CYP2E1
IP6K1
GYG1
ABO-A
LPCAT4
PTGS1
NOS1
ECI2
GSTZ1
ACOT4
TMEM86B
SOAT2
PPIP5K1
PTGS2
ST3GAL1
HADHB
UGT1A8
CYP7A1
MUT
FUT3
FBP1
G6PC3
CPT1C
ACY3
ECHS1
PLCB1
IDO1
APOA1BP
ABHD4
LTA4H
GBA3
ST6GALNAC1
PLA1A
ELOVL4
ABHD10

PLA2G4A
NOS3
CD38
ACSL1
CSGALNACT2
ACSL6
UGT2B7
MAN2A1
MOCS2
NUDT11
FMO3
DIO2
GSS
SCP2
ENPP2
FKTN
SRD5A2
SUGCT
ALDH9A1
HSD3B2
CYP3A4
UGP2
PDHX
ADH5
PLCE1
ALDH3A2
CYBRD1
GCK
FUT7
SULT1A2
FUT5
FUT6
SLC27A4
DLD
G6PC2
ELOVL5
MTH3
PLA/AT3
RDH5
TECR
CYP1A2
COQ3
PLCD1
PFKP
PECR

FUT2
EHHADH
ALDOA
AGPAT2
PCBD1
ST6GALNAC4
CERS2
CERS5
IP6K2
DPYS
GBGT1
TIGAR
HSD17B4
NAAA
CES1
ALOX5
GPAM
GFPT1
INMT
CYP4F2
CHSY3
DAGLB
LIPE
CYP19A1
MTH1
GALNT1
DBH

Table S7. Differential lipid species between IBF and IFF/Mixed women in the no T2D subgroup.

Lipid species	IFF/Mixed_Mean	IFF/Mixed_SD	IBF_Mean	IBF_SD	P-value	FDR
DAG(16:0/22:5)	0.06	0.04	0.04	0.02	1.04E-26	8.49E-24
DAG(18:0/18:2)	0.71	0.54	0.45	0.29	2.22E-22	9.07E-20
DAG(16:0/18:2)	2.22	1.74	1.39	0.94	3.78E-20	1.03E-17
DAG(18:1/18:2)	6.54	4.40	4.42	2.62	1.54E-19	3.15E-17
DAG(18:2/22:5)	0.10	0.06	0.06	0.04	2.25E-19	3.69E-17
CE(15:0)	8.60	5.69	9.86	5.60	7.68E-19	8.98E-17
DAG(18:1/20:1)	0.10	0.06	0.07	0.03	6.76E-19	8.98E-17
CE(22:0)	0.76	0.45	0.94	0.63	9.99E-19	1.02E-16
TAG56:6-FA22:4	1.71	1.20	1.16	0.79	6.94E-18	6.31E-16
TAG56:4-FA18:0	1.04	0.72	0.75	0.45	3.77E-17	3.09E-15
DAG(16:1/18:2)	0.73	0.58	0.46	0.32	1.68E-16	1.25E-14
LPC(15:0)	0.69	0.29	0.82	0.32	3.92E-16	2.67E-14
DAG(16:0/20:3)	0.12	0.09	0.08	0.04	6.79E-16	4.27E-14
TAG56:5-FA22:4	2.09	1.60	1.42	0.93	1.06E-15	6.17E-14
TAG54:3-FA16:0	6.09	4.54	4.20	3.03	1.26E-15	6.84E-14
DAG(18:1/18:1)	5.80	4.23	4.03	2.21	1.59E-15	8.12E-14
DAG(18:1/22:5)	0.12	0.08	0.08	0.04	2.98E-15	1.35E-13
TAG54:4-FA16:0	7.95	6.04	5.41	3.80	2.95E-15	1.35E-13
DAG(18:1/20:2)	0.11	0.07	0.08	0.03	1.08E-14	4.45E-13
TAG56:5-FA16:0	1.85	1.46	1.25	0.82	1.09E-14	4.45E-13
CE(18:1)	654.75	307.27	720.38	382.59	1.19E-14	4.65E-13
DAG(16:0/18:1)	4.17	3.63	2.62	1.65	1.25E-14	4.65E-13
CE(17:0)	8.92	5.40	9.88	5.42	1.31E-14	4.67E-13
CE(18:2)	1769.17	878.38	1909.14	980.57	1.68E-14	5.71E-13
LPC(17:0)	1.19	0.49	1.36	0.48	2.90E-14	9.50E-13
TAG52:3-FA20:0	0.84	0.57	0.62	0.40	3.47E-14	1.09E-12
TAG52:2-FA18:2	35.34	26.03	25.13	17.12	4.22E-14	1.28E-12
DAG(18:0/18:1)	1.12	0.97	0.72	0.44	8.53E-14	2.49E-12
TAG54:4-FA20:2	2.98	2.11	2.11	1.52	9.27E-14	2.62E-12
TAG56:4-FA16:0	0.41	0.34	0.28	0.20	1.04E-13	2.84E-12
TAG54:5-FA16:0	7.95	6.03	5.58	3.68	1.45E-13	3.83E-12
TAG52:3-FA18:2	278.52	182.20	208.36	131.76	2.16E-13	5.36E-12
TAG56:4-FA22:4	0.25	0.22	0.17	0.12	2.14E-13	5.36E-12
TAG54:2-FA20:2	0.46	0.36	0.32	0.23	2.69E-13	6.48E-12
CER(24:0)	2.80	0.97	3.03	0.98	3.93E-13	9.19E-12
TAG54:5-FA20:3	5.34	3.70	3.83	2.50	4.28E-13	9.72E-12
CE(18:3)	65.53	39.18	72.95	42.37	7.78E-13	1.72E-11
TAG52:3-FA16:0	301.77	197.26	226.36	142.73	8.01E-13	1.72E-11
TAG54:4-FA22:4	0.55	0.57	0.34	0.27	1.51E-12	3.16E-11
LCER(14:0)	0.10	0.04	0.13	0.12	3.05E-12	6.24E-11
CE(18:0)	53.37	27.32	58.52	32.33	3.72E-12	7.42E-11
DAG(16:0/16:1)	0.51	0.59	0.29	0.26	4.42E-12	8.60E-11
CER(26:1)	0.02	0.01	0.03	0.01	5.83E-12	1.11E-10
TAG56:7-FA22:5	4.13	2.67	2.99	1.91	5.94E-12	1.11E-10

PC(17:0/18:1)	2.06	0.73	2.28	0.79	6.47E-12	1.17E-10
TAG54:3-FA20:2	3.70	2.75	2.65	1.82	6.60E-12	1.17E-10
TAG54:5-FA22:5	1.18	1.04	0.78	0.59	6.91E-12	1.20E-10
TAG56:6-FA16:0	3.62	2.57	2.61	1.56	8.23E-12	1.40E-10
TAG56:3-FA18:0	0.51	0.40	0.37	0.23	1.11E-11	1.86E-10
TAG56:6-FA22:5	4.91	3.23	3.61	2.16	1.20E-11	1.96E-10
TAG56:7-FA22:4	0.28	0.19	0.20	0.12	1.63E-11	2.61E-10
TAG52:3-FA18:1	308.56	204.65	233.56	145.33	2.13E-11	3.35E-10
TAG56:5-FA18:0	1.86	1.15	1.43	0.79	2.21E-11	3.40E-10
DAG(18:2/20:3)	0.20	0.12	0.15	0.07	2.37E-11	3.59E-10
PC(15:0/18:2)	1.78	0.71	1.99	0.72	2.68E-11	3.99E-10
TAG52:3-FA22:1	0.20	0.13	0.15	0.09	2.74E-11	4.01E-10
DAG(16:0/16:0)	0.94	0.98	0.54	0.41	2.88E-11	4.12E-10
TAG54:3-FA20:3	0.83	0.68	0.57	0.40	2.92E-11	4.12E-10
TAG54:3-FA20:1	2.30	1.73	1.65	1.12	3.81E-11	5.28E-10
TAG52:2-FA18:0	24.58	19.90	16.97	12.37	4.66E-11	6.35E-10
TAG54:4-FA20:3	6.39	4.86	4.59	2.90	5.48E-11	7.23E-10
TAG56:5-FA22:5	0.62	0.46	0.45	0.29	5.48E-11	7.23E-10
LPC(18:3)	0.28	0.19	0.32	0.18	1.05E-10	1.34E-09
TAG54:5-FA16:1	1.24	0.94	0.88	0.56	1.05E-10	1.34E-09
TAG54:5-FA20:2	0.59	0.41	0.43	0.29	1.18E-10	1.49E-09
TAG50:2-FA18:2	61.97	52.91	41.31	32.69	1.36E-10	1.64E-09
TAG52:3-FA18:0	4.82	3.62	3.43	2.28	1.33E-10	1.64E-09
TAG56:5-FA18:1	5.06	3.38	3.81	2.08	1.36E-10	1.64E-09
TAG52:4-FA22:1	0.33	0.22	0.24	0.15	1.54E-10	1.82E-09
LPC(18:1)	14.42	7.24	15.50	5.12	1.73E-10	2.02E-09
TAG58:6-FA22:4	0.40	0.24	0.31	0.16	1.90E-10	2.19E-09
DAG(16:1/20:2)	0.06	0.03	0.04	0.02	2.60E-10	2.95E-09
TAG54:5-FA22:4	0.59	0.64	0.37	0.29	2.75E-10	3.08E-09
TAG58:7-FA18:2	0.42	0.26	0.32	0.18	2.99E-10	3.31E-09
TAG52:4-FA16:0	129.22	88.91	95.17	65.92	3.18E-10	3.47E-09
CE(24:0)	0.20	0.10	0.23	0.13	3.65E-10	3.93E-09
TAG56:6-FA18:2	3.37	2.21	2.51	1.50	4.05E-10	4.30E-09
TAG54:3-FA18:0	23.89	17.93	17.94	10.72	4.66E-10	4.89E-09
DAG(16:0/20:4)	0.22	0.19	0.14	0.09	5.89E-10	6.10E-09
TAG54:4-FA16:1	0.85	0.62	0.62	0.39	6.59E-10	6.74E-09
DAG(18:2/22:6)	0.09	0.07	0.06	0.07	7.09E-10	7.16E-09
TAG52:4-FA20:2	0.45	0.34	0.32	0.23	7.78E-10	7.76E-09
CE(20:0)	1.27	0.78	1.57	1.31	7.89E-10	7.77E-09
DCER(24:0)	0.57	0.19	0.60	0.21	9.36E-10	9.11E-09
LPC(18:0)	21.83	9.43	23.22	8.06	9.95E-10	9.56E-09
TAG54:2-FA18:2	3.07	2.62	2.24	1.40	1.01E-09	9.56E-09
PE(18:0/22:4)	1.19	0.66	0.90	0.44	1.04E-09	9.64E-09
TAG52:4-FA18:2	206.62	142.90	152.40	104.65	1.04E-09	9.64E-09
DCER(16:0)	0.11	0.04	0.15	0.23	1.24E-09	1.14E-08

DCER(20:0)	0.08	0.03	0.10	0.10	1.53E-09	1.39E-08
TAG56:4-FA20:4	0.57	0.36	0.44	0.23	1.84E-09	1.65E-08
TAG52:4-FA20:0	1.04	0.73	0.77	0.54	2.04E-09	1.81E-08
TAG53:3-FA16:0	1.32	1.10	0.93	0.70	2.21E-09	1.94E-08
SM(14:0)	19.42	6.03	20.82	6.95	2.24E-09	1.95E-08
HCER(20:0)	0.08	0.03	0.09	0.03	2.88E-09	2.48E-08
TAG52:2-FA20:2	0.95	0.89	0.61	0.55	3.37E-09	2.87E-08
PI(18:1/20:4)	1.34	0.66	1.48	0.67	4.56E-09	3.81E-08
TAG54:3-FA18:2	24.36	18.24	18.42	11.12	4.54E-09	3.81E-08
CE(16:0)	417.11	218.50	438.11	229.57	5.23E-09	4.32E-08
TAG52:2-FA16:0	243.63	175.91	184.03	113.21	6.59E-09	5.39E-08
DAG(14:0/18:2)	0.34	0.34	0.21	0.16	7.55E-09	6.06E-08
TAG58:6-FA18:0	0.25	0.16	0.19	0.10	7.51E-09	6.06E-08
LPC(18:2)	21.43	10.56	23.11	8.59	8.03E-09	6.35E-08
TAG52:3-FA20:3	1.69	1.73	1.08	0.90	8.07E-09	6.35E-08
PI(18:1/18:1)	1.86	1.01	2.07	0.92	8.21E-09	6.39E-08
TAG54:2-FA20:1	2.90	2.24	2.11	1.37	9.14E-09	7.05E-08
TAG56:7-FA16:1	0.34	0.21	0.26	0.15	9.47E-09	7.24E-08
TAG50:2-FA16:0	163.94	146.50	109.92	84.63	9.80E-09	7.42E-08
CE(20:5)	24.03	13.92	29.75	22.18	1.01E-08	7.54E-08
CER(26:0)	0.06	0.02	0.06	0.02	1.17E-08	8.58E-08
LPC(14:0)	0.71	0.38	0.76	0.33	1.16E-08	8.58E-08
PC(17:0/18:2)	4.82	1.58	5.23	1.67	1.17E-08	8.58E-08
TAG54:6-FA16:0	4.33	3.19	3.20	2.03	1.22E-08	8.86E-08
TAG56:8-FA22:5	0.66	0.42	0.49	0.31	1.69E-08	1.21E-07
DAG(18:2/20:4)	0.45	0.29	0.34	0.19	1.79E-08	1.27E-07
TAG50:3-FA16:1	44.62	38.52	30.59	22.93	1.94E-08	1.35E-07
TAG56:4-FA20:3	0.84	0.56	0.65	0.36	1.92E-08	1.35E-07
TAG56:7-FA16:0	2.96	1.97	2.25	1.48	1.96E-08	1.36E-07
TAG52:1-FA18:1	51.56	42.41	37.00	24.61	1.99E-08	1.37E-07
TAG54:2-FA16:0	3.26	2.60	2.36	1.63	2.06E-08	1.40E-07
TAG54:1-FA20:1	0.38	0.34	0.27	0.18	2.29E-08	1.55E-07
TAG58:7-FA22:4	0.35	0.22	0.27	0.15	2.37E-08	1.59E-07
CE(24:1)	0.15	0.07	0.15	0.08	2.47E-08	1.64E-07
CE(14:0)	29.23	19.70	31.06	19.83	2.75E-08	1.82E-07
TAG52:3-FA20:2	1.29	1.13	0.88	0.66	2.79E-08	1.83E-07
TAG50:3-FA16:0	49.39	41.96	33.89	25.67	3.05E-08	1.98E-07
TAG50:3-FA18:2	65.50	53.01	46.38	34.19	3.16E-08	2.02E-07
TAG54:6-FA22:5	1.23	1.02	0.87	0.61	3.16E-08	2.02E-07
TAG52:2-FA18:1	388.76	269.29	299.60	179.17	3.92E-08	2.48E-07
TAG58:6-FA18:1	0.60	0.37	0.47	0.25	4.06E-08	2.55E-07
TAG56:3-FA20:2	0.29	0.20	0.22	0.13	4.54E-08	2.84E-07
PC(18:2/18:3)	1.13	0.42	1.27	0.44	5.64E-08	3.50E-07
HCER(24:1)	0.59	0.19	0.65	0.21	5.99E-08	3.68E-07
DAG(16:0/22:6)	0.05	0.04	0.04	0.03	6.71E-08	4.10E-07

CER(22:0)	1.05	0.38	1.08	0.33	6.82E-08	4.13E-07
HCER(24:0)	0.79	0.26	0.85	0.25	6.92E-08	4.16E-07
TAG52:4-FA16:1	35.87	24.60	27.22	17.76	8.52E-08	5.09E-07
TAG56:5-FA18:2	1.85	1.35	1.38	0.86	9.11E-08	5.40E-07
DAG(16:0/18:3)	0.22	0.19	0.15	0.10	9.85E-08	5.76E-07
TAG52:2-FA16:1	6.23	5.03	4.51	2.88	9.80E-08	5.76E-07
DAG(18:0/18:3)	0.09	0.06	0.07	0.05	1.00E-07	5.81E-07
PC(15:0/18:1)	1.18	0.48	1.30	0.54	1.01E-07	5.82E-07
DAG(18:1/20:3)	0.28	0.18	0.21	0.10	1.04E-07	5.96E-07
TAG58:6-FA22:5	0.32	0.19	0.26	0.14	1.12E-07	6.35E-07
TAG54:4-FA18:0	11.12	8.17	8.44	5.62	1.14E-07	6.44E-07
PE(P-18:1/16:0)	0.31	0.13	0.36	0.14	1.17E-07	6.55E-07
TAG58:5-FA18:1	0.21	0.13	0.16	0.09	1.18E-07	6.58E-07
TAG52:1-FA16:0	41.84	35.92	29.39	20.66	1.19E-07	6.59E-07
DAG(14:0/18:1)	0.73	0.69	0.49	0.32	1.26E-07	6.87E-07
PC(18:1/18:1)	16.63	6.25	17.86	6.53	1.26E-07	6.87E-07
TAG52:1-FA20:1	0.75	0.71	0.50	0.44	1.32E-07	7.16E-07
TAG54:4-FA20:1	0.45	0.32	0.34	0.22	1.40E-07	7.55E-07
DAG(16:1/20:4)	0.11	0.11	0.07	0.04	1.54E-07	8.24E-07
PC(16:0/18:3)	9.03	4.76	9.71	4.32	1.80E-07	9.55E-07
CER(24:1)	1.07	0.39	1.11	0.35	2.01E-07	1.06E-06
TAG54:4-FA20:4	2.17	1.73	1.59	0.99	2.03E-07	1.07E-06
DAG(16:1/18:0)	0.25	0.18	0.18	0.11	2.16E-07	1.12E-06
TAG50:1-FA18:1	96.24	91.45	64.07	50.99	2.22E-07	1.14E-06
TAG52:1-FA18:0	31.79	29.22	21.62	15.75	2.20E-07	1.14E-06
TAG50:1-FA16:0	167.50	162.95	109.91	88.86	2.26E-07	1.16E-06
TAG53:4-FA16:0	0.44	0.34	0.32	0.23	2.29E-07	1.17E-06
DAG(18:1/20:4)	0.64	0.43	0.48	0.24	2.46E-07	1.23E-06
HCER(16:0)	0.92	0.28	0.97	0.28	2.45E-07	1.23E-06
LCER(24:1)	0.20	0.08	0.22	0.07	2.55E-07	1.27E-06
LPC(16:1)	2.38	1.22	2.45	0.79	2.62E-07	1.28E-06
PE(P-18:1/18:1)	1.01	0.38	1.11	0.42	2.61E-07	1.28E-06
TAG52:4-FA18:1	52.11	35.68	39.52	24.40	2.58E-07	1.28E-06
CE(18:4)	1.22	0.99	1.38	1.14	2.69E-07	1.31E-06
HCER(22:0)	0.66	0.22	0.70	0.20	2.79E-07	1.35E-06
PC(18:2/18:2)	21.25	8.36	23.02	8.91	2.94E-07	1.41E-06
PC(18:1/20:5)	1.76	1.03	2.14	1.36	2.99E-07	1.43E-06
HCER(22:1)	0.08	0.03	0.08	0.03	3.33E-07	1.58E-06
TAG56:5-FA20:4	4.39	2.51	3.49	1.84	3.59E-07	1.70E-06
PI(18:0/20:4)	20.65	7.64	21.15	6.78	3.79E-07	1.78E-06
TAG54:6-FA20:4	12.42	8.20	9.64	6.10	4.06E-07	1.90E-06
TAG52:1-FA16:1	0.62	0.54	0.44	0.28	5.00E-07	2.32E-06
TAG54:6-FA22:6	1.31	1.26	0.92	0.86	5.84E-07	2.70E-06
TAG56:8-FA16:0	1.11	0.80	0.87	0.73	7.20E-07	3.31E-06
TAG52:3-FA20:1	0.39	0.31	0.29	0.20	7.28E-07	3.33E-06

TAG52:4-FA18:0	0.50	0.37	0.37	0.23	7.96E-07	3.62E-06
TAG56:4-FA18:1	2.87	2.09	2.21	1.27	8.64E-07	3.91E-06
TAG50:4-FA14:1	1.29	1.04	0.97	0.61	9.86E-07	4.43E-06
PC(14:0/18:1)	5.02	2.32	5.26	1.96	1.04E-06	4.64E-06
TAG54:7-FA22:5	0.36	0.26	0.27	0.20	1.09E-06	4.86E-06
LPC(16:0)	83.43	33.82	85.31	25.97	1.18E-06	5.23E-06
TAG54:5-FA20:4	14.58	11.01	11.12	6.86	1.26E-06	5.52E-06
PI(18:0/18:1)	2.11	0.96	2.18	0.84	1.29E-06	5.66E-06
TAG52:4-FA20:3	2.00	1.94	1.36	0.99	1.46E-06	6.36E-06
TAG56:6-FA18:0	1.22	0.71	0.99	0.59	1.51E-06	6.52E-06
TAG52:4-FA22:4	0.26	0.25	0.17	0.13	1.69E-06	7.26E-06
PC(18:0/18:3)	2.92	1.92	3.19	1.79	1.70E-06	7.28E-06
TAG54:2-FA18:0	19.78	17.01	14.81	8.94	1.79E-06	7.63E-06
SM(26:0)	0.50	0.12	0.53	0.14	1.84E-06	7.80E-06
TAG52:2-FA20:1	0.83	0.79	0.57	0.43	1.99E-06	8.34E-06
TAG54:6-FA16:1	1.25	0.88	0.96	0.57	1.98E-06	8.34E-06
TAG55:1-FA16:0	1.18	0.85	0.93	0.78	2.00E-06	8.36E-06
TAG52:5-FA16:1	15.53	10.67	11.72	7.99	2.02E-06	8.39E-06
PC(18:0/18:1)	42.21	15.93	43.52	13.57	2.08E-06	8.58E-06
TAG52:5-FA18:2	32.34	22.44	24.39	17.05	2.32E-06	9.53E-06
SM(16:0)	191.93	46.07	199.53	49.31	2.36E-06	9.67E-06
TAG50:1-FA16:1	7.86	8.55	5.00	4.06	2.44E-06	9.92E-06
TAG52:3-FA16:1	31.85	23.19	24.41	15.44	2.64E-06	1.07E-05
TAG50:2-FA16:1	60.92	61.16	41.19	31.21	2.94E-06	1.19E-05
TAG56:6-FA22:6	0.69	0.59	0.53	0.46	3.12E-06	1.25E-05
DAG(16:0/18:0)	0.79	0.60	0.57	0.29	3.21E-06	1.28E-05
SM(24:0)	33.56	7.44	35.30	8.19	3.55E-06	1.41E-05
TAG54:6-FA20:3	0.95	0.63	0.73	0.44	3.64E-06	1.44E-05
HCER(14:0)	0.02	0.01	0.02	0.01	4.01E-06	1.58E-05
TAG54:3-FA16:1	0.45	0.35	0.34	0.20	4.10E-06	1.60E-05
TAG52:4-FA20:4	3.69	3.94	2.48	2.00	4.29E-06	1.67E-05
PE(P-18:1/18:2)	3.07	1.11	3.43	1.55	4.37E-06	1.69E-05
PI(18:0/18:2)	7.41	2.54	7.65	2.63	4.49E-06	1.73E-05
TAG56:8-FA22:6	4.58	3.72	3.67	3.53	4.90E-06	1.88E-05
LCER(24:0)	0.10	0.04	0.11	0.03	5.04E-06	1.93E-05
TAG56:3-FA16:0	0.19	0.15	0.15	0.14	5.33E-06	2.03E-05
TAG50:0-FA16:0	23.25	24.84	14.58	13.27	6.11E-06	2.31E-05
DAG(18:2/18:3)	0.37	0.26	0.27	0.18	6.38E-06	2.40E-05
LCER(16:0)	2.17	0.59	2.33	0.69	6.41E-06	2.41E-05
PC(18:1/18:3)	1.24	0.61	1.39	0.69	7.31E-06	2.73E-05
CE(20:1)	1.15	0.83	1.20	0.68	7.75E-06	2.88E-05
TAG52:5-FA22:5	0.70	0.57	0.50	0.37	8.13E-06	3.01E-05
PC(18:1/16:1)	10.13	4.04	10.39	3.59	9.02E-06	3.30E-05
PE(P-18:0/20:5)	1.44	1.02	1.75	1.40	8.96E-06	3.30E-05
TAG56:6-FA18:3	0.31	0.20	0.24	0.15	9.03E-06	3.30E-05

TAG53:2-FA16:0	1.64	1.59	1.15	0.89	9.55E-06	3.47E-05
TAG50:4-FA16:1	8.62	7.14	6.16	4.67	9.91E-06	3.59E-05
TAG52:3-FA18:3	3.51	2.69	2.61	1.72	1.01E-05	3.65E-05
PC(14:0/18:2)	7.59	2.76	7.99	2.97	1.02E-05	3.67E-05
TAG52:4-FA18:3	27.91	19.80	21.39	13.55	1.03E-05	3.68E-05
TAG58:7-FA18:0	0.23	0.15	0.18	0.11	1.04E-05	3.70E-05
TAG54:2-FA18:1	36.58	31.74	27.78	16.59	1.13E-05	3.99E-05
LCER(20:1)	0.03	0.01	0.04	0.01	1.19E-05	4.19E-05
SM(24:1)	68.50	16.24	71.16	17.39	1.26E-05	4.41E-05
DAG(18:1/22:6)	0.12	0.09	0.10	0.09	1.27E-05	4.44E-05
PC(18:1/18:2)	85.65	27.91	90.79	32.11	1.28E-05	4.46E-05
TAG56:7-FA22:6	5.27	4.14	4.21	3.63	1.49E-05	5.18E-05
TAG58:10-FA20:5	0.14	0.10	0.17	0.13	1.57E-05	5.41E-05
TAG58:6-FA16:0	0.18	0.14	0.13	0.09	1.82E-05	6.24E-05
PC(16:0/20:1)	1.48	0.47	1.56	0.54	1.91E-05	6.55E-05
TAG52:0-FA16:0	3.57	3.63	2.35	1.88	2.12E-05	7.21E-05
TAG56:6-FA18:1	5.81	3.64	4.67	2.30	2.26E-05	7.69E-05
PC(16:0/18:1)	257.18	93.54	257.61	75.44	2.37E-05	8.01E-05
TAG52:0-FA18:0	4.89	5.11	3.16	2.50	2.53E-05	8.47E-05
TAG52:5-FA16:0	15.76	11.44	11.89	8.62	2.52E-05	8.47E-05
TAG56:1-FA16:0	0.15	0.20	0.10	0.12	2.56E-05	8.56E-05
DAG(18:1/22:4)	0.05	0.03	0.04	0.02	2.59E-05	8.63E-05
PI(16:0/18:1)	1.85	0.99	1.87	0.87	2.82E-05	9.33E-05
PC(18:0/20:5)	7.13	4.11	8.13	4.95	3.04E-05	1.00E-04
TAG48:1-FA16:1	11.49	16.53	6.35	6.42	3.04E-05	1.00E-04
TAG50:4-FA18:2	17.95	14.27	13.40	10.47	3.20E-05	1.05E-04
TAG54:1-FA18:1	4.42	4.46	3.20	2.00	3.47E-05	1.13E-04
TAG53:3-FA18:2	5.34	4.16	4.12	2.69	3.53E-05	1.15E-04
TAG54:4-FA18:2	71.29	52.66	55.64	32.73	3.84E-05	1.24E-04
TAG51:2-FA18:2	3.15	2.81	2.29	1.76	4.12E-05	1.33E-04
TAG54:5-FA22:1	0.37	0.27	0.28	0.18	4.70E-05	1.51E-04
DCER(24:1)	0.39	0.10	0.41	0.13	4.81E-05	1.54E-04
CE(22:5)	3.22	1.35	3.38	1.76	4.97E-05	1.58E-04
PC(18:1/22:5)	1.88	0.75	1.99	0.77	5.15E-05	1.63E-04
HCER(18:0)	0.12	0.04	0.12	0.03	5.24E-05	1.66E-04
LCER(22:0)	0.11	0.04	0.12	0.04	5.28E-05	1.66E-04
TAG58:8-FA18:2	0.53	0.31	0.43	0.23	5.49E-05	1.72E-04
PE(16:0/20:3)	0.56	0.37	0.41	0.21	5.72E-05	1.79E-04
PE(O-18:0/18:2)	0.93	0.33	0.97	0.35	5.98E-05	1.86E-04
TAG51:2-FA16:0	7.63	6.30	5.67	4.04	6.37E-05	1.97E-04
TAG50:3-FA18:1	31.98	27.21	23.75	16.94	6.60E-05	2.04E-04
PC(18:2/20:4)	12.00	3.41	12.47	3.52	6.68E-05	2.05E-04
TAG50:0-FA18:0	8.42	9.50	5.21	5.00	6.69E-05	2.05E-04
TAG56:7-FA18:2	3.85	2.43	3.08	1.70	6.73E-05	2.05E-04
TAG51:2-FA17:0	3.39	2.89	2.48	1.82	7.07E-05	2.15E-04

TAG52:8-FA16:1	0.20	0.22	0.14	0.11	7.32E-05	2.22E-04
PC(18:0/18:2)	272.59	74.14	279.12	78.60	7.40E-05	2.23E-04
TAG50:2-FA18:1	107.37	101.11	76.47	55.27	7.46E-05	2.24E-04
TAG50:3-FA18:3	5.87	5.64	3.99	3.11	7.63E-05	2.29E-04
TAG56:4-FA20:2	0.98	0.67	0.78	0.44	8.44E-05	2.52E-04
TAG52:5-FA20:3	0.57	0.46	0.43	0.31	8.76E-05	2.60E-04
TAG53:2-FA18:0	1.09	0.71	0.89	0.41	9.08E-05	2.69E-04
PE(P-16:0/18:1)	1.24	0.50	1.31	0.48	9.72E-05	2.87E-04
PI(18:1/18:2)	1.27	0.51	1.40	0.70	1.01E-04	2.96E-04
TAG56:2-FA18:0	0.34	0.31	0.26	0.16	1.01E-04	2.96E-04
SM(26:1)	0.68	0.16	0.70	0.17	1.02E-04	2.97E-04
TAG54:1-FA18:0	5.13	5.52	3.57	2.49	1.08E-04	3.13E-04
PE(P-18:1/22:5)	0.96	0.24	1.03	0.35	1.12E-04	3.26E-04
TAG52:8-FA18:2	0.23	0.19	0.17	0.12	1.13E-04	3.26E-04
TAG58:7-FA22:5	0.93	0.50	0.78	0.42	1.20E-04	3.47E-04
DCER(22:0)	0.29	0.09	0.29	0.10	1.27E-04	3.65E-04
PE(P-18:0/18:2)	6.48	2.48	7.14	3.08	1.28E-04	3.66E-04
TAG52:4-FA14:0	0.66	0.61	0.47	0.37	1.31E-04	3.74E-04
PE(16:0/18:1)	1.82	1.18	1.43	0.69	1.33E-04	3.76E-04
TAG54:5-FA18:0	1.61	1.13	1.25	0.85	1.33E-04	3.76E-04
CE(22:1)	0.54	0.49	0.59	0.56	1.36E-04	3.81E-04
PE(18:0/20:4)	19.71	8.86	16.46	6.15	1.35E-04	3.81E-04
PE(P-18:0/18:1)	1.67	0.64	1.78	0.67	1.41E-04	3.94E-04
TAG54:8-FA22:6	0.40	0.37	0.32	0.34	1.49E-04	4.17E-04
DAG(16:1/16:1)	0.14	0.14	0.11	0.21	1.54E-04	4.28E-04
PC(18:2/16:1)	17.07	5.60	17.65	6.08	1.63E-04	4.49E-04
TAG50:4-FA16:0	4.14	4.01	2.88	2.18	1.62E-04	4.49E-04
TAG53:1-FA16:0	0.67	0.78	0.45	0.38	1.66E-04	4.57E-04
TAG53:4-FA18:2	5.09	3.62	4.01	2.52	1.66E-04	4.57E-04
CE(22:6)	13.00	6.07	14.36	8.45	1.70E-04	4.64E-04
TAG54:7-FA22:6	1.38	1.29	1.05	0.95	1.95E-04	5.32E-04
TAG48:3-FA14:1	2.44	2.79	1.59	1.40	1.98E-04	5.38E-04
DAG(14:0/20:0)	0.07	0.03	0.15	0.61	2.02E-04	5.46E-04
TAG53:3-FA17:0	4.98	3.84	3.91	2.49	2.04E-04	5.50E-04
TAG51:3-FA18:2	7.82	6.33	5.97	4.00	2.07E-04	5.57E-04
TAG52:3-FA14:0	0.50	0.46	0.36	0.28	2.10E-04	5.63E-04
TAG50:3-FA14:1	1.41	1.28	1.07	0.69	2.23E-04	5.96E-04
CE(20:2)	2.64	1.37	2.68	1.38	2.32E-04	6.17E-04
TAG54:5-FA18:1	49.03	35.30	38.30	23.66	2.32E-04	6.17E-04
TAG54:4-FA18:1	117.20	85.86	92.54	54.31	2.43E-04	6.44E-04
PE(18:0/20:3)	4.18	2.05	3.41	1.39	2.46E-04	6.49E-04
TAG53:2-FA18:2	0.88	0.79	0.66	0.46	2.67E-04	7.03E-04
PE(O-18:0/18:1)	0.34	0.13	0.36	0.12	2.68E-04	7.04E-04
PI(18:0/20:3)	3.66	1.81	3.54	1.36	2.81E-04	7.35E-04
TAG54:4-FA22:1	0.31	0.23	0.25	0.14	2.82E-04	7.36E-04

TAG52:5-FA18:3	20.06	14.00	15.52	10.57	2.84E-04	7.37E-04
CER(22:1)	0.06	0.02	0.06	0.02	2.93E-04	7.56E-04
TAG51:4-FA16:1	0.58	0.43	0.44	0.29	2.92E-04	7.56E-04
TAG54:5-FA18:2	63.03	48.00	48.69	32.10	3.01E-04	7.73E-04
TAG52:2-FA14:0	0.28	0.27	0.21	0.15	3.02E-04	7.75E-04
TAG51:3-FA17:0	0.92	0.72	0.71	0.49	3.10E-04	7.91E-04
TAG48:0-FA16:0	29.50	38.66	16.99	19.34	3.25E-04	8.28E-04
TAG50:5-FA14:1	0.63	0.44	0.50	0.30	3.28E-04	8.32E-04
LCER(22:1)	0.04	0.01	0.04	0.01	3.42E-04	8.65E-04
TAG56:6-FA20:2	0.45	0.31	0.36	0.22	3.82E-04	9.63E-04
TAG58:8-FA22:5	0.89	0.50	0.74	0.41	3.86E-04	9.71E-04
TAG50:1-FA18:0	8.75	10.03	5.59	4.74	3.89E-04	9.77E-04
HCER(18:1)	0.03	0.01	0.03	0.01	3.98E-04	9.94E-04
PC(17:0/20:4)	2.48	0.85	2.65	1.02	4.02E-04	1.00E-03
DAG(12:0/16:0)	0.16	0.08	0.13	0.07	4.23E-04	1.05E-03
TAG48:2-FA16:1	13.45	18.41	8.14	7.21	4.48E-04	1.11E-03
PC(16:0/20:5)	13.80	7.83	15.22	10.40	4.51E-04	1.11E-03
TAG56:9-FA20:5	0.46	0.30	0.51	0.43	4.59E-04	1.13E-03
TAG50:2-FA20:2	0.34	0.37	0.22	0.23	4.80E-04	1.18E-03
PE(P-16:0/18:2)	4.22	1.74	4.50	1.95	4.86E-04	1.19E-03
TAG54:0-FA18:0	0.83	0.90	0.59	0.38	5.13E-04	1.25E-03
LPE(22:6)	0.15	0.06	0.16	0.07	5.24E-04	1.28E-03
TAG55:4-FA18:1	0.69	0.55	0.54	0.31	5.27E-04	1.28E-03
TAG56:5-FA20:2	0.97	0.70	0.77	0.46	5.41E-04	1.30E-03
TAG56:9-FA22:6	0.73	0.58	0.62	0.61	5.40E-04	1.30E-03
PC(18:2/20:3)	3.51	1.33	3.53	1.22	5.44E-04	1.31E-03
DAG(14:0/16:1)	0.10	0.11	0.06	0.05	5.51E-04	1.32E-03
TAG52:6-FA18:2	2.59	1.77	2.03	1.45	5.74E-04	1.37E-03
TAG48:2-FA16:0	23.09	27.08	14.70	14.03	5.90E-04	1.41E-03
CER(16:0)	0.38	0.12	0.38	0.11	5.94E-04	1.41E-03
PC(16:0/14:0)	3.13	1.64	3.26	1.60	6.12E-04	1.45E-03
TAG50:4-FA14:0	9.23	7.70	7.00	5.60	6.20E-04	1.47E-03
PE(16:0/22:5)	1.49	0.98	1.18	0.58	6.43E-04	1.52E-03
DAG(15:0/18:2)	0.08	0.06	0.06	0.03	6.77E-04	1.59E-03
TAG50:2-FA18:0	2.94	3.00	2.03	1.60	6.87E-04	1.61E-03
TAG56:2-FA16:0	0.20	0.21	0.16	0.27	6.86E-04	1.61E-03
PE(P-16:0/22:5)	3.18	0.83	3.37	1.22	7.02E-04	1.64E-03
TAG48:4-FA16:1	0.61	0.65	0.42	0.35	7.17E-04	1.66E-03
TAG51:3-FA16:1	1.38	1.13	1.03	0.72	7.16E-04	1.66E-03
TAG52:2-FA20:0	0.20	0.16	0.16	0.09	7.57E-04	1.75E-03
FFA(15:0)	6.91	3.82	8.64	9.05	7.86E-04	1.81E-03
TAG48:4-FA14:1	0.44	0.47	0.31	0.25	7.93E-04	1.82E-03
TAG56:7-FA20:5	1.02	0.66	1.08	0.73	8.14E-04	1.86E-03
TAG60:10-FA22:6	0.14	0.10	0.12	0.10	8.15E-04	1.86E-03
TAG52:5-FA20:4	3.82	4.34	2.70	2.06	8.75E-04	1.99E-03

CE(20:4)	254.62	131.03	263.32	144.34	8.83E-04	2.01E-03
TAG55:5-FA18:2	0.39	0.29	0.30	0.20	8.89E-04	2.02E-03
PE(18:2/16:1)	0.54	0.23	0.58	0.28	8.98E-04	2.03E-03
TAG54:4-FA18:3	2.81	2.01	2.21	1.31	9.00E-04	2.03E-03
TAG53:4-FA17:0	2.10	1.56	1.67	1.15	9.09E-04	2.04E-03
SM(22:0)	81.08	22.77	81.43	21.55	9.38E-04	2.10E-03
TAG52:5-FA18:1	4.83	3.32	3.80	2.37	9.50E-04	2.12E-03
FFA(14:0)	21.12	5.86	21.73	5.99	9.99E-04	2.23E-03
LPE(18:1)	1.01	0.57	1.02	0.49	1.00E-03	2.23E-03
TAG58:7-FA22:6	0.44	0.34	0.37	0.32	1.02E-03	2.27E-03
LPC(20:4)	2.63	1.04	2.75	1.03	1.03E-03	2.27E-03
TAG50:2-FA14:1	0.39	0.42	0.28	0.20	1.05E-03	2.32E-03
TAG58:10-FA20:4	0.59	0.40	0.63	0.40	1.05E-03	2.32E-03
TAG48:1-FA16:0	48.48	61.40	29.56	29.42	1.06E-03	2.32E-03
TAG48:3-FA16:1	4.58	4.95	3.08	2.63	1.09E-03	2.37E-03
TAG50:5-FA18:2	1.86	1.44	1.44	1.13	1.09E-03	2.37E-03
TAG49:1-FA16:1	0.66	0.76	0.45	0.41	1.13E-03	2.46E-03
TAG50:3-FA20:3	0.55	0.65	0.36	0.36	1.13E-03	2.46E-03
CER(20:0)	0.14	0.05	0.14	0.05	1.15E-03	2.48E-03
TAG50:5-FA16:1	0.82	0.75	0.60	0.44	1.20E-03	2.59E-03
TAG58:7-FA18:1	0.78	0.44	0.65	0.33	1.23E-03	2.65E-03
TAG48:2-FA18:2	16.45	17.38	11.09	10.63	1.26E-03	2.70E-03
CER(14:0)	0.02	0.01	0.02	0.01	1.29E-03	2.75E-03
PE(18:0/20:5)	1.39	0.87	1.42	0.71	1.29E-03	2.75E-03
TAG58:9-FA22:5	0.44	0.26	0.37	0.21	1.30E-03	2.77E-03
TAG54:3-FA18:1	124.51	93.15	99.87	56.67	1.32E-03	2.80E-03
TAG50:3-FA18:0	0.46	0.44	0.34	0.24	1.36E-03	2.88E-03
TAG51:1-FA17:0	3.79	3.62	2.75	2.18	1.40E-03	2.95E-03
LCER(18:1)	0.15	0.03	0.15	0.03	1.47E-03	3.10E-03
TAG51:2-FA18:0	0.58	0.46	0.46	0.23	1.48E-03	3.11E-03
PE(P-18:0/16:0)	0.33	0.11	0.35	0.12	1.49E-03	3.13E-03
TAG55:4-FA18:2	0.53	0.45	0.41	0.27	1.51E-03	3.16E-03
LPC(20:3)	1.83	0.92	1.81	0.70	1.53E-03	3.19E-03
PE(18:0/22:6)	6.35	3.43	5.60	3.50	1.55E-03	3.23E-03
MAG(18:1)	0.28	0.17	0.23	0.18	1.58E-03	3.29E-03
PI(18:0/22:5)	0.47	0.21	0.48	0.20	1.63E-03	3.38E-03
CE(12:0)	1.03	0.94	1.13	1.05	1.64E-03	3.39E-03
TAG48:2-FA14:0	16.98	18.26	11.35	10.38	1.70E-03	3.51E-03
TAG56:5-FA20:3	2.26	1.39	1.88	0.98	1.73E-03	3.56E-03
MAG(18:2)	0.21	0.09	0.19	0.09	1.74E-03	3.57E-03
PI(16:0/20:4)	2.57	1.82	2.39	1.06	1.79E-03	3.66E-03
TAG50:3-FA14:0	24.14	21.25	18.52	13.77	1.89E-03	3.86E-03
TAG58:7-FA16:0	0.21	0.16	0.16	0.10	2.00E-03	4.07E-03
LPE(16:0)	1.25	0.62	1.21	0.40	2.03E-03	4.13E-03
TAG48:3-FA14:0	4.29	4.33	2.97	2.61	2.04E-03	4.13E-03

TAG56:8-FA16:1	0.29	0.18	0.24	0.16	2.06E-03	4.16E-03
TAG52:5-FA14:0	0.62	0.57	0.46	0.35	2.13E-03	4.30E-03
PE(P-18:0/22:6)	6.30	2.52	6.99	3.79	2.14E-03	4.30E-03
LPE(18:2)	1.68	1.14	1.70	1.05	2.15E-03	4.31E-03
TAG52:6-FA22:6	0.43	0.49	0.31	0.32	2.19E-03	4.37E-03
PE(P-18:1/20:3)	0.77	0.30	0.82	0.38	2.21E-03	4.40E-03
TAG56:8-FA20:5	0.97	0.60	1.05	0.77	2.46E-03	4.90E-03
FFA(20:5)	5.91	1.92	6.17	2.25	2.53E-03	5.02E-03
TAG50:0-FA14:0	0.45	0.52	0.31	0.25	2.77E-03	5.49E-03
TAG51:1-FA16:0	4.78	4.73	3.39	2.76	2.79E-03	5.52E-03
TAG48:1-FA14:1	0.56	0.74	0.35	0.36	2.83E-03	5.58E-03
TAG48:3-FA16:0	3.88	4.39	2.56	2.42	2.88E-03	5.66E-03
TAG48:2-FA14:1	3.68	4.73	2.37	2.24	2.90E-03	5.68E-03
SM(20:1)	7.12	1.60	7.24	2.01	2.91E-03	5.70E-03
PE(P-18:1/22:6)	3.53	1.40	3.88	2.22	2.93E-03	5.71E-03
TAG52:6-FA16:1	2.02	1.41	1.59	1.09	3.11E-03	6.05E-03
PC(20:0/18:1)	1.85	0.93	1.95	0.91	3.18E-03	6.18E-03
TAG54:6-FA18:2	27.87	22.41	21.78	16.65	3.19E-03	6.18E-03
LPE(20:4)	0.78	0.32	0.79	0.31	3.42E-03	6.60E-03
TAG47:1-FA16:1	0.50	0.56	0.36	0.35	3.42E-03	6.60E-03
DAG(16:1/18:3)	0.18	0.15	0.15	0.13	3.49E-03	6.73E-03
TAG58:6-FA20:4	0.15	0.08	0.12	0.06	3.55E-03	6.82E-03
TAG56:4-FA20:1	0.98	0.75	0.78	0.47	3.60E-03	6.89E-03
PE(P-18:0/22:5)	2.22	0.62	2.34	0.84	3.68E-03	7.01E-03
TAG53:0-FA16:0	0.82	0.67	0.64	0.43	3.68E-03	7.01E-03
FFA(22:2)	1.00	0.32	1.02	0.33	3.71E-03	7.07E-03
TAG50:4-FA18:1	3.18	2.66	2.43	1.69	3.83E-03	7.27E-03
TAG49:2-FA16:0	3.33	3.36	2.36	1.93	3.88E-03	7.35E-03
TAG49:3-FA16:1	0.71	0.61	0.53	0.38	3.99E-03	7.54E-03
TAG56:4-FA18:2	1.12	0.86	0.89	0.54	4.02E-03	7.59E-03
TAG55:7-FA15:0	0.32	0.20	0.31	0.15	4.06E-03	7.64E-03
DAG(14:0/20:4)	0.04	0.05	0.03	0.02	4.32E-03	8.11E-03
TAG54:2-FA20:0	0.64	0.51	0.50	0.29	4.34E-03	8.13E-03
TAG47:0-FA17:0	0.47	0.39	0.38	0.28	4.36E-03	8.13E-03
PI(16:0/18:2)	3.08	1.51	3.01	1.32	4.39E-03	8.17E-03
SM(18:0)	33.83	7.89	34.41	10.44	4.40E-03	8.19E-03
TAG56:2-FA20:1	0.32	0.29	0.25	0.15	4.59E-03	8.51E-03
TAG53:3-FA18:1	11.64	5.83	10.06	3.66	4.64E-03	8.59E-03
PC(16:0/18:0)	56.67	33.05	59.11	34.95	4.70E-03	8.67E-03
TAG50:4-FA18:3	6.21	5.69	4.53	3.32	4.91E-03	9.04E-03
TAG46:3-FA16:0	0.39	0.45	0.28	0.33	5.13E-03	9.43E-03
TAG51:2-FA16:1	1.23	1.15	0.91	0.66	5.15E-03	9.44E-03
DAG(14:0/14:0)	0.08	0.09	0.06	0.06	5.17E-03	9.47E-03
TAG51:1-FA18:1	4.88	4.68	3.62	2.78	5.20E-03	9.49E-03
TAG52:7-FA16:0	0.76	0.86	0.51	0.48	5.21E-03	9.49E-03

PC(18:1/20:2)	1.38	0.55	1.38	0.50	5.29E-03	9.62E-03
PC(18:1/22:6)	5.18	1.97	5.39	2.18	5.60E-03	1.02E-02
TAG50:2-FA14:0	21.50	20.21	16.30	11.59	5.78E-03	1.05E-02
LCER(20:0)	0.04	0.01	0.04	0.01	5.92E-03	1.07E-02
TAG53:1-FA17:0	0.84	0.81	0.63	0.43	6.08E-03	1.10E-02
TAG53:1-FA18:0	0.81	0.86	0.58	0.42	6.16E-03	1.11E-02
PC(16:0/18:2)	680.27	177.65	678.02	205.22	6.37E-03	1.14E-02
TAG48:4-FA16:0	0.38	0.45	0.26	0.23	6.45E-03	1.15E-02
TAG49:2-FA18:2	2.92	3.09	2.10	1.74	6.66E-03	1.19E-02
TAG56:7-FA18:3	0.49	0.32	0.39	0.23	6.69E-03	1.19E-02
TAG56:7-FA18:0	0.22	0.13	0.18	0.11	6.72E-03	1.19E-02
TAG56:9-FA20:4	0.61	0.41	0.60	0.38	6.85E-03	1.22E-02
TAG53:2-FA18:1	8.05	6.74	6.40	3.92	7.26E-03	1.29E-02
FFA(20:0)	7.36	1.84	7.71	2.93	7.28E-03	1.29E-02
TAG55:5-FA18:1	0.35	0.21	0.30	0.14	8.07E-03	1.42E-02
TAG52:7-FA22:6	0.19	0.20	0.15	0.15	8.23E-03	1.45E-02
TAG48:1-FA14:0	23.12	26.83	15.19	14.74	8.37E-03	1.47E-02
FFA(20:1)	2.36	0.91	2.34	0.62	8.77E-03	1.54E-02
FFA(14:1)	3.11	2.43	4.16	5.81	8.96E-03	1.57E-02
TAG58:7-FA20:4	0.25	0.14	0.21	0.11	9.02E-03	1.57E-02
TAG55:3-FA18:1	0.93	0.84	0.72	0.44	9.16E-03	1.59E-02
FFA(22:0)	3.73	1.01	3.80	1.07	9.21E-03	1.60E-02
PE(18:0/22:5)	2.23	1.07	1.90	0.73	9.24E-03	1.60E-02
TAG54:7-FA20:4	2.11	1.34	1.74	1.02	9.32E-03	1.61E-02
TAG56:5-FA20:1	0.44	0.33	0.35	0.22	9.34E-03	1.61E-02
DCER(18:0)	0.33	0.86	0.89	3.78	9.37E-03	1.61E-02
LPE(20:3)	0.22	0.12	0.21	0.09	9.43E-03	1.62E-02
LCER(18:0)	0.12	0.03	0.12	0.03	9.47E-03	1.62E-02
TAG48:1-FA18:1	29.53	33.90	19.88	19.44	9.54E-03	1.63E-02
TAG51:4-FA18:2	3.44	2.75	2.72	1.94	9.62E-03	1.64E-02
TAG46:1-FA16:1	3.26	5.29	1.79	2.01	9.68E-03	1.65E-02
TAG58:9-FA20:4	0.77	0.50	0.78	0.47	9.86E-03	1.68E-02
TAG56:3-FA20:1	1.01	0.80	0.81	0.46	9.99E-03	1.69E-02
TAG48:0-FA14:0	3.33	4.09	2.10	2.25	1.01E-02	1.71E-02
TAG55:7-FA20:3	0.22	0.02	0.22	0.02	1.03E-02	1.74E-02
TAG51:0-FA16:0	0.85	0.96	0.58	0.54	1.08E-02	1.82E-02
PE(16:0/20:4)	4.24	2.55	3.46	1.47	1.10E-02	1.84E-02
TAG50:4-FA20:3	0.27	0.30	0.19	0.16	1.10E-02	1.84E-02
TAG52:7-FA18:1	0.35	0.32	0.26	0.19	1.11E-02	1.87E-02
TAG53:1-FA18:1	1.39	1.36	1.04	0.70	1.17E-02	1.96E-02
TAG53:5-FA18:1	6.53	0.51	6.53	0.41	1.18E-02	1.96E-02
FFA(16:1)	17.89	6.90	18.16	6.61	1.21E-02	2.01E-02
TAG51:0-FA18:0	0.49	0.56	0.34	0.31	1.21E-02	2.01E-02
TAG49:2-FA16:1	1.70	1.70	1.25	0.97	1.24E-02	2.05E-02
TAG56:6-FA20:4	8.05	4.51	6.85	3.38	1.24E-02	2.05E-02

PE(O-16:0/22:5)	0.89	0.30	0.91	0.38	1.24E-02	2.05E-02
TAG48:3-FA18:2	8.53	8.60	6.22	5.55	1.26E-02	2.08E-02
DAG(14:0/16:0)	0.35	0.39	0.31	0.65	1.27E-02	2.09E-02
TAG50:1-FA14:0	4.16	4.66	2.93	2.29	1.28E-02	2.11E-02
PC(14:0/20:3)	1.47	0.68	1.45	0.62	1.29E-02	2.12E-02
TAG54:7-FA16:1	0.70	0.47	0.58	0.32	1.30E-02	2.12E-02
TAG51:0-FA17:0	0.55	0.56	0.39	0.34	1.33E-02	2.18E-02
TAG50:5-FA18:1	0.34	0.26	0.27	0.17	1.36E-02	2.21E-02
PE(O-18:0/22:5)	0.57	0.17	0.59	0.21	1.41E-02	2.30E-02
TAG50:5-FA16:0	0.37	0.40	0.27	0.20	1.43E-02	2.31E-02
PC(18:2/20:2)	0.96	0.32	1.01	0.41	1.44E-02	2.32E-02
TAG53:6-FA18:1	1.94	0.13	1.94	0.13	1.43E-02	2.32E-02
TAG52:6-FA18:3	3.10	2.18	2.46	1.75	1.55E-02	2.50E-02
LPE(18:0)	1.22	0.59	1.16	0.36	1.58E-02	2.55E-02
TAG49:0-FA17:0	0.84	0.95	0.59	0.64	1.68E-02	2.70E-02
TAG48:0-FA18:0	2.71	3.43	1.71	2.01	1.76E-02	2.83E-02
DAG(16:1/18:1)	3.50	3.32	2.99	3.08	1.79E-02	2.86E-02
TAG54:1-FA16:0	0.87	0.87	0.66	0.55	1.79E-02	2.87E-02
CE(20:3)	40.50	19.37	40.29	20.97	1.81E-02	2.88E-02
FFA(17:0)	8.83	1.70	8.84	1.72	1.84E-02	2.93E-02
TAG46:0-FA14:0	4.13	5.99	2.38	3.16	1.87E-02	2.98E-02
FFA(22:1)	11.67	4.57	11.73	4.29	1.91E-02	3.03E-02
PC(18:0/18:0)	3.09	1.56	3.08	1.49	1.93E-02	3.05E-02
DAG(15:0/18:1)	0.12	0.10	0.10	0.05	1.99E-02	3.14E-02
TAG49:3-FA16:0	0.40	0.38	0.30	0.23	1.99E-02	3.14E-02
DAG(14:0/18:3)	0.05	0.04	0.04	0.02	2.03E-02	3.19E-02
TAG52:6-FA20:4	1.00	0.87	0.79	0.56	2.03E-02	3.19E-02
TAG53:4-FA18:0	0.90	0.06	0.90	0.06	2.12E-02	3.32E-02
TAG58:9-FA22:6	1.15	0.87	1.09	1.09	2.14E-02	3.35E-02
FFA(20:4)	4.91	1.38	4.94	1.38	2.15E-02	3.35E-02
PE(16:0/18:2)	4.23	2.56	3.52	1.95	2.15E-02	3.35E-02
TAG49:0-FA15:0	0.52	0.61	0.36	0.35	2.16E-02	3.36E-02
PC(16:0/22:5)	25.58	8.02	25.24	8.55	2.17E-02	3.37E-02
TAG58:8-FA22:6	1.23	0.91	1.16	1.09	2.17E-02	3.37E-02
TAG55:6-FA18:1	1.05	0.14	1.04	0.11	2.21E-02	3.42E-02
TAG46:2-FA14:1	0.87	1.45	0.51	0.53	2.26E-02	3.48E-02
CE(16:1)	136.83	80.51	133.47	74.35	2.35E-02	3.62E-02
TAG58:9-FA18:2	0.42	0.25	0.37	0.26	2.37E-02	3.64E-02
FFA(22:5)	2.24	0.75	2.26	0.69	2.37E-02	3.64E-02
CE(14:1)	1.66	1.13	1.69	1.38	2.47E-02	3.79E-02
TAG49:2-FA15:0	2.94	2.84	2.19	1.66	2.48E-02	3.79E-02
TAG49:3-FA18:2	1.16	1.07	0.88	0.66	2.52E-02	3.84E-02
DCER(22:1)	0.12	0.02	0.12	0.03	2.57E-02	3.91E-02
TAG49:1-FA15:0	3.51	3.80	2.57	2.16	2.57E-02	3.91E-02
FFA(20:3)	2.14	0.60	2.13	0.62	2.58E-02	3.91E-02

TAG50:1-FA20:1	0.27	0.30	0.19	0.19	2.58E-02	3.91E-02
TAG51:1-FA18:0	1.01	1.08	0.73	0.56	2.59E-02	3.91E-02
TAG51:2-FA18:1	11.81	10.22	9.34	5.95	2.61E-02	3.94E-02
TAG53:7-FA18:3	0.24	0.03	0.23	0.03	2.75E-02	4.15E-02
TAG56:3-FA18:1	1.88	1.52	1.53	0.87	2.79E-02	4.19E-02
TAG55:8-FA20:4	0.31	0.04	0.31	0.03	2.86E-02	4.29E-02
PC(18:0/16:1)	1.91	1.46	1.76	0.83	3.02E-02	4.51E-02
TAG49:2-FA14:0	0.73	0.76	0.54	0.44	3.02E-02	4.51E-02
PE(16:0/22:6)	5.64	3.82	4.90	3.08	3.07E-02	4.58E-02
TAG50:4-FA20:4	1.02	1.29	0.69	0.66	3.16E-02	4.70E-02
TAG53:2-FA17:0	4.09	3.29	3.32	1.98	3.19E-02	4.74E-02
TAG53:6-FA18:3	2.24	0.20	2.21	0.18	3.25E-02	4.82E-02
TAG54:6-FA18:1	10.07	7.46	8.12	5.58	3.29E-02	4.87E-02

Table S8. Differential lipid species between IBF and IFF/Mixed women in the future T2D subgroup.

Lipid species	IFF/Mixed_Mean	IFF/Mixed_SD	IBF_Mean	IBF_SD	P-value	FDR
CE(14:0)	34.00	29.54	38.32	32.40	8.85E-04	4.23E-02
CER(24:0)	2.84	0.95	3.20	1.09	6.61E-04	4.23E-02
DAG(18:2/22:5)	0.09	0.05	0.07	0.05	7.29E-04	4.23E-02
LPE(22:5)	0.10	0.04	0.12	0.04	1.12E-03	4.23E-02
PC(14:0/18:1)	5.13	2.09	5.76	2.12	4.26E-04	4.23E-02
PC(16:0/14:0)	3.35	1.86	3.87	1.84	4.26E-04	4.23E-02
PC(18:0/20:5)	7.17	3.45	10.14	8.45	6.06E-04	4.23E-02
PE(P-18:1/16:0)	0.31	0.13	0.37	0.13	2.92E-04	4.23E-02
TAG52:3-FA16:0	330.07	227.54	279.10	257.06	1.00E-03	4.23E-02
TAG52:3-FA18:2	302.78	211.73	252.62	221.15	6.86E-04	4.23E-02
TAG52:4-FA16:0	142.59	104.36	115.36	111.00	6.42E-04	4.23E-02
TAG52:4-FA18:1	55.21	38.40	44.98	38.95	8.16E-04	4.23E-02
TAG52:4-FA18:2	219.92	156.00	177.37	164.61	7.63E-04	4.23E-02
TAG52:4-FA22:1	0.35	0.24	0.28	0.25	1.45E-04	4.23E-02
TAG52:5-FA16:1	15.68	11.19	12.24	9.71	1.17E-03	4.23E-02
TAG52:5-FA18:2	34.57	26.04	26.97	24.12	8.65E-04	4.23E-02
TAG54:4-FA20:1	0.48	0.35	0.39	0.34	9.14E-04	4.23E-02
TAG54:4-FA20:2	3.17	2.45	2.61	2.46	8.21E-04	4.23E-02
TAG54:5-FA20:2	0.63	0.50	0.51	0.46	1.19E-03	4.23E-02
TAG56:3-FA18:0	0.56	0.39	0.46	0.40	1.02E-03	4.23E-02
TAG56:5-FA18:2	1.90	1.37	1.50	1.16	1.11E-03	4.23E-02
TAG56:6-FA18:3	0.33	0.24	0.26	0.21	5.92E-04	4.23E-02
TAG58:10-FA20:5	0.15	0.11	0.21	0.20	7.45E-04	4.23E-02
LCER(20:1)	0.03	0.01	0.04	0.01	1.27E-03	4.33E-02
DAG(18:1/18:2)	6.48	4.47	5.12	3.64	1.50E-03	4.53E-02
TAG52:4-FA20:0	1.13	0.86	0.92	0.85	1.47E-03	4.53E-02
TAG56:4-FA18:0	1.12	0.79	0.95	0.79	1.44E-03	4.53E-02
DAG(18:1/22:5)	0.11	0.06	0.10	0.06	1.71E-03	4.64E-02
PC(16:0/20:5)	13.58	6.59	18.64	16.30	1.80E-03	4.64E-02
TAG52:3-FA18:1	333.22	232.63	279.92	244.43	1.59E-03	4.64E-02
TAG52:5-FA16:0	19.28	17.11	15.47	16.82	1.93E-03	4.64E-02
TAG54:4-FA18:0	12.73	9.96	10.03	8.73	1.73E-03	4.64E-02
TAG54:4-FA18:3	3.27	2.48	2.67	2.50	1.85E-03	4.64E-02
TAG55:5-FA18:2	0.48	0.63	0.34	0.28	1.91E-03	4.64E-02
CER(26:0)	0.06	0.03	0.07	0.02	2.36E-03	4.68E-02
DAG(18:0/18:2)	0.75	0.56	0.62	0.55	2.92E-03	4.68E-02
DCER(26:0)	0.14	0.09	0.16	0.10	2.64E-03	4.68E-02
MAG(18:3)	0.19	0.06	0.22	0.06	2.05E-03	4.68E-02
TAG52:4-FA18:3	32.62	24.88	27.56	28.09	2.52E-03	4.68E-02
TAG52:5-FA18:1	5.30	3.87	4.31	3.64	2.40E-03	4.68E-02
TAG52:5-FA18:3	23.72	19.82	19.19	19.16	2.75E-03	4.68E-02
TAG52:6-FA18:3	3.61	3.10	2.79	2.70	2.91E-03	4.68E-02
TAG54:3-FA16:0	6.49	4.68	5.59	5.72	2.91E-03	4.68E-02
TAG54:3-FA20:1	2.47	1.81	2.06	1.88	2.34E-03	4.68E-02

TAG54:4-FA22:1	0.33	0.23	0.26	0.21	2.13E-03	4.68E-02
TAG54:5-FA18:1	53.91	43.62	41.52	33.65	2.49E-03	4.68E-02
TAG54:5-FA18:3	12.11	9.19	9.49	7.84	2.22E-03	4.68E-02
TAG54:5-FA22:1	0.40	0.33	0.30	0.23	2.70E-03	4.68E-02
TAG54:6-FA18:1	11.55	9.98	8.79	7.67	2.90E-03	4.68E-02
TAG54:6-FA18:3	12.08	10.50	9.03	7.97	2.76E-03	4.68E-02
TAG56:5-FA20:1	0.48	0.41	0.36	0.27	2.51E-03	4.68E-02
CE(15:0)	9.51	8.39	10.18	6.76	3.54E-03	4.70E-02
DAG(18:0/18:3)	0.10	0.08	0.08	0.06	3.25E-03	4.70E-02
PC(18:1/20:5)	1.67	0.87	2.30	1.79	3.45E-03	4.70E-02
PE(18:0/20:5)	1.40	0.61	1.73	0.99	3.51E-03	4.70E-02
PI(16:0/18:1)	2.06	1.21	2.19	0.97	3.56E-03	4.70E-02
PI(18:0/18:1)	2.16	0.93	2.36	0.90	3.39E-03	4.70E-02
SM(14:0)	18.74	6.19	20.67	7.01	3.35E-03	4.70E-02
TAG53:4-FA16:0	0.55	0.57	0.45	0.48	3.50E-03	4.70E-02
TAG54:3-FA18:2	27.01	19.41	22.53	20.22	3.13E-03	4.70E-02
TAG54:4-FA18:2	75.74	54.45	60.68	47.62	3.62E-03	4.70E-02
TAG54:5-FA18:2	68.93	59.32	51.51	41.00	3.40E-03	4.70E-02
TAG56:6-FA18:2	3.47	2.42	2.85	2.18	3.58E-03	4.70E-02
DCER(24:0)	0.60	0.24	0.64	0.21	4.31E-03	4.77E-02
PI(18:0/18:2)	7.59	2.69	8.18	2.91	4.15E-03	4.77E-02
TAG52:2-FA18:2	40.01	31.63	34.35	36.91	4.21E-03	4.77E-02
TAG52:3-FA20:0	0.90	0.64	0.77	0.70	4.07E-03	4.77E-02
TAG52:3-FA22:1	0.22	0.14	0.19	0.16	4.29E-03	4.77E-02
TAG52:6-FA18:2	2.84	2.18	2.24	1.96	3.78E-03	4.77E-02
TAG54:3-FA18:0	26.34	18.63	22.23	20.29	3.99E-03	4.77E-02
TAG54:6-FA18:2	33.07	35.21	23.50	21.39	4.26E-03	4.77E-02
TAG55:7-FA15:0	0.33	0.22	0.35	0.23	3.98E-03	4.77E-02
TAG56:4-FA18:1	2.92	1.94	2.43	1.83	3.98E-03	4.77E-02
TAG56:5-FA18:0	1.97	1.22	1.71	1.34	4.26E-03	4.77E-02

Table S9. Differential analytes between IBF and IFF/Mixed women in the no T2D subgroup.

Metabolites	IFF/Mixed_Mean	IFF/Mixed_SD	IBF_Mean	IBF_SD	P-value	FDR
SM (OH) C24:1	1.36	0.40	1.52	0.39	8.29E-12	1.17E-09
lysoPC a C17:0	0.84	0.28	0.98	0.36	2.09E-11	1.48E-09
PC ae C36:1	6.33	1.41	6.98	1.70	1.10E-10	5.15E-09
PC ae C34:1	7.77	1.71	8.51	2.14	8.05E-10	2.84E-08
PC ae C38:2	1.81	0.53	1.99	0.53	2.69E-09	7.57E-08
lysoPC a C28:1	0.40	0.10	0.44	0.11	1.85E-08	3.72E-07
Kynurenine	2.64	0.90	3.33	2.95	1.85E-08	3.72E-07
lysoPC a C18:1	9.79	4.66	10.69	3.70	2.32E-08	4.08E-07
lysoPC a C18:2	19.32	9.01	21.17	7.45	2.63E-08	4.13E-07
lysoPC a C18:0	14.17	5.45	15.56	5.45	1.85E-07	2.20E-06
SM C26:1	0.22	0.10	0.25	0.11	1.79E-07	2.20E-06
Serotonin	0.37	0.49	0.24	0.36	1.87E-07	2.20E-06
PC aa C28:1	2.19	0.56	2.37	0.64	2.93E-07	3.18E-06
SM (OH) C22:1	16.09	3.98	17.39	4.40	3.68E-07	3.70E-06
PC ae C36:2	15.14	3.72	16.60	4.35	6.04E-07	5.68E-06
PC ae C30:0	0.31	0.09	0.34	0.10	7.73E-07	6.81E-06
PC ae C40:2	1.42	0.38	1.53	0.39	1.20E-06	9.97E-06
PC ae C34:0	1.15	0.27	1.26	0.35	1.58E-06	1.24E-05
PC ae C36:0	0.89	0.27	0.95	0.23	1.85E-06	1.37E-05
Orn	66.98	19.70	73.86	25.76	2.87E-06	2.02E-05
PC ae C40:3	0.94	0.19	1.00	0.21	3.92E-06	2.63E-05
SM (OH) C14:1	6.13	1.51	6.58	1.70	5.06E-06	3.25E-05
PC ae C38:0	1.90	0.57	2.11	0.80	2.91E-05	1.78E-04
lysoPC a C24:0	0.15	0.04	0.16	0.05	3.41E-05	2.01E-04
PC ae C44:4	0.23	0.06	0.25	0.06	5.42E-05	3.05E-04
lysoPC a C16:0	55.01	20.91	58.29	18.47	7.66E-05	4.15E-04
Taurine	61.66	40.90	52.18	32.21	1.17E-04	6.13E-04
SM C16:0	115.01	23.06	120.23	27.09	1.30E-04	6.53E-04
Creatinine	73.69	15.94	69.62	14.62	1.41E-04	6.84E-04
lysoPC a C26:0	0.24	0.07	0.27	0.09	2.12E-04	9.99E-04
SM (OH) C16:1	3.83	0.90	4.05	0.98	2.43E-04	1.11E-03
PC aa C36:1	37.48	12.28	39.45	11.10	3.40E-04	1.43E-03
PC aa C36:6	0.71	0.25	0.80	0.39	3.44E-04	1.43E-03
PC ae C34:2	13.44	3.46	14.33	3.81	3.42E-04	1.43E-03
SM C24:1	47.12	11.26	49.86	13.43	3.88E-04	1.56E-03
PC ae C42:3	0.58	0.18	0.60	0.15	4.51E-04	1.76E-03
lysoPC a C26:1	0.11	0.03	0.12	0.05	6.61E-04	2.45E-03
SM C18:1	13.99	3.31	13.37	3.57	6.46E-04	2.45E-03
PC ae C42:2	0.46	0.14	0.48	0.13	6.93E-04	2.51E-03
Gly	256.60	123.94	281.25	131.76	9.29E-04	3.27E-03
PC ae C42:4	0.64	0.19	0.67	0.17	9.78E-04	3.36E-03
SM (OH) C22:2	12.67	3.44	13.23	3.36	1.06E-03	3.55E-03
SM C24:0	21.84	5.73	22.90	5.83	1.48E-03	4.85E-03
SM C26:0	0.20	0.08	0.21	0.10	2.82E-03	9.05E-03

AC4	0.21	0.09	0.23	0.14	3.37E-03	9.56E-03
lysoPC a C16:1	1.89	0.92	1.97	0.72	3.31E-03	9.56E-03
lysoPC a C28:0	0.24	0.09	0.27	0.10	3.27E-03	9.56E-03
PC aa C36:2	288.84	65.63	299.60	70.80	3.28E-03	9.56E-03
PC ae C32:1	2.13	0.52	2.20	0.48	3.39E-03	9.56E-03
His	90.32	28.27	96.14	31.98	3.15E-03	9.56E-03
lysoPC a C20:4	4.56	1.72	4.83	1.66	3.47E-03	9.60E-03
Spermidine	0.20	0.09	0.20	0.06	3.75E-03	1.02E-02
alpha-AAA	1.04	0.48	1.11	0.49	4.39E-03	1.17E-02
PC aa C40:4	3.59	1.29	3.37	1.33	4.65E-03	1.21E-02
Pro	206.93	55.23	216.55	62.36	5.21E-03	1.34E-02
PC aa C24:0	0.07	0.02	0.07	0.03	6.11E-03	1.54E-02
PC aa C32:2	3.99	1.85	4.44	2.18	6.53E-03	1.61E-02
SM C16:1	18.27	3.36	17.80	4.11	8.14E-03	1.95E-02
Val	217.14	55.72	225.28	57.23	8.11E-03	1.95E-02
Thr	134.77	45.16	125.01	37.58	9.29E-03	2.18E-02
PC ae C32:2	0.63	0.17	0.65	0.15	1.05E-02	2.43E-02
Cit	29.33	10.30	32.17	13.06	1.09E-02	2.47E-02
PC aa C40:2	0.27	0.07	0.29	0.09	1.21E-02	2.71E-02
PC aa C36:5	19.95	9.17	21.97	12.71	1.27E-02	2.80E-02
PC aa C34:3	21.43	6.26	22.45	6.61	1.32E-02	2.87E-02
PC aa C30:0	2.78	0.99	2.95	1.14	1.41E-02	3.02E-02
Phe	65.62	10.75	67.93	14.13	1.44E-02	3.02E-02
AC8	0.28	0.08	0.29	0.07	1.53E-02	3.17E-02
Ser	118.79	47.85	128.07	51.57	1.66E-02	3.36E-02
Tyr	72.27	19.63	74.87	20.76	1.67E-02	3.36E-02
PC ae C40:6	3.46	0.89	3.64	1.06	1.77E-02	3.52E-02
PC aa C42:5	0.25	0.07	0.26	0.09	1.95E-02	3.81E-02
PC ae C40:5	2.62	0.65	2.71	0.71	2.39E-02	4.63E-02
Hexose	5566.93	884.07	5510.67	954.48	2.45E-02	4.67E-02
PC aa C42:4	0.15	0.04	0.16	0.05	2.65E-02	4.99E-02

Table S10. Differential analytes between IBF and IFF/Mixed women in the future T2D subgroup.

Metabolites	IFF/Mixed_Mean	IFF/Mixed_SD	IBF_Mean	IBF_SD	P-value	FDR
Kynurenine	2.53	0.72	3.08	1.10	3.66E-06	5.16E-04
PC aa C28:1	2.14	0.56	2.43	0.69	4.06E-04	2.86E-02

Table S11. Baseline clinical characteristics of responders and non-responders in the present study

	Responders to lactation (n=118)	Non-responders to lactation(n=98)	P-value
Prenatal characteristics			
Age, years, Mean (SD)	34.1 (4.5)	32.3 (7.1)	0.29
Pre-pregnancy BMI, kg/m^2, Mean (SD)	32.2 (6.6)	32.3 (7.1)	0.57
Race, n (%)			
Non-Hispanic white	19 (16.1%)	16 (16.3%)	0.52
Asian	38 (32.2%)	32 (32.7%)	
Non-Hispanic black	7 (5.9%)	11 (11.2%)	
Hispanic	54 (45.8%)	39 (39.8%)	
Z-score sum of 3-h 100-g OGTT during pregnancy, Mean (SD)	-0.2 (2.5)	1.5 (3.3)	**0.002**
Treatment for GDM, n (%)			**<0.001**
Diet	82 (69.5%)	42 (42.9%)	
Oral medications/Insulin	36 (30.5%)	56 (57.1%)	
Family history of diabetes			0.378
Yes	64 (54.2%)	59 (60.2%)	
No	54 (45.8%)	39 (39.8%)	
Baseline characteristics at 6-9 weeks postpartum (study baseline)			
2-h 75 g OGTT			
FPG, mmol/l, Mean (SD)	93.5 (7.6)	99.7 (10.2)	**0.011**
2h-PG, mmol/l, Mean (SD)	109.0 (27.9)	130.2 (30.7)	0.107
Fasting Insulin, pmol/l, Median (IQR)	19.5 (13.8-28.8)	23.1 (17.2-35.7)	**0.01**
HOMA-IR, Median (IQR)	4.4 (3.2-6.8)	5.5 (3.9-8.9)	**0.002**
HOMA-β, Median (IQR)	235.3 (176.8-341.9)	247.3 (171.1-354.6)	0.724
Status of Glucose Tolerance, n (%)			**<0.001**
NGT	82	36 (36.7%)	
IFG/IGT	36	62 (63.3%)	

2-month LIR score, Median (IQR)	1.98 (1.88-2.0)	1.98 (1.81-2.0)	0.664
Person-time of follow-up, months, Mean (SD)	70.4 (27.0)	33.3 (25.7)	**<0.001**

Data are presented as Mean (SD) for continuous variables that are approximately normally distributed. Data are presented as Median (IQR) for continuous variables with asymmetrical distributions. Chi-square test was used for categorical variables (n, %), t-test was used for continuous variables (Mean, SD), and Mann-Whitney U test was used for continuous variables (Median, IQR). IBF, intensive breastfeeding; IFF/Mixed, intensive formula feeding or mixed feeding; BMI, body mass index; OGTT, oral glucose tolerance test; GDM, gestational diabetes mellitus; FPG, fasting plasma glucose; 2h-PG, 2-hour post-load plasma glucose; HOMA-IR, homeostatic model assessment for insulin resistance; HOMA-B, homeostatic model assessment for beta cell function; NGT, normal glucose tolerance; IFG, impaired fasting glucose; IGT, impaired glucose tolerance; LIR, lactation intensity/duration ratio; T2D, type 2 diabetes.

Table S12. Predictive performance of 10-analyte signature, non-invasive variables and standard measurements

Variable(s)	Median AUC	95% Confidence Interval	
		Lower Bound	Upper Bound
10-analyte signature	0.78	0.65	0.91
**Non-invasive variables**			
FPG	0.56	0.39	0.73
2hPG	0.62	0.46	0.78
FPG+2hPG	0.60	0.44	0.76
Race/Ethnicity	0.54	0.39	0.73
GDM treatment	0.65	0.52	0.79
Total lactation duration	0.55	0.38	0.71
Family history of diabetes	0.54	0.40	0.68
**Standard measurements**			
Total cholesterol	0.72	0.58	0.86
FFA	0.58	0.42	0.75
HDL-C	0.61	0.45	0.77
LDL-C	0.66	0.51	0.81
TG	0.60	0.43	0.76

AUC, area under the curve; FPG, fasting plasma glucose; 2h-PG, 2-hour post-load plasma glucose; GDM, gestational diabetes mellitus; FFA, free fatty acid; HDL-C, HDL, high-density lipoprotein-cholesterol; LDL, low-density lipoprotein-cholesterol; TG, triacylglycerol.

Table S13. Differential analytes between T2D and no T2D women in the IBF group.

Metabolites	no T2D_Mean	no T2D_SD	T2D_Mean	T2D_SD	P-value	FDR
PC ae C32:2	0.65	0.15	0.61	0.14	8.54E-10	5.14E-08
SM (OH) C16:1	4.05	0.98	3.74	0.99	4.48E-10	5.14E-08
SM C20:2	0.89	0.26	0.80	0.21	1.09E-09	5.14E-08
PC ae C42:5	1.48	0.33	1.39	0.31	3.21E-09	9.07E-08
SM (OH) C22:2	13.23	3.36	12.46	3.19	2.88E-09	9.07E-08
SM C16:0	120.23	27.09	116.25	23.96	2.14E-08	4.31E-07
SM C24:1	49.86	13.43	46.55	10.06	1.87E-08	4.31E-07
SM C18:1	13.37	3.57	12.48	2.90	2.84E-08	5.01E-07
PC ae C36:2	16.60	4.35	15.28	3.82	6.63E-08	8.49E-07
PC ae C44:6	1.16	0.28	1.09	0.26	6.50E-08	8.49E-07
SM C16:1	17.80	4.11	17.20	3.45	6.31E-08	8.49E-07
PC ae C40:4	2.23	0.53	2.12	0.53	7.67E-08	9.01E-07
PC ae C40:2	1.53	0.39	1.44	0.36	1.08E-07	1.18E-06
PC ae C32:1	2.20	0.48	2.13	0.50	3.15E-07	3.17E-06
PC ae C40:3	1.00	0.21	0.98	0.34	4.58E-07	4.30E-06
PC ae C42:3	0.60	0.15	0.58	0.14	6.09E-07	5.37E-06
PC ae C34:2	14.33	3.81	13.56	3.88	1.45E-06	1.12E-05
SM (OH) C24:1	1.52	0.39	1.44	0.39	1.51E-06	1.12E-05
SM C18:0	25.30	6.21	24.24	5.72	1.45E-06	1.12E-05
PC ae C34:1	8.51	2.14	8.17	2.00	1.73E-06	1.22E-05
Creatinine	69.62	14.62	67.63	15.52	2.06E-06	1.38E-05
PC ae C34:3	9.40	3.10	8.52	2.59	3.06E-06	1.96E-05
PC ae C38:4	13.67	3.60	13.03	3.36	3.58E-06	2.20E-05
Hexose	5510.67	954.48	5997.66	977.89	5.97E-06	3.51E-05
PC ae C40:6	3.64	1.06	3.44	1.06	7.50E-06	4.23E-05
PC ae C44:5	0.95	0.25	0.90	0.23	1.36E-05	7.40E-05
PC aa C42:0	0.52	0.13	0.50	0.12	1.94E-05	9.12E-05
PC ae C40:5	2.71	0.71	2.63	0.65	1.87E-05	9.12E-05
SM (OH) C14:1	6.58	1.70	6.36	1.75	1.90E-05	9.12E-05
SM C26:1	0.25	0.11	0.22	0.10	1.94E-05	9.12E-05
PC ae C36:1	6.98	1.70	6.78	1.67	3.01E-05	1.37E-04
PC ae C38:5	17.24	4.67	16.59	4.13	3.43E-05	1.51E-04
PC ae C36:3	8.89	2.56	8.46	2.32	4.82E-05	2.06E-04
lysoPC a C28:0	0.27	0.10	0.25	0.11	8.89E-05	3.69E-04
PC ae C44:4	0.25	0.06	0.24	0.06	1.05E-04	4.24E-04
lysoPC a C28:1	0.44	0.11	0.43	0.15	2.42E-04	9.23E-04
PC ae C36:4	19.64	5.39	19.03	5.25	2.40E-04	9.23E-04
PC ae C42:4	0.67	0.17	0.66	0.17	2.83E-04	1.05E-03
PC ae C36:0	0.95	0.23	0.93	0.21	2.99E-04	1.08E-03
lysoPC a C17:0	0.98	0.36	0.94	0.39	3.83E-04	1.35E-03
AC16	0.12	0.05	0.15	0.05	4.37E-04	1.47E-03
lysoPC a C26:1	0.12	0.05	0.11	0.05	4.30E-04	1.47E-03
PC aa C30:0	2.95	1.14	3.53	1.39	4.82E-04	1.58E-03
Orn	73.86	25.76	70.98	22.24	5.03E-04	1.61E-03

PC ae C34:0	1.26	0.35	1.23	0.37	8.83E-04	2.74E-03
PC ae C38:2	1.99	0.53	1.96	0.56	8.93E-04	2.74E-03
PC aa C40:4	3.37	1.33	3.96	1.49	9.14E-04	2.74E-03
PC aa C32:3	0.62	0.17	0.60	0.15	1.08E-03	3.18E-03
PC ae C30:2	0.08	0.02	0.08	0.03	1.40E-03	4.02E-03
PC aa C38:0	2.66	1.01	2.54	0.86	1.51E-03	4.26E-03
AC18:1	0.12	0.04	0.11	0.04	1.90E-03	5.26E-03
PC aa C32:1	14.72	6.61	17.87	8.35	2.53E-03	6.73E-03
SM (OH) C22:1	17.39	4.40	17.30	4.27	2.50E-03	6.73E-03
alpha-AAA	1.11	0.49	1.05	0.49	3.10E-03	8.10E-03
Val	225.28	57.23	225.38	62.23	4.54E-03	1.16E-02
Putrescine	0.10	0.07	0.09	0.07	4.89E-03	1.21E-02
Taurine	52.18	32.21	48.50	37.09	4.87E-03	1.21E-02
PC aa C38:1	0.73	0.54	0.59	0.59	5.13E-03	1.25E-02
Arg	100.97	47.98	121.54	64.82	5.31E-03	1.27E-02
Phe	67.93	14.13	68.21	14.13	6.04E-03	1.42E-02
PC ae C36:5	12.77	4.16	12.39	3.66	6.71E-03	1.55E-02
Spermidine	0.20	0.06	0.20	0.05	8.59E-03	1.95E-02
PC ae C38:3	4.36	1.15	4.36	1.19	9.48E-03	2.12E-02
t4-OH-Pro	16.88	10.89	15.24	7.45	9.96E-03	2.19E-02
AC8	0.29	0.07	0.28	0.07	1.04E-02	2.22E-02
PC ae C38:0	2.11	0.80	2.07	0.80	1.04E-02	2.22E-02
PC aa C40:5	8.62	3.16	9.82	3.43	1.11E-02	2.35E-02
PC ae C38:6	7.34	2.43	7.22	2.20	1.14E-02	2.36E-02
PC ae C44:3	0.19	0.04	0.19	0.05	1.47E-02	3.01E-02
